# A dispersal of *Homo sapiens* from southern to eastern Africa immediately preceded the out-of-Africa migration

**DOI:** 10.1038/s41598-019-41176-3

**Published:** 2019-03-18

**Authors:** Teresa Rito, Daniel Vieira, Marina Silva, Eduardo Conde-Sousa, Luísa Pereira, Paul Mellars, Martin B. Richards, Pedro Soares

**Affiliations:** 1 Life and Health Sciences Research Institute (ICVS), School of Medicine, University of Minho, 4710-057 Braga, Portugal; 2 ICVS/3B’s, PT Government Associate Laboratory, 4710-057 Braga/4806-909, Guimarães, Portugal; 3 Department of Biology, CBMA (Centre of Molecular and Environmental Biology), University of Minho, 4710-057 Braga, Portugal; 4 Department of Biological and Geographical Sciences, School of Applied Sciences, University of Huddersfield, Queensgate, Huddersfield, HD1 3DH UK; 5 Centre of Mathematics of the University of Porto (CMUP), 4169-007 Porto, Portugal; 6 i3S (Instituto de Investigação e Inovação em Saúde, Universidade do Porto), 4200-135 Porto, Portugal; 7 IPATIMUP (Instituto de Patologia e Imunologia Molecular da Universidade do Porto), 4200-135 Porto, Portugal; 8 Department of Archaeology, University of Cambridge, Cambridge, CB2 3DZ UK; 9 Institute of Science and Innovation for Bio-Sustainability (IB-S), University of Minho, Campus de Gualtar, 4710-057 Braga, Portugal

## Abstract

Africa was the birth-place of *Homo sapiens* and has the earliest evidence for symbolic behaviour and complex technologies. The best-attested early flowering of these distinctive features was in a glacial refuge zone on the southern coast 100–70 ka, with fewer indications in eastern Africa until after 70 ka. Yet it was eastern Africa, not the south, that witnessed the first major demographic expansion, ~70–60 ka, which led to the peopling of the rest of the world. One possible explanation is that important cultural traits were transmitted from south to east at this time. Here we identify a mitochondrial signal of such a dispersal soon after ~70 ka – the only time in the last 200,000 years that humid climate conditions encompassed southern and tropical Africa. This dispersal immediately preceded the out-of-Africa expansions, potentially providing the trigger for these expansions by transmitting significant cultural elements from the southern African refuge.

## Introduction

Africa was the birth-place of *Homo sapiens*. Although evidence suggests limited gene flow with archaic species outside Africa^1^, this statement remains valid on genetic, palaeontological and archaeological grounds. However, one aspect of early modern human evolution where evidence is still scarce is precisely where in Africa *Homo sapiens* emerged. That question might not be answerable using genetic evidence^2^, and the question itself may even be mis-conceived, but eastern, southern and central Africa have in fact all been proposed as the cradle of humanity. Southern Africa has been suggested on the basis of the genome-wide variability of its indigenous “Khoe-San” groups^3^, recently shown using ancient DNA to have extended as far north as Malawi in the Early Holocene^4^; central Africa based on a range of features such as overall mtDNA diversity^5,6^, deep Y-chromosome ancestry^7^ and parasitology and palaeoclimatology^8^; and eastern Africa, mostly on palaeontological grounds^9–11^. A pan-African view^12,13^, or “multi-regionalism within one continent”^5,14,15^, has also gained currency recently, especially given that *Homo sapiens* remains at Jebel Irhoud in Morocco, with similarities to early *Homo sapiens* remains in both eastern and southern Africa, have now been dated to more than 300 ka (300,000 years ago)^16–18^.

A consistent feature that recurs in several distinct lines of genetic evidence is a deep split between hunter-gather/herder “Khoe-San” groups in southern Africa and all other present-day humans (including outside Africa)^4,6,15,19,20^. This split cannot, in itself, distinguish between an origin in the north or the south, but it does establish the existence of two groups of humans early in the evolution of our species. For example, the mitochondrial DNA (mtDNA) genetic clock suggests a coalescence time for all human mtDNA sequences (and therefore maternal lineages) soon after 200 ka^5,21^, and two deep branches in the tree: haplogroup L0 in southern Africa, and the L1′6 branch present in central and eastern Africa (and, ultimately, everywhere else in the world). Genome-wide analyses show the same separation^15,19^, although Y-chromosome sampling and analysis on the current genomic level^22^ are still required for any solid inferences to be drawn.

Furthermore, mtDNA analysis suggests a split within the northern side of the tree (L1′6) between central/western Africa (L1) and eastern Africa (L2′3′4′5′6, or L2′6 for short) dating to 150–135 ka^5^, re-emphasising the emerging picture of deep sub-structuring of early modern human populations within Africa^14^. It is intriguing that the appearance of these three major phylogenetic groupings matches the time at which the use of hafted stone tools (“Mode 3” technologies) became more prominent across the continent^23^. A hypothetical origin for these industries in tropical Africa^24^ could also suggest a major source of modern humans in central Africa before 150 ka^6^, although eastern Africa may also be possible^25^. Such areas would display little gene flow between them, at least until the early Holocene, and drastically later with the Bantu expansion from central Africa^5,26,27^. The first episodes of Middle Stone Age (MSA) genetic exchange between areas detected at the mtDNA level are the emergence of the L2 branch in central/western Africa derived from the deeper eastern African L2′6 haplogroup, ~85–100 ka^26^, the appearance of southern African haplogroup L0 subclades in eastern Africa some time before 70 ka^6^ and the spread of eastern African haplogroup L3 after ~70 ka across eastern Africa, into central Africa and beyond the continent^28,29^.

In parallel with the genetics, there has been much discussion in recent years over the relationship between the origins of *Homo sapiens* and the appearance of distinctively modern behaviour and social features, marked by an increase in symbolic activities and technological complexity in the archaeological record^30^. (A more comprehensive discussion on the archaeology is available in the supplementary text “Archaeological evidence for modern human behaviour” and a map indicating locations and dates of mentioned sites is displayed in Fig. S1.) Marks of such activities, previously associated with the Eurasian Upper Palaeolithic, are apparent much earlier in the southern African archaeological record, especially along the exceptionally rich and economically productive South African coastline^31^, and although potentially an effect of sampling, the location is widely thought to be significant. Blombos Cave, adjacent to the South African coast, is arguably the most impressive site, with an array of modern features (for example, bone tools, and large numbers of deliberately perforated *Nassarius* sea-shell ornaments, red ochre incised with apparently symbolic designs, and most recently the earliest known ochre drawing^32^), with dates of ~100–70 ka, including layers associated with the Still Bay (SB) industry, characterized by distinctively symmetrical, extensively shaped (using pressure flaking on heat-treated silcrete), and bi-facially worked ‘leaf-point’ forms^33^, and an ochre-processing toolkit at ~100 ka^34^. Even earlier occurrences of engraved red ochre are recorded from sites in the Pinnacle Point region (further east on the South African coast), dated to at least 100–150 ka^35^.

These are not completely unique to this region or even to *Homo sapiens*^32^: beads similar to those seen in southern Africa have been found in Morocco at ~82 ka^36^ and even earlier in the Levantine coastal sites of Skhul and Qafzeh, and there are even examples amongst European Neanderthals^37,38^. However, these sporadic occurrences^39–41^ might reflect early, but isolated, elements of cultural modernity which could have been “trumped” by demographic processes, in which these features were subsequently lost due to drift and population extinctions^42^, during times of adverse climate that prevailed in tropical and southern Africa until ~90 ka, and in eastern Africa until ~80 ka^8^. Moreover, there is an underlying taphonomic problem with excavated archaeological sites across Africa: whilst southern Africa is dominated by cave sites, elsewhere open-air sites predominate; and symbolic artefacts, frequently made on organic materials, suffer disproportionately from poor preservation.

The archaeology of eastern Africa for this period lacks similar evidence in the same time frame, but MSA eastern African reconstructions derive primarily from open-air sites, where such evidence is unlikely to be preserved. Nevertheless, some indicators, such as beads and incised ostrich shell, become more common in eastern Africa towards the end of the MSA^43^. A recently reported 78,000-year-long archaeological record at the Panga ya Saidi cave site in Kenya detects the earliest bead between 67–63 ka, followed by a (markedly non-linear) accumulation of novel symbolic and technological behaviors^44^. Furthermore, there is a possibility that southern Africa may have been the source of microlithic technology^45^, established promptly after the Toba eruption ~74 ka^46^, and potentially reflecting the appearance of bow-and-arrow technology and the associated capacity to kill game at a greater distance than possible with purely hand-propelled hunting missiles^47,48^. This appears in the “Howiesons Poort’ (HP) industries, which in stratified archaeological sequences invariably overlie the SB industries, with dates spanning the range from ~67–58 ka^49^. The increased indications of symbolic behaviour are best reflected in the geometrically engraved ostrich eggshell fragments from the site of Diepkloof and Klipdrift Shelter, potentially (by analogy with modern Kalahari San) reflecting marks of personal ownership of egg-shell water containers^50–52^.

Unlike the preceding SB technologies, HP-like microlithic technologies appear further north in East Africa (as at Mumba in Tanzania and in the Naisiusiu Beds at Olduvai Gorge, both showing an almost identical range of “geometric” microlithic forms to those which characterize the South African HP sites), and with associated OSL dates of 56.9 ± 4.8 and 49.1 ± 4.3 for the Mumba industry^53^ and ESR dates of between 62.5 ± 5 and 59.5 ± 5 for the Naisiusiu sites^54^ – broadly contemporaneous with the South African HP sites^43^. A stratigraphic intrusion of backed microlithic forms (predominantly crescents), recorded in layer 11 and 12 at the Kenyan coastal site of Panga ya Saidi cave referred to above and dated broadly to between 58.3 to 44.5 ka^44^, could add further support to the dating of the similar HP-like microlithic industries at the Mumba and Naisiusiu sites. Microliths, defined in the broadest technological terms, were clearly reinvented at several different times and locations within the later (Upper Palaeolithic) time range (as for example in some of the Gravettian and related Kebaran industries in Europe and western Asia). But these later occurrences of microlithic technology in the Eurasian archaeological record show few if any resemblances to the highly distinctive range of microlithic shapes (isosceles triangles, trapezoids, crescents and simpler obliquely blunted forms) which consistently define the much earlier HP-like technologies in both southern and eastern Africa^31^.

The roughly simultaneous appearance of these distinctive HP-like microlithic industries in both southern and eastern Africa suggests that the two phenomena may have been connected by migration, perhaps facilitated by the onset of more humid climatic conditions across tropical Africa ~70–60 ka^8,55^ Since the 95% error ranges overlap substantially, it is not possible to point to direction purely on the basis of the dates, and it remains possible (if statistically unlikely) that they arose independently. In the future, residue, use-wear and macro-fracture analysis could more directly test this suggestion. However, we can test the possibility of a migration with genetic evidence. In fact, though, to date no migration between southern and eastern Africa has been detected in this timeframe^5,28^.

Here, therefore, we reassess the phylogeography of mtDNA L0 lineages in southern Africa and the rest of sub-Saharan Africa, in order to refine our view of early gene flow between continental regions in the MSA. We also analyse genome-wide data for over 1 M SNPs (single-nucleotide polymorphisms) to test the extent to which autosomal evidence can corroborate the mtDNA picture and examine its limitations. We identify, for the first time, a clear signal of dispersal of modern humans from southern to eastern Africa in the time frame of 70–60 ka.

This signal survives only in the maternal record, despite the fact that we show that the overall genetic sub-structuring within Africa indicated by the genome-wide analysis is closely reflected in the mtDNA phylogeny, suggesting that the mtDNA patterns are not misleading. This shows the continued value of modern mtDNA analysis to discern very ancient migration events that can no longer be recovered by genome-wide analysis, and have not yet proven amenable to investigation by means of ancient DNA  (aDNA), due to preservation issues at high time depths in the tropics. This is analogous to the way in which signals in Y-chromosome variation have revealed male-dominated (often pastoralist) dispersals in the mid- to late Holocene that have left no signal in the rest of the genome^56^.

## Results

We constructed a full phylogeny of haplogroup L0 (Table S1) coupled with age estimates calculated using both maximum-likelihood and a whole mtDNA time-dependent molecular clock^21^ and an aDNA calibration that yielded an estimated mutation rate of 2.69 × 10^−8^ [2.06 × 10^−8^; 3.37 × 10^−8^] substitutions per site per year. Ancient DNA calibration with a set of very recent fossil L0 sequences (dating between 1.4 and 6.1 ka) can lead to serious underestimates for ancient divergences such as the ones analysed here, but nevertheless offers an independent calibration and so lower bound of the time of ancient splits. The new data, namely the new structuring of the tree, allowed us to significantly re-evaluate earlier inferences on the timing of the dispersal of L0 from south to north^5,6^.

**Figure 1:**
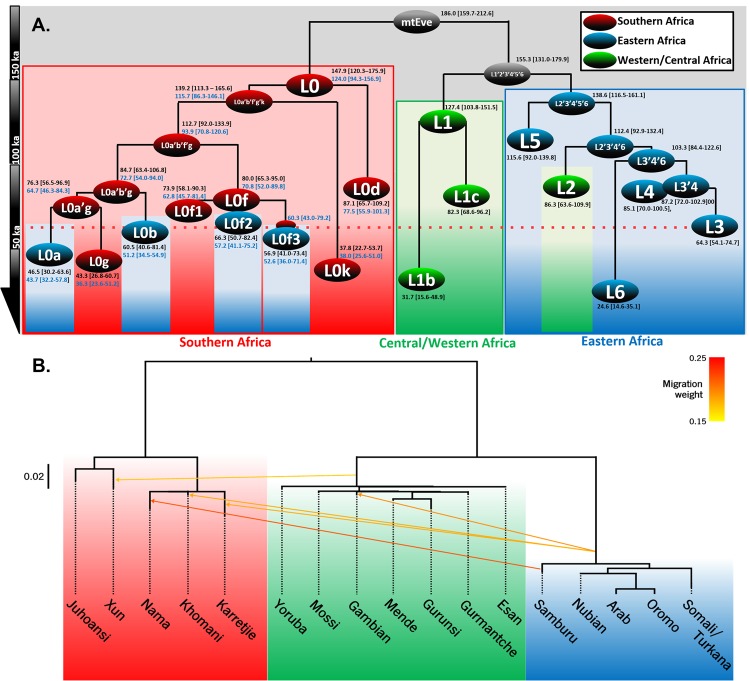
Genetic structure of Africa using mitochondrial DNA and genome-wide data. (**A**) Schematic African mtDNA tree, scaled using maximum likelihood and a time-dependent molecular clock for whole-mtDNA genomes (age estimates in black). Age estimates in blue in L0 were obtained using an ancient DNA calibration with BEAST. (**B**) Maximum-likelihood population tree and admixture events inferred by TreeMix, with five inferred migration edges. The colour of the migration arrows indicates different migration weights. The branch lengths are proportional to the extent of genetic drift that has occurred in each population.

Additional diversity within L0 is now evident: deeper southern subclades within both L0f (both from modern “Khoe-San” samples^57^ and aDNA data^4^) and, more importantly, an entirely new branch, labelled L0g, in southern Khoe-San groups^57^. This shifts the time at which the southern African indigenous haplogroup L0 is restricted to southern Africa from an earlier estimate of ~120–100 ka^6^ to ~70 ka in the new analysis (Fig. 1A). This suggests that all eastern African clades from southern Africa arrived between ~70 ka and ~65–45 ka (based on the age estimates of probable South African nodes L0f, L0a′b′g and L0a′g, and eastern African subclades L0f2′3, L0b and L0a) (Fig. 1A). Most parsimoniously, considering the age of the three founders, it is most likely that their arrival in eastern Africa corresponds to a single event dating to ~70–60 ka. We should note that there is a further eastern African L0 subclade, L0d3b. However, this lineage is most likely related with an independent, more recent, episode of gene flow, within the last 25 ka^6^.

We also performed coalescent simulations, in order to test a range of alternative migration scenarios. They fully rejected models of isolation and gene flow between southern and eastern Africa before 75 ka (Table S2), ruling out a model we had previously proposed^6^. 72.2% of the statistically non-excluded simulations involved gene flow in the time period of interest (i.e., ~75–60 ka) with the remainder concerning the period after 60 ka. While descriptive statistics were unable to clearly distinguish between gene flow from Khoe-San to eastern Africa only and bidirectional gene flow, all simulation models without migrations from Khoe-San to eastern Africa were rejected. Overall the demographic simulations placed an emphasis on migrations between 75–60 ka with obligatory Khoe-San to eastern Africa ancient migrations. However, we should take into account that simulations do not necessarily indicate the best or most probable model but rather they can allow us to reject specific models based on the descriptive statistics, and that here they do allow our hypothesis to stand. Analysing both the simulations and the phylogeographic signal together, it becomes evident that the phylogeography indicates the direction of the migration (southern to eastern Africa) through the polarity of the phylogenetic tree, pointing clearly to a directional movement of L0a′b′g and L0f lineages currently present in eastern Africa but securely nested within a much deeper southern Africa ancestry.

**Figure 2:**
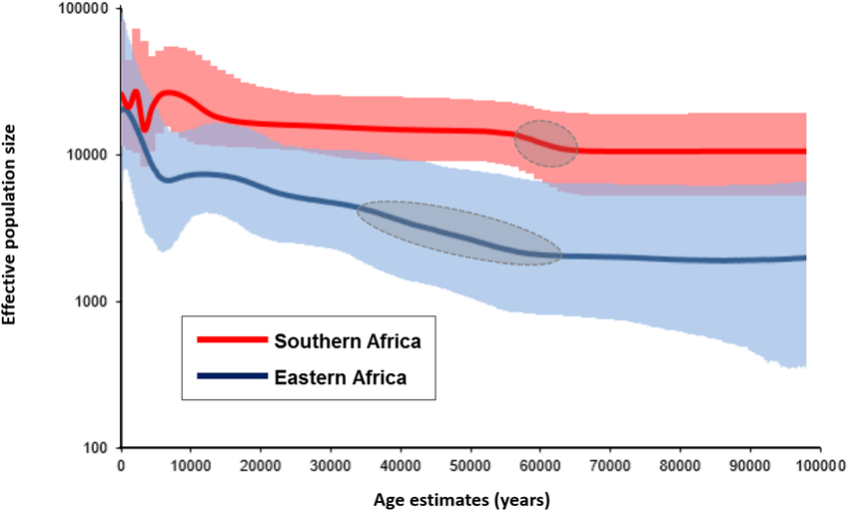
Bayesian skyline plots for haplogroup L0. Blue line corresponds to L0 in eastern Africa and red line corresponds to L0 in southern Africa. Highlighted regions in grey correspond to the probable Middle Stone Age population increments.

We investigated signals of possible population expansions associated with haplogroup L0 in the BSP (Bayesian Skyline Plot) patterns for southern and eastern populations (Fig. 2). For the southern data, which approximates population data from the MSA period, there are no drastic shifts until about 20–15 ka, although there is a mild population increment ~65–55 ka that corresponds to a ~1.4x increment from 10,000 to 14,000 breeding females. For the eastern African data, the major increment is observed with the mid-Holocene, but we can see a long-lasting increment starting at ~60 ka, probably reflecting the arrival time of L0 lineages in eastern Africa. If we consider that these L0 lineages reflect the sub-sample of the southern diversity that migrated north (or the diversity of the migrant population), we could extrapolate from the BSP an effective population size of ~2000 women migrating south to north at ~65–60 ka. This contrasts with the out-of-Africa process, an extremely rapid and dramatic expansion of a very small group of 1000–2000 individuals into effectively virgin territory^5,58,59^. Here we envision the dispersal and relocation of a group within Africa, with only a mild demographic expansion.

We next compared the mtDNA patterns with genome-wide patterns across Africa. Following a preliminary analysis of the overall dataset we excluded the Fulani^60,61^, who had a very divergent pattern whose drifted status was evident in the PCA and ADMIXTURE analyses, and we also excluded the “Coloured_Colesberg” and the “Coloured_Wellington” samples, which corresponded to very heterogeneous groups. In the PCA, we identified and removed six outliers – three Oromo samples and three Somali individuals which have undergone excessive amounts of drift and might therefore skew the analysis. We analysed this modified dataset again using ADMIXTURE, sNMF, PCA and TreeMix.

Cross-validation errors output from ADMIXTURE showed a minimum value at *K* = 4, which is thus the hypothetical ideal value of *K*. The computed component proportions were consistent between ADMIXTURE and sNMF across all values of *K*. As expected, *K* = 2 separates southern Africans from the rest, with variable input from the north as expected in groups impacted by the Bantu expansions^27^, and *K* = 3 clearly separates southern, eastern and central/western components, while the hypothetical optimum *K* = 4 splits the southern component into two (Fig. S2). In the PCA (for 10 major principal components), the first three components explained 71% of the total variance. Projection of PC1 and PC2 shows a clear division between northern (i.e. central/western and eastern African) and southern populations; furthermore, variability among individuals of southern origin is very pronounced, as opposed to the tighter clustering of northern samples along the PC2 axis, especially in the case of Niger-Congo-speaking central/West Africa (Fig. S3). This can be explained, at least in part, by examining the ADMIXTURE analysis, where southern populations display a diverse mixture of the southern and central/western (and to a lesser extent eastern) components, the latter most likely resulting from the Bantu expansion.

Following an initial reconstruction with TreeMix, most of the migration vertices that were added were related to gene flow from central to eastern and southern Africa, representing most likely recent (Bantu) migrations. In order to maximize the detection of earlier migrations, we excluded populations that were a clear mixture of central/western and southern components (mixture of the Khoe-San component with the Bantu component) and a few individuals within populations that showed clear evidence of admixture (unlike the general population). We observed that the populations were successfully clustered according to their geographical location within the continent. More striking was the population tree structure of an initial split between central/western/eastern Africa and southern Africa, with the former then splitting into central/western and eastern Africa – respectively mimicking the splits between mtDNA haplogroups L1′6 and the southern “Khoe-San” L0 and the split of L1′6 into central/western haplogroup L1 and eastern L2′6 in the mitochondrial tree (Fig. 1). This matching between genome-wide data and mtDNA suggests that the maternal line of descent faithfully represents the deep ancient sub-structuring within Africa^14^. The migration edges detected seem to be mostly the result of recent gene flow. Data indicate some gene flow from eastern Africa into some southern groups (visible in the components in ADMIXTURE) but no signal of ancient migration from southern Africa into eastern Africa of any kind is evident.

## Discussion

In the last two decades, remarkable evidence from (in particular) Pinnacle Point and Blombos Cave at the southern Cape of South Africa has accumulated for signs of symbolic behaviour stretching back more than 150 ka. This evidence includes an intensification of selection of red ochre for potentially ritual-based body decoration, sophisticated technological operations such as heat-treatment of lithic raw materials, optimising shell-fishing by tracking lunar phases, and use of shell beads for ornamentation^35,45,50,62,63^ ^(and references therein).^ Most recently, Blombos has yielded the earliest evidence for drawing^32^. These activities initially appeared sporadically, rather than as a package. However, it has been argued that the southern Cape was a refuge throughout the glacial Marine Isotope Stage 6, and a greater intensity seems evident after the end of Stage 6, and especially after 90 ka, as parts of southern Africa likely became wetter and more hospitable and the southern coasts were settled^64–66^. As a result, some have suggested that all later modern humans must, in some sense, descend from a small refugial population of modern humans who lived in southern Africa during Marine Isotope Stage 6, ~200–130 ka^67,68^.

The deep genetic division between Khoe-San groups and other extant humans shows that the idea of a southern refuge is highly plausible – but also that it was not a major genetic source for the rest of humankind. Genetically, present-day modern humans do not descend primarily from southern Africans who lived before ~70 ka – modern African diversity has much deeper roots. In particular, extant mtDNA diversity coalesces around the transition from the inter-glacial Marine Isotope Stage 7 and the glacial Marine Isotope Stage 6, at ~190 ka, and comprises two deep branches, both dating to ~150 ka, one of which arose amongst the ancestors of present-day hunter–gatherer/herder (“Khoe-San”) southern Africans and the other amongst the ancestors of present-day central and eastern Africans. These two deep branches separated between ~190–150 ka, and the northern cluster then split further into a central and an eastern branch. It is from the eastern branch that much African (and all ancient non-African) diversity descends, in the wake of unprecedented demographic expansions, which fuelled dispersals of mtDNA haplogroup L3 both across Africa and out of Africa, after ~70–60 ka^28^. Thus, at least two Stage 6 refuges contributed to modern human mtDNA diversity, but by far the larger contribution was from eastern Africa.

It is widely agreed that the *Homo sapiens* inhabitants of both of these regions would have been cognitively modern – in other words, they would not seem out of place if they were raised in any twenty-first century society. Shea^69^ has argued, for example, that early MSA eastern Africans were as behaviourally flexible as other MSA population where symbolic activities are more evident. It nevertheless remains intriguing that the most impressive evidence for symbolic activities and technological complexity is seen in southern Africa before ~70 ka, and appears in eastern Africa only after 70 ka. This was the time at which microlithic technology appears in both regions almost simultaneously, and was also the only time in the last 135 ka that the climate was simultaneously humid across the whole of the sub-Saharan Africa, so that dispersals became more feasible between what had generally been isolated regions^8^, following the end of the “megadrought” that had kept the tropics largely arid since ~135 ka^44^. Did the southern refuge somehow play a role in the dramatic expansions that led to the peopling of the world?

If this was the case, contact between the two regions at this time may have left a trace in a migration from south to east. Here, indeed, we have described the discovery of a small stream of migrants who moved from southern to eastern Africa around 70–60 ka, during precisely the window of opportunity provided by the climate. Our results suggest it was only during this pan-African wet phase that connections were finally established between southern and eastern Africa with dispersals from south to east.

Signals of MSA migrations between ~135 ka and 70 ka are very scarce in the genetic record. One exception involves the movement of a likely ancestor of haplogroup L2 from eastern to central/western Africa at ~100–85 ka^26^ (Fig. 1A). This represents the most ancient migration detected on the female line of descent following the establishment of specific sub-regional diversities after ~150 ka^5,6^. The dispersal we detect at ~70–60 ka is the next signal that is evident in the mtDNA record, and the first to connect southern and eastern Africa. Aside from a possible later migration that brought L0d3 into Tanzania after 25 ka, it may be the only episode of gene flow between these two regions until the Holocene^5,6^.

While this migration from south to north is clearly visible in the mtDNA record, we do not detect any genome-wide signal. This may be due to the very nature of genome-wide data. In an ADMIXTURE-type analysis of present day individuals^70^, any component arriving ~60 ka would most likely have long since been diluted through recombination into the sink regions’ indigenous components, as suggested by ADMIXTURE analyses of Eurasian aDNA dating to before 20 ka^71^. Even more sophisticated methodologies, such as TreeMix^72^, are unlikely to provide further insight, despite the available resolution of over 1.5 million genome-wide SNPs.

Such deep admixture events would be most readily detected in regions that did not undergo large-scale recombination, and this is where the non-recombining mtDNA and Y-chromosome variation come into their own. Detailed high-resolution genomic Y-chromosome data for eastern Africa and southern Khoe-San populations are still lacking, although we must note that, in any case, Y-chromosome results will not necessarily match mtDNA patterns, as males and females can have very different populations histories – and deep ancestry seems to be more often over-written on the male line of descent, further exacerbating the problem of limited sampling^73^. Nevertheless, despite the inability of the genome-wide analyses to detect the deep migratory events, the early separations and dispersals established a deep genome-wide African population structure that closely matches the mtDNA phylogeography and the split of human diversity into three major groups (southern African, central/western African, and eastern African/non-African). This supports the deep L0 southern African ancestry and, consequently, the presence of more recent L0 subclades in eastern Africa can only be explained by gene flow from southern to eastern Africa.

Several archaeologists have proposed that developments during the Still Bay and Howiesons Poort phase of the MSA maybe have contributed to the out-of-Africa expansions^31,47,74^. It is therefore tempting to speculate that the migrants at 70–60 ka might have transmitted innovations developed in the southern refuge, such as microlithic technology or even novel symbolic activities, to eastern Africa – innovations that may have had a role in the subsequent unprecedented expansions of eastern African populations. The number of migrants from the south was probably not very large, as reflected in the present-day frequencies of L0 in eastern Africa of 5–15% on average^5^. Technologies and culture are readily transmitted horizontally, and a relatively minor genetic influx (involving several thousand individuals at most) may have sufficed to transmit the cultural innovations to eastern Africans ~70–60 ka, and seed the populations that migrated outside the continent. In genetic terms, the earliest out-of Africa populations might even have carried a low level of southern African ancestry, but the out-of-Africa bottleneck was very drastic, with the maternal lineage of all humans outside Africa eventually deriving from a single (and likely random) eastern African sequence, the root of haplogroup L3, and any hypothetical genome-wide South African ancestry was diluted through recombination.

It is possible that both the dispersal south to north and the out-of-Africa expansion are independent by-products of the shift in climate after 70 ka, and that the simultaneous appearance of the microliths is a coincidence or an artefact of the scanty archaeological evidence in eastern Africa. On the other hand, if the connections were causal, it would imply that a seemingly minor re-evaluation of the mtDNA tree in Africa may have significant implications for the evolution of *Homo sapiens*. A cluster of *Homo sapiens* populations, living in a glacial refuge area on the southern coast of Africa, developed a sophisticated repertoire of complex technological and symbolic behaviours over many tens of thousands of years^75,76^. This was already evident ~165 ka at Pinnacle Point and reached a zenith at sites such as Blombos by ~80–70 ka. These developments were far from unique (with even some indications amongst other hominin species such as Neanderthals^37,38^): similar developments took place at other sites across the range of early *Homo sapiens* in North Africa and the Near East, who likely had similar cognitive capacities. However, these were lost or dispersed southwards due to the onset of harsher climatic conditions in the north after 75 ka^39–41^ – there may even have been a “corridor” connecting these regions via the eastern African coastline prior to this time^66^. Populations may well have moved rather than simply becoming extinct – indeed, population shifts seem to characterise the MSA^66^. At the same time, however, favourable climate conditions in the tropics and the south and demographic expansion led to the innovations that emerged in southern Africa being fixed in an expanding population, resulting in them being transmitted to other human groups in, ultimately, eastern Africa. Thus demography likely played a critical role, re-shaping cognitive capacities that may have been in place for perhaps half a million years^37^.

Thus the use of beads, incised ochre, heat treatment and possibly microlithic technologies, might then potentially have been transmitted to eastern Africa after 70 ka, when – even considering a sampling bias for previous periods – some of these elements become more common in the archaeological record^43^. As well as the appearance of microlithic tools, the earliest bead in a 78,000 year-long archaeological record in Kenya appears at ~67–63 ka alongside a shift in toolkits^44^. This migration from southern to eastern Africa after 70 ka was closely followed by a major demographic expansion that is clearly visible in both the genetic and archaeological records^77^, marked by the decreasing reliance on diagnostic MSA technologies characteristic of the MSA–LSA transition. While the microlithic evidence for a southern source into eastern Africa can be contested on statistical grounds, the putative scenario described for the migration of people and behavioural elements from southern to eastern Africa at the 70–60 ka time frame would be consistent with the concomitant transmission of a technology which clearly appears in both regions at this time. This spread likely involved a small group of migrants from southern Africa, leading not to population replacement or an immediate acculturation across indigenous eastern African populations after 70 ka, but creating a patchwork distribution in both time and space, consistent with the heterogeneity of the archaeological record^44^.

By 60,000 years ago, however, a fully “syntactic” language akin to those used today^74^ must have been widespread across both southern and eastern Africa, and have been carried out of Africa. The inference from various aspects of the record to “symbol use” and from there to “fully syntactical language” is far from straightforward^63,74^. However, the fact that a phenomenon is imperfectly understood does not mean it should be dismissed^78^, especially when it as potentially significant as charting the emergence of people who potentially were beginning to communicate in ways approaching present-day humans. Indeed, linguists and archaeologists have begun to address the theoretical challenges^63,74^, and it is possible to sketch out plausible trajectories from courtship rituals shaped by sexual selection into symbolic activity and language, over a likely timeframe of hundreds of thousands of years^79–81^.

Even so, there has been growing scepticism amongst archaeologists in recent years against the possibility that human symbolic behaviour radiated outwards from a single source in southern Africa at such a recent date. By providing novel genetic evidence for a dispersal at this time, we show that a transmission mechanism existed. If symbolic behaviour first gained a foothold primarily in the south, this dispersal could have transmitted it to the rest of Africa by migration and subsequent acculturation.

The populations in eastern Africa that resulted from the arrival of new groups from the south were the starting point for the greatest expansion ever undergone by modern humans, not only back across Africa but also out of Africa, along the Indian Ocean into South Asia, Southeast Asia and Australasia, and ultimately to the rest of the globe. Interpreted in this way, the archaeological and genetic evidence concur that southern Africa is a plausible candidate for the cradle of modern humankind.

## Materials and Methods

### mtDNA phylogenetics

We built an updated phylogeny of mtDNA haplogroup L0 containing 1024 sequences (Table S3). We used the reduced-median algorithm^82^ to build an initial network that was further manually checked and refined using the relative mutation rate of the different mtDNA positions^21^. We selected 204 further African sequences to represent the rest of the African mtDNA tree^5^ (Table S4).

We estimated ages of the overall African tree using maximum-likelihood^83^ and the time-dependent molecular clock we developed for the whole human mtDNA^21^. For haplogroup L0, we also estimated ages using an aDNA calibration with BEAST^84^. We used sequences that were either from haplogroup L0 or clearly external to the L0 and African phylogeny for a total of 10 aDNA mitogenomes. The internal L0 sequences were from haplogroups L0d2c1 (KJ669158)^57^, L0d1c (I4468), L0d1b2b (I4427), L0a1′4 (I1048), pre-L0k2 (I4421), L0k1 (I4422) and pre-L0f3 (I4426)^4^, all dating between 1.4 and 6.1 ka. Some L0 sequences from the work by Skoglund and collaborators^4^ were excluded, due to large gaps present in the sequences. External sequences included a pre-N sequence (Oase1) dating to 37.8 ka^85^, a Neanderthal sequence (KC879692) dating 50.3 ka^86^ and a Denisovan sequence (FR695060) dating to ~50 ka^87^. We built BSPs, using the same aDNA calibration parameters, for the southern African L0 data (excluding L0a sequences that are most likely related with the later Bantu migration into the south of the continent)^6,26^, and by using the L0a′b′f data from the eastern African specific clades. The first dataset, the southern sequences, approximates population data, as no other clades have been detected to date that could trace into MSA southern Africa.

We performed coalescent simulations using the software ms^88^, in order to test the validity of the model proposed here. We took several models into account, including total isolation following the split between eastern and southern Africa, models that consider both unidirectional and bidirectional gene flow in the periods between 60 and 75 ka, in the last 60 ka, and before 75 ka. Simulations were run in the cluster SEARCH from the University of Minho. For populations in the simulations we used the ancient southern L0 data as an approximation of ancient Khoe-San mtDNA gene pool and the 1000 Genomes Project Luhya data to represent eastern African population. We estimated changes in effective population size through time as Bayesian skyline plots in BEAST^84^. We describe details of the analysis in the Supplementary material text (Coalescent simulations) and Table S5.

### Genome-wide analysis

For the selection of the dataset, in order to investigate deeper evolutionary patterns in African populations, we opted for maximizing the number of SNPs in the analysis (Table S6). We initially merged 220 samples from 11 southern African populations^15^ with 161 individuals spanning 13 Sahelian groups^60^. Both datasets contained genotype data for over 2.2 million SNPs, of which 2,032,247 were found to overlap. We included an additional 170 samples from the five African populations of the 1000 Genomes Project. Upon excluding SNPs showing strand inconsistencies, we kept over 1.5 M SNPs (1,558,657 polymorphisms) in the merged dataset. The aim of the analysis was to test if we could identify signals of MSA gene flow on the genome-wide level using TreeMix^72^ with the support of ADMIXTURE^89^ and PCA^90^ for data characterization.

We carried out ancestry analysis by running ADMIXTURE^89^ and sNMF^91^ for values of *K* between 2 and 10 with the calculation of cross-validation errors in ADMIXTURE, and performed a PCA for 10 major principal components (using EIGENSOFT 6.1)^90^. We assessed population splits and possible migrations using TreeMix. We used 100 bootstrap replicates to generate a consensus tree based on the African populations, to be used as scaffold during a posterior run in which six migration edges were added. In all cases, we performed standard error calculations using blocks of 100 SNPs. For TreeMix, we aimed to maximize ancient admixture processes in terms of migration edges, by minimizing clear recent processes of admixture supported by the three types of analyses. To that end, we excluded groups that were recently established from two ancestral populations as well as some individuals that showed recent double ancestry within otherwise non-mixed groups, in a similar fashion to that performed before^15^.

## Supplementary information

Supplementary material

Supplementary dataset 1
