# Supplementary material for: A dispersal of Homo sapiens from southern to eastern Africa immediately preceded the out-of-Africa migration
Source: Sci Rep. 2019 Mar 18;9:4728. doi: 10.1038/s41598-019-41176-3 (PMC6426877; doi:10.1038/s41598-019-41176-3)
Supplement: Supplementary file 1 — Supplementary material [file 41598_2019_41176_MOESM1_ESM.docx]

**Supplementary material for:**

**A dispersal of *Homo sapiens* from southern to eastern Africa immediately preceded the out-of-Africa migration**

Teresa Rito^a,b,^*, Daniel Vieira^c,^*, Marina Silva^d^, Eduardo Conde-Sousa^c,e^, Luísa Pereira^f,g^, Paul Mellars^h^, Martin B. Richards^d,1^, Pedro Soares^c,i,1^

*joint first authors

*^a^Life and Health Sciences Research Institute (ICVS), School of Medicine, University of Minho, 4710-057 Braga, Portugal*

*^b^ICVS/3B's, PT Government Associate Laboratory, 4710-057 Braga/4806-909 Guimarães, Portugal*

*^c^Department of Biology, CBMA (Centre of Molecular and Environmental Biology), University of Minho, 4710-057 Braga, Portugal*

*^d^Department of Biological and Geographical Sciences, School of Applied Sciences, University of Huddersfield, Queensgate, Huddersfield, HD1 3DH, UK*

*^e^Centre of Mathematics of the University of Porto (CMUP), 4169-007 Porto, Portugal*

*^f^i3S (Instituto de Investigação e Inovação em Saúde, Universidade do Porto), 4200-135 Porto, Portugal*

*^g^IPATIMUP (Instituto de Patologia e Imunologia Molecular da Universidade do Porto), 4200-135 Porto, Portugal*

*^h^Department of Archaeology, University of Cambridge, Cambridge CB2 3DZ, UK*

*^i^Institute of Science and Innovation for Bio-Sustainability (IB-S), University of Minho, Campus de Gualtar, 4710-057 Braga, Portugal*

^1^To whom correspondence should be addressed:

Martin B. Richards, Department of Biological and Geographical Sciences, School of Applied Sciences, University of Huddersfield, Queensgate, Huddersfield, HD1 3DH, UK; phone number +44 1484 471676; email: [m.b.richards@hud.ac.uk](mailto:m.b.richards@hud.ac.uk)

Pedro Soares, CBMA (Centre of Molecular and Environmental Biology), Department of Biology, University of Minho, Campus de Gualtar, 4710-057 Braga, Portugal; phone number +351 93 866 9129; email: [pedrosoares@bio.uminho.pt](mailto:pedrosoares@bio.uminho.pt)

**Archaeological evidence for “modern human behaviour”**

In the recent evolutionary literature, the concept of “anatomically modern” humans has become closely intertwined with the notions of “behaviourally modern” populations, for which the only direct source of evidence comes from the associated archaeological records of the populations involved. While some authors[^1^](#_ENREF_1) have argued that the very concept of “behavioural modernity” or “cultural modernity”is at best very difficult to define, arguably the most simple definition of “behaviourally modern” features would be features which are found in most if not all anatomically modern (*Homo sapiens*) populations but, with some rare and controversial exceptions[^2^](#_ENREF_2), not found in earlier “archaic” populations, such as those of the European and western Asian Neanderthals (*Homo neanderthalensis*) and potentially closely related populations in eastern Eurasia. Some of the clearest and most sharply defined behavioural features in this context are those of distinctively symbolic (e.g. “artistic” and “decorative”) expression, together with more technologically “advanced” features, such as complex microblade and associated microlithic technologies, systematically and extensively shaped bone and antler artefacts, or stone tools showing a clear pattern of “imposed form” or “style” in their visual appearance and design[^3^](#_ENREF_3)^,^ [^4^](#_ENREF_4)^,^ [^5^](#_ENREF_5)^,^ [^6^](#_ENREF_6). In Europe, these features appear relatively abruptly with the replacement of Neanderthal by anatomically modern *Homo sapiens* populations, equipped with a range of complex and rapidly changing “Upper Palaeolithic” technologies.

Whether judged by these or any other plausible criteria, there is wide agreement that the most impressive early appearance of more complex and “advanced” forms of cultural and symbolic behaviour can be documented from a range of Middle Stone Age (MSA) sites in the most southerly regions of Africa, particularly along the ecologically and economically exceptionally rich maritime environments around the South African coastline, where the documented levels of marine productivity are exceptionally high – with the associated potential to support commensurately high densities of human populations[^7^](#_ENREF_7)^,^ [^8^](#_ENREF_8)^,^ [^9^](#_ENREF_9) (Figure S1).

At present, the clearest and most explicit evidence for a relatively sudden burst of cultural and technological innovation is provided by the Still Bay (SB) levels in the Blombos Cave, located close to the extreme southern tip of South Africa, and dated by a range of techniques to between ~100 and 75 ka[^9^](#_ENREF_9). In terms of technology, these industries are best defined by a range of highly symmetrical and carefully shaped bifacially-retouched “leaf point” forms. Although varying in size, these forms are generally assumed to represent the tips of wooden hunting spears. Arguably more significant in these levels is the presence of carefully shaped bone points, together with large quantities of red ochre, many of which show clearly deliberate, and apparently symbolic, engravings on their prepared surfaces[^5^](#_ENREF_5), and even what are clearly drawings[^10^](#_ENREF_10), along with large numbers of deliberately perforated sea shells of the genus *Nassarius*, which appear, from the signs of polishing around the edges of the perforations, to have been attached in some way as explicitly decorative ornaments[^11^](#_ENREF_11). While apparently much earlier occurrences of symbolically incised red ochre – together with some other potentially “modern” behavioural features – have been reported from a number of MSA sites in the Pinnacle Point region (further east along the South African coast), with dates potentially reaching back to at least 100–150 ka[^8^](#_ENREF_8), the range and complexity of the (by any criteria) “modern” behavioural features recorded in the >75 ka levels in the Blombos cave remain, collectively, unparalleled in any in other comparably ancient sites in either Africa or other regions of the world (see below). The conceptual ability to source, combine, and store substances that enhance technology or social practices arguably represents a benchmark in the evolution of complex human cognition. Excavations in 2008 at Blombos Cave, South Africa, revealed a processing workshop where a liquefied ochre-rich mixture was produced and stored in two Haliotis midae (abalone) shells 100,000 years ago. Ochre, bone, charcoal, grindstones, and hammerstones form a composite part of this production toolkit. The application of the mixture is unknown, but possibilities include decoration and skin protection[^12^](#_ENREF_12).

On several grounds there are strong indications that southern Africa was the source of the earliest microlithic technology, reflected in the relatively sudden appearance of the so-called “Howiesions Poort” (HP) technologies across wide areas of southern Africa from ~70 ka onwards[^13^](#_ENREF_13)^,^ [^14^](#_ENREF_14)^,^ [^15^](#_ENREF_15). In stratified cave and rock-shelter sequences from at least ten sites, these technologies closely overlie the Still Bay industries, in which any trace of characteristic microlithic technology is conspicuously lacking. On the basis of the most recent dating evidence, the earliest occurrence of Howiesons Poort industries first appear ~68 ka, with the latest occurrences of the stratigraphically preceding Still Bay industries at ~72 ka, leaving a potential interval of at most 2–5 ka between these two sharply contrasting technologies[^13^](#_ENREF_13). The oldest known microliths, at Pinnacle Point, date to 70.6 ± 2.3 ka, preceding the Howiesons Poort industry by several thousand years and lasting in southern Africa for more than 10,000 years. The cultural and technological origins of the HP industries remains an essentially open question on archaeological grounds, but on stratigraphic and chronological grounds the most parsimonious explanation would be to see these industries in some way as a direct evolution from the closely preceding SB technologies, marked by a seemingly rapid invention of new microlithic technology, during the interval documented between the recorded ages of the two technologies discussed above. Exactly where and how this technological evolution occurred remains equally debatable. One possibility is that it occurred initially among closely coastally-adapted groups during a period when significantly lowered sea levels led to a consequent expansion in the exceptionally rich and productive coastal plain habitats around the South African coast, or at the end of this period when the waters readvanced[^14^](#_ENREF_14)^,^ [^16^](#_ENREF_16).

In any event, it is plausible that the transition from the SB, bifacially-flaked leaf-shaped point forms to the sharply contrasting microlith-dominated HP industries represented a significant increase in the economic (food-producing) capacities of the technologies involved, most probably related to the pursuit of either or both of terrestrial and, conceivably, marine mammals (such as seals, dolphins or whales). As discussed elsewhere[^7^](#_ENREF_7), potentially the most plausible explanation would be that the emergence of microlithic technology reflected the introduction of bow-and-arrow technology, for which the ability to kill or injure the targeted species at a much greater distance than that possible with the use of manually-propelled spear forms would have allowed a major increase in the economic, food producing adaptations of the human groups involved.

Several reseachers have argued for bow-and-arrow technology in the HP from analysis of a bone point and backed lithics in layers dating to ~64 ka at Sibudu (Backwell et al. 2008; Lombard and Phillipson 2010). Moreover, while microliths could potentially have served a number of other functions in the HP industries, another aspect of the archaeological record which provides strong empirical support for the archery interpretations is the remarkable similarity between the specific shapes of the microlithic forms encountered in the African HP industries[^17^](#_ENREF_17) and those which characterize the earliest stages of the Holocene (Mesolithic) period over large areas of the northwest European plain from ~11 ka onwards[^18^](#_ENREF_18)^,^ [^19^](#_ENREF_19)^.^ In both the HP and the north European sites, these include effectively identical forms of carefully shaped, “isosceles” triangular forms, together with equally distinctive trapezoidal and crescentic shapes, and simpler obliquely blunted point forms. In the north European sites, these types are widely interpreted as the tips and barbs of wooden arrows – as reflected best by the hafted example recovered from Lilla Loshult in southern Sweden, with the two microliths embedded in resin to a pinewood shaft to provide the tip and barb of the arrow, and with a clear notch at the proximal end of the shaft unambiguously reflecting its use as a bow-propelled armature[^20^](#_ENREF_20). While, needless to say, there is no suggestion of any direct cultural or technological connections across the vast spatial and chronological range, the close similarities in the detailed forms of the microliths in the African HP and European Mesolithic sites could be seen as a strong argument for analogous functions of the microlithic technologies in the South African HP industries. Further analysis of these tools for traces of use-wear or impact fractures could clearly strengthen the case for these interpretations.

While there is as yet no clear archaeological evidence for a direct technological evolution from the SB to the HP industries in southern Africa, the fact remains that no other plausible explanations for the origins of microlithic technology have so far been identified from either southern Africa or elsewhere in the Old World. Clearly, if the invention of archery technology conferred a strong, economically adaptive advantage on the archery equipped groups, this – combined with the broadly contemporaneous sharp decrease in aridity conditions within the central and eastern areas of Africa[^21^](#_ENREF_21) – could provide an obvious and highly plausible model for the demographic expansion of small human groups from southern into eastern Africa between ~70–60 ka, as suggested by the new mtDNA genetic evidence discussed above.

In addition to the emergence of microlithic technology, other features of the South African HP industries show a continuity and apparently increased complexity of the distinctively “modern” cultural features documented from the preceding SB industries (at Blombos, Pinnacle Point and elsewhere) in the form of carefully worked bone tools, as at Sibudu in north-eastern South Africa[^9^](#_ENREF_9). Especially significant are engraved ostrich eggshell fragments with symbolic designs recovered from the HP levels at the site of Diepkloof, close to the southwest African coast, and at the HP site Klipdrift Shelter, 40 km west of Blombos Cave on the southern Cape coast, where more than 100 pieces of engraved ostrich eggshell have been recovered[^15^](#_ENREF_15), which have been interpreted as potential symbols of personal ownership of ostrich eggshell water containers[^22^](#_ENREF_22). Both technologically and apparently symbolically, the HP industries would appear to reflect some significant increase in the distinctively “modern” features at the South African sites.

Currently, the best evidence for the appearance of typically HP industries within East Africa is provided by the two sites of the Mumba rock shelter (Tanzania) and an open-air site located on the Naisiusiu Beds at Olduvai Gorge – both showing an almost identical range of microlithic forms to those which characterize the South African sites, and with associated OSL dates of 56.9±4.8 and 49.1±4.3 ka for the Mumba industry[^23^](#_ENREF_23) and ESR dates of between 62.5±5 and 59±5 ka for the Naisiusiu site[^24^](#_ENREF_24) (although see[^25^](#_ENREF_25)). An important new development in symbolic form represented in the Mumba site is a number of carefully shaped, circular, perforated bead forms manufactured from ostrich eggshell fragments, and presumably representing personal decorative items[^26^](#_ENREF_26).

There is much less dating evidence so far for microliths in eastern Africa, with correspondingly less precision, and, as we mentioned in the main text, the 95% error ranges overlap substantially with the southern African ages. Therefore, any suggestion of an earlier appearance of microlithic technologies in southern Africa based on radiometric evidence alone (which essentially relies on the point estimates) needs to be treated with considerable cautious. Nevertheless, when combined with the evidence for the onset of more humid climatic conditions in East Africa ~70–60 ka[^21^](#_ENREF_21)^,^ [^27^](#_ENREF_27), it seems not unreasonable to entertain the possibility of a potential migration of HP populations from southern to eastern Africa at around this time – a hypothesis that can be further tested with genetic evidence, as we have done here.

The relatively abrupt disappearance of microlithic technologies in the later stages of the South African MSA industries (as at the inland site of Sibudu in north-eastern South Africa and Klasies River Mouth on the south-east South African coast[^9^](#_ENREF_9) and at Klipdrift Shelter where the HP is dated at 66–­59 ka[^15^](#_ENREF_15) could potentially be explained by a number of factors. Firstly, it should be recalled that functionally efficient arrows can be manufactured from purely organic materials such as sharply pointed wooden shafts, or alternatively with the tips and barbs of the arrows provided by minimally or entirely unretouched stone flakes, as documented in several recent hunter-gatherer communities, such as the South African San or the East African Hadza groups[^28^](#_ENREF_28), or with bone, which is attested in both the LSA and the HP[^29^](#_ENREF_29). Secondly, the northwards migration of human groups from southern to more central and eastern regions of Africa ~70–60 ka, as inferred from the most recent genetic data discussed above, could potentially have allowed the colonization of these areas by new human groups from other regions of Africa, still retaining more archaic forms of MSA technology.

In southern Africa, the seemingly abrupt reappearance of microlithic technologies in the earliest stage of the Later Stone Age period (from ~30 ka onwards[^30^](#_ENREF_30) could similarly be explained most economically and plausibly in one or both of two ways: either by a reversed demographic migration of human groups from the eastern to southern regions of Africa, conceivably stimulated by climatic and associated environmental changes (but for which there is no evident genetic signal), or potentially by increasing human population densities in the more northern regions; or alternatively by processes of cultural diffusion between adjacent human groups from eastern to southern Africa, stimulated by similar climatic changes or by other economic, social or other cultural mechanisms. But in any event, these early LSA industries in southern Africa are currently separated by an interval of at least 20–30 ka from the earlier HP technologies in the same regions, during which no technological continuity within South Africa itself has as yet been documented from the archaeological records[^30^](#_ENREF_30).

Finally, it is at present difficult to evaluate how much significance should be attached to the sporadic occurrence of much earlier “symbolic” features, reflected in the occasional specimens of perforated marine shells (of the same *Nassarius* genus as those at Blombos) recovered from sites dating back to at least ~100–130 ka in both North Africa and the adjacent Levantine coastal sites of Skhul and Qafzeh – accompanied at the latter site by evidence of clearly symbolic burial practices[^11^](#_ENREF_11). However, Powell et al[^31^](#_ENREF_31) have argued that these occurrences could potentially reflect early, but isolated, elements of cultural modernity which could have been “trumped” by demographic processes, in which these features were subsequently lost due to drift and population extinctions, during times of adverse climate that prevailed in tropical and southern Africa until ~ 90 ka, and in eastern Africa until ~80 ka[^21^](#_ENREF_21).

**Coalescent simulations**

To assess the statistical power of the proposed model, we performed coalescent simulations using the software ms [^32^](#_ENREF_32), in order to compare several alternative scenarios:

- - Scenario 1) Gene flow from the ancestral Khoe-San population to eastern Africa between 75 and 60 ka;
  - Scenario 2) Bidirectional gene flow between the ancestral Khoe-San population and eastern Africa between 75 and 60 ka;
  - Scenario 3) Gene flow from eastern Africa to the ancestral Khoe-San population between 75 and 60 ka;
  - Scenario 4) Gene flow from the ancestral Khoe-San population to eastern Africa between 125 and 75 ka;
  - Scenario 5) Bidirectional gene flow between the ancestral Khoe-San population and eastern Africa between 125 and 75 ka;
  - Scenario 6) Gene flow from eastern Africa to the ancestral Khoe-San population between 125 and 75 ka;
  - Scenario 7) Gene flow from the ancestral Khoe-San population to eastern Africa after 60 ka;
  - Scenario 8) Bidirectional gene flow between the ancestral Khoe-San population and eastern Africa after 60 ka;
  - Scenario 9) Gene flow from eastern Africa to the ancestral Khoe-San population after 60 ka;
  - Scenario 10) No discernible gene flow between the two populations following the initial population split (*i.e.,* independent evolution).

For each demographic model, we considered a sample size of 890 (on the ratio of 2.7 to 1 following the ratio between the Khoe-San dataset and the Luhya dataset from real data) and repeated the simulation 1000 times. For each demographic scenario we considered different sets of parameters (varying the duration of the migration, the proportion of migrants per generation in the target population and the migration rate per nucleotide per generation) resulting in a total of 244 models, as listed in table S5.

For each simulation, we computed the haplotype diversity, Tajima’s *D* and Fay and Wu's θ*_H_* [^33^](#_ENREF_33) in each population, and the difference between $\theta_{H}$ and the pairwise differences between sequences in both populations. The empirical likelihood (*P*) was then computed for each diversity measure following the procedure described in [^34^](#_ENREF_34). Let $x_{i}$ be the observed value for a given statistic and $\Omega_{i}$ the ordered set of corresponding statistics computed from the 1000 simulated datasets. Let us assume that $x_{i}$, greater than the median of $\Omega_{i}$, lies between the *k*^th^ and the (*k*+1)^th^. The empirical likelihood $P$ is obtained by counting the number of values between $x_{i}$ and 1000 and then doubling that figure to account for a two-tailed test: $P=2\times\frac{1000-k}{1000}$. To compute the overall *p-value*, we then used Fisher's method to combine statistics, as described by [^35^](#_ENREF_35)^,^ [^36^](#_ENREF_36). According to Fisher, $-2\sum_{i=1}^{k} \ln\left( P \right)\sim\chi_{2k}^{2}$. Here we considered three diversity measures from each population for a total of $k=6$ empirical likelihoods; thus we considered $\chi^{2}$ with 12 degrees of freedom. Since we performed multiple tests (244 models), we considered a Bonferroni correction in the significance level by assuming a significance level of $P=\frac{0.05}{244}\approx2.05\times{10}^{-4}$resulting in a $\chi^{2}$significance threshold of $37.2312$.

**Table S2.** Statistical evaluation of 10 alternative demographic scenarios. $\chi^{2}$ and *P-*values of non-significant models are highlighted.

| **Scenario** | **Migration duration** | **Migration rate** | **Mutation rate** | **Khoe-San** | | | **Eastern Africa** | | | **c^2^** | **p-Value** |
| --- | --- | --- | --- | --- | --- | --- | --- | --- | --- | --- | --- |
|  |  |  |  | **Segregating Sites** | **thetaH** | **H** | **Segregating Sites** | **thetaH** | H |  |  |
| **1** | 100 | 0.01 | 2.50E-08 | 0.0005 | 0.728 | 0.0005 | 0.0005 | 0.0005 | 0.214 | 64.5256867 | 3.34E-09 |
| **1** | 100 | 0.01 | 1.25E-07 | 0.168 | 0.004 | 0.556 | 0.0005 | 0.41 | 0.128 | ***36.8809296*** | ***0.00023356*** |
| **1** | 100 | 0.01 | 2.50E-07 | 0.002 | 0.0005 | 0.846 | 0.0005 | 0.012 | 0.084 | 56.9668721 | 8.01E-08 |
| **1** | 100 | 0.01 | 5.00E-07 | 0.0005 | 0.0005 | 0.674 | 0.0005 | 0.0005 | 0.084 | 66.550147 | 1.41E-09 |
| **1** | 100 | 0.1 | 2.50E-08 | 0.0005 | 0.762 | 0.0005 | 0.0005 | 0.0005 | 0.198 | 64.5898136 | 3.25E-09 |
| **1** | 100 | 0.1 | 1.25E-07 | 0.01 | 0.006 | 0.576 | 0.0005 | 0.398 | 0.114 | 41.9331524 | 3.42E-05 |
| **1** | 100 | 0.1 | 2.50E-07 | 0.002 | 0.0005 | 0.884 | 0.0005 | 0.01 | 0.098 | 56.9353384 | 8.12E-08 |
| **1** | 100 | 0.1 | 5.00E-07 | 0.0005 | 0.0005 | 0.64 | 0.0005 | 0.0005 | 0.114 | 66.0429075 | 1.75E-09 |
| **1** | 100 | 0.25 | 2.50E-08 | 0.0005 | 0.748 | 0.0005 | 0.0005 | 0.0005 | 0.218 | 64.4344447 | 3.47E-09 |
| **1** | 100 | 0.25 | 1.25E-07 | 0.006 | 0.01 | 0.534 | 0.0005 | 0.436 | 0.112 | 41.9375947 | 3.41E-05 |
| **1** | 100 | 0.25 | 2.50E-07 | 0.0005 | 0.0005 | 0.882 | 0.0005 | 0.014 | 0.108 | 58.8451852 | 3.66E-08 |
| **1** | 100 | 0.25 | 5.00E-07 | 0.0005 | 0.0005 | 0.68 | 0.0005 | 0.0005 | 0.104 | 66.1052734 | 1.70E-09 |
| **1** | 100 | 0.5 | 2.50E-08 | 0.0005 | 0.764 | 0.0005 | 0.0005 | 0.0005 | 0.212 | 64.4479327 | 3.45E-09 |
| **1** | 100 | 0.5 | 1.25E-07 | 0.004 | 0.0005 | 0.536 | 0.0005 | 0.402 | 0.082 | 49.5184524 | 1.70E-06 |
| **1** | 100 | 0.5 | 2.50E-07 | 0.004 | 0.0005 | 0.85 | 0.0005 | 0.012 | 0.096 | 55.304081 | 1.60E-07 |
| **1** | 100 | 0.5 | 5.00E-07 | 0.0005 | 0.0005 | 0.656 | 0.0005 | 0.0005 | 0.086 | 66.5572246 | 1.40E-09 |
| **1** | 1000 | 0.01 | 2.50E-08 | 0.0005 | 0.808 | 0.0005 | 0.0005 | 0.0005 | 0.208 | 64.3740405 | 3.56E-09 |
| **1** | 1000 | 0.01 | 1.25E-07 | 0.012 | 0.002 | 0.548 | 0.0005 | 0.382 | 0.126 | 43.7472944 | 1.69E-05 |
| **1** | 1000 | 0.01 | 2.50E-07 | 0.0005 | 0.0005 | 0.858 | 0.0005 | 0.016 | 0.08 | 59.2335075 | 3.11E-08 |
| **1** | 1000 | 0.01 | 5.00E-07 | 0.0005 | 0.0005 | 0.724 | 0.0005 | 0.0005 | 0.102 | 66.0187124 | 1.77E-09 |
| **1** | 1000 | 0.1 | 2.50E-08 | 0.0005 | 0.764 | 0.0005 | 0.0005 | 0.0005 | 0.228 | 64.302414 | 3.67E-09 |
| **1** | 1000 | 0.1 | 1.25E-07 | 0.006 | 0.0005 | 0.534 | 0.0005 | 0.386 | 0.124 | 47.9691036 | 3.16E-06 |
| **1** | 1000 | 0.1 | 2.50E-07 | 0.0005 | 0.0005 | 0.88 | 0.0005 | 0.022 | 0.108 | 57.9457553 | 5.33E-08 |
| **1** | 1000 | 0.1 | 5.00E-07 | 0.0005 | 0.0005 | 0.688 | 0.0005 | 0.0005 | 0.064 | 67.0528969 | 1.14E-09 |
| **1** | 1000 | 0.25 | 2.50E-08 | 0.0005 | 0.778 | 0.0005 | 0.0005 | 0.0005 | 0.232 | 64.231313 | 3.78E-09 |
| **1** | 1000 | 0.25 | 1.25E-07 | 0.008 | 0.006 | 0.58 | 0.0005 | 0.432 | 0.106 | 42.3471701 | 2.91E-05 |
| **1** | 1000 | 0.25 | 2.50E-07 | 0.002 | 0.0005 | 0.828 | 0.0005 | 0.02 | 0.1 | 55.6395265 | 1.39E-07 |
| **1** | 1000 | 0.25 | 5.00E-07 | 0.0005 | 0.0005 | 0.676 | 0.0005 | 0.0005 | 0.112 | 65.9688569 | 1.80E-09 |
| **1** | 1000 | 0.5 | 2.50E-08 | 0.0005 | 0.694 | 0.0005 | 0.0005 | 0.0005 | 0.158 | 65.2281068 | 2.47E-09 |
| **1** | 1000 | 0.5 | 1.25E-07 | 0.012 | 0.01 | 0.6 | 0.0005 | 0.39 | 0.116 | 40.4710411 | 6.00E-05 |
| **1** | 1000 | 0.5 | 2.50E-07 | 0.002 | 0.0005 | 0.822 | 0.0005 | 0.02 | 0.09 | 55.864793 | 1.27E-07 |
| **1** | 1000 | 0.5 | 5.00E-07 | 0.0005 | 0.0005 | 0.708 | 0.0005 | 0.0005 | 0.09 | 66.3137333 | 1.56E-09 |
| **1** | 5000 | 0.01 | 2.50E-08 | 0.0005 | 0.772 | 0.0005 | 0.0005 | 0.0005 | 0.198 | 64.5637376 | 3.28E-09 |
| **1** | 5000 | 0.01 | 1.25E-07 | 0.004 | 0.002 | 0.524 | 0.0005 | 0.376 | 0.124 | 46.0977498 | 6.67E-06 |
| **1** | 5000 | 0.01 | 2.50E-07 | 0.004 | 0.0005 | 0.874 | 0.0005 | 0.008 | 0.106 | 55.8611413 | 1.27E-07 |
| **1** | 5000 | 0.01 | 5.00E-07 | 0.0005 | 0.0005 | 0.7 | 0.0005 | 0.0005 | 0.102 | 66.0861345 | 1.72E-09 |
| **1** | 5000 | 0.1 | 2.50E-08 | 0.0005 | 0.75 | 0.0005 | 0.0005 | 0.0005 | 0.216 | 64.4475376 | 3.45E-09 |
| **1** | 5000 | 0.1 | 1.25E-07 | 0.014 | 0.002 | 0.53 | 0.0005 | 0.404 | 0.13 | 43.331296 | 1.98E-05 |
| **1** | 5000 | 0.1 | 2.50E-07 | 0.002 | 0.0005 | 0.846 | 0.0005 | 0.012 | 0.088 | 56.8738321 | 8.33E-08 |
| **1** | 5000 | 0.1 | 5.00E-07 | 0.0005 | 0.0005 | 0.704 | 0.0005 | 0.0005 | 0.114 | 65.8522872 | 1.90E-09 |
| **1** | 5000 | 0.25 | 2.50E-08 | 0.0005 | 0.78 | 0.0005 | 0.0005 | 0.002 | 0.228 | 61.488373 | 1.21E-08 |
| **1** | 5000 | 0.25 | 1.25E-07 | 0.004 | 0.01 | 0.532 | 0.0005 | 0.376 | 0.108 | 43.1248711 | 2.15E-05 |
| **1** | 5000 | 0.25 | 2.50E-07 | 0.004 | 0.0005 | 0.874 | 0.0005 | 0.01 | 0.096 | 55.613036 | 1.40E-07 |
| **1** | 5000 | 0.25 | 5.00E-07 | 0.0005 | 0.0005 | 0.664 | 0.0005 | 0.0005 | 0.104 | 66.1528947 | 1.67E-09 |
| **1** | 5000 | 0.5 | 2.50E-08 | 0.0005 | 0.76 | 0.0005 | 0.0005 | 0.0005 | 0.234 | 64.2609617 | 3.73E-09 |
| **1** | 5000 | 0.5 | 1.25E-07 | 0.004 | 0.002 | 0.568 | 0.0005 | 0.38 | 0.104 | 46.2671075 | 6.24E-06 |
| **1** | 5000 | 0.5 | 2.50E-07 | 0.0005 | 0.0005 | 0.884 | 0.0005 | 0.008 | 0.112 | 59.8871515 | 2.37E-08 |
| **1** | 5000 | 0.5 | 5.00E-07 | 0.0005 | 0.0005 | 0.698 | 0.0005 | 0.0005 | 0.092 | 66.2982254 | 1.57E-09 |
| **2** | 100 | 0.01 | 2.50E-08 | 0.0005 | 0.826 | 0.0005 | 0.0005 | 0.336 | 0.4 | 50.0016055 | 1.40E-06 |
| **2** | 100 | 0.01 | 1.25E-07 | 0.138 | 0.002 | 0.546 | 0.0005 | 0.392 | 0.334 | ***36.8685124*** | ***0.00023464*** |
| **2** | 100 | 0.01 | 2.50E-07 | 0.008 | 0.0005 | 0.87 | 0.0005 | 0.012 | 0.322 | 51.4508662 | 7.75E-07 |
| **2** | 100 | 0.01 | 5.00E-07 | 0.0005 | 0.0005 | 0.742 | 0.0005 | 0.0005 | 0.324 | 63.6580553 | 4.82E-09 |
| **2** | 100 | 0.1 | 2.50E-08 | 0.0005 | 0.786 | 0.0005 | 0.0005 | 0.396 | 0.42 | 49.674695 | 1.59E-06 |
| **2** | 100 | 0.1 | 1.25E-07 | 0.118 | 0.002 | 0.514 | 0.0005 | 0.422 | 0.368 | ***36.9610711*** | ***0.00022668*** |
| **2** | 100 | 0.1 | 2.50E-07 | 0.0005 | 0.0005 | 0.902 | 0.0005 | 0.016 | 0.328 | 56.3115127 | 1.05E-07 |
| **2** | 100 | 0.1 | 5.00E-07 | 0.0005 | 0.0005 | 0.708 | 0.0005 | 0.0005 | 0.352 | 63.5860903 | 4.97E-09 |
| **2** | 100 | 0.25 | 2.50E-08 | 0.0005 | 0.736 | 0.0005 | 0.0005 | 0.402 | 0.414 | 49.8048501 | 1.51E-06 |
| **2** | 100 | 0.25 | 1.25E-07 | 0.11 | 0.002 | 0.514 | 0.0005 | 0.414 | 0.356 | ***37.2060627*** | ***0.00020685*** |
| **2** | 100 | 0.25 | 2.50E-07 | 0.0005 | 0.0005 | 0.854 | 0.0005 | 0.012 | 0.37 | 56.7552647 | 8.75E-08 |
| **2** | 100 | 0.25 | 5.00E-07 | 0.0005 | 0.0005 | 0.752 | 0.0005 | 0.0005 | 0.374 | 63.3442565 | 5.51E-09 |
| **2** | 100 | 0.5 | 2.50E-08 | 0.0005 | 0.716 | 0.0005 | 0.0005 | 0.394 | 0.43 | 49.8243139 | 1.50E-06 |
| **2** | 100 | 0.5 | 1.25E-07 | 0.136 | 0.002 | 0.556 | 0.0005 | 0.446 | 0.294 | ***36.8584195*** | ***0.00023553*** |
| **2** | 100 | 0.5 | 2.50E-07 | 0.004 | 0.0005 | 0.89 | 0.0005 | 0.018 | 0.35 | 51.8140106 | 6.69E-07 |
| **2** | 100 | 0.5 | 5.00E-07 | 0.0005 | 0.0005 | 0.76 | 0.0005 | 0.0005 | 0.326 | 63.5978092 | 4.95E-09 |
| **2** | 1000 | 0.01 | 2.50E-08 | 0.0005 | 0.804 | 0.0005 | 0.0005 | 0.37 | 0.416 | 49.7843714 | 1.52E-06 |
| **2** | 1000 | 0.01 | 1.25E-07 | 0.142 | 0.004 | 0.522 | 0.0005 | 0.414 | 0.356 | ***35.2781863*** | ***0.00042284*** |
| **2** | 1000 | 0.01 | 2.50E-07 | 0.0005 | 0.0005 | 0.93 | 0.0005 | 0.014 | 0.342 | 56.4338411 | 1.00E-07 |
| **2** | 1000 | 0.01 | 5.00E-07 | 0.0005 | 0.0005 | 0.716 | 0.0005 | 0.0005 | 0.336 | 63.6566581 | 4.82E-09 |
| **2** | 1000 | 0.1 | 2.50E-08 | 0.0005 | 0.808 | 0.0005 | 0.0005 | 0.392 | 0.442 | 49.5376789 | 1.68E-06 |
| **2** | 1000 | 0.1 | 1.25E-07 | 0.136 | 0.002 | 0.608 | 0.0005 | 0.444 | 0.362 | ***36.2724663*** | ***0.00029288*** |
| **2** | 1000 | 0.1 | 2.50E-07 | 0.0005 | 0.0005 | 0.86 | 0.0005 | 0.01 | 0.356 | 57.18305 | 7.32E-08 |
| **2** | 1000 | 0.1 | 5.00E-07 | 0.0005 | 0.0005 | 0.672 | 0.0005 | 0.0005 | 0.344 | 63.7364408 | 4.66E-09 |
| **2** | 1000 | 0.25 | 2.50E-08 | 0.0005 | 0.76 | 0.0005 | 0.0005 | 0.374 | 0.398 | 49.963894 | 1.42E-06 |
| **2** | 1000 | 0.25 | 1.25E-07 | 0.128 | 0.002 | 0.568 | 0.0005 | 0.41 | 0.348 | ***36.7680407*** | ***0.00024359*** |
| **2** | 1000 | 0.25 | 2.50E-07 | 0.002 | 0.0005 | 0.932 | 0.0005 | 0.016 | 0.342 | 53.3898932 | 3.51E-07 |
| **2** | 1000 | 0.25 | 5.00E-07 | 0.0005 | 0.0005 | 0.696 | 0.0005 | 0.0005 | 0.35 | 63.6316752 | 4.88E-09 |
| **2** | 1000 | 0.5 | 2.50E-08 | 0.0005 | 0.804 | 0.0005 | 0.0005 | 0.392 | 0.44 | 49.5566748 | 1.67E-06 |
| **2** | 1000 | 0.5 | 1.25E-07 | 0.104 | 0.006 | 0.572 | 0.0005 | 0.402 | 0.402 | ***34.7229706*** | ***0.00051831*** |
| **2** | 1000 | 0.5 | 2.50E-07 | 0.0005 | 0.0005 | 0.922 | 0.0005 | 0.016 | 0.336 | 56.2194562 | 1.09E-07 |
| **2** | 1000 | 0.5 | 5.00E-07 | 0.0005 | 0.0005 | 0.694 | 0.0005 | 0.0005 | 0.32 | 63.8166549 | 4.51E-09 |
| **2** | 5000 | 0.01 | 2.50E-08 | 0.0005 | 0.768 | 0.0005 | 0.0005 | 0.282 | 0.356 | 50.7306914 | 1.04E-06 |
| **2** | 5000 | 0.01 | 1.25E-07 | 0.098 | 0.01 | 0.506 | 0.0005 | 0.44 | 0.294 | ***34.5104702*** | ***0.00056015*** |
| **2** | 5000 | 0.01 | 2.50E-07 | 0.006 | 0.0005 | 0.852 | 0.0005 | 0.016 | 0.34 | 51.3838914 | 7.97E-07 |
| **2** | 5000 | 0.01 | 5.00E-07 | 0.0005 | 0.0005 | 0.67 | 0.0005 | 0.0005 | 0.326 | 63.8498906 | 4.44E-09 |
| **2** | 5000 | 0.1 | 2.50E-08 | 0.0005 | 0.732 | 0.0005 | 0.0005 | 0.334 | 0.41 | 50.2057891 | 1.29E-06 |
| **2** | 5000 | 0.1 | 1.25E-07 | 0.152 | 0.006 | 0.6 | 0.0005 | 0.378 | 0.296 | ***34.6037111*** | ***0.0005414*** |
| **2** | 5000 | 0.1 | 2.50E-07 | 0.004 | 0.0005 | 0.84 | 0.0005 | 0.016 | 0.292 | 52.5275745 | 5.00E-07 |
| **2** | 5000 | 0.1 | 5.00E-07 | 0.0005 | 0.0005 | 0.678 | 0.0005 | 0.0005 | 0.302 | 63.9790922 | 4.21E-09 |
| **2** | 5000 | 0.25 | 2.50E-08 | 0.0005 | 0.752 | 0.0005 | 0.0005 | 0.356 | 0.396 | 50.0937839 | 1.35E-06 |
| **2** | 5000 | 0.25 | 1.25E-07 | 0.11 | 0.004 | 0.536 | 0.0005 | 0.416 | 0.304 | ***36.042114*** | ***0.00031898*** |
| **2** | 5000 | 0.25 | 2.50E-07 | 0.0005 | 0.0005 | 0.846 | 0.0005 | 0.018 | 0.306 | 56.342994 | 1.04E-07 |
| **2** | 5000 | 0.25 | 5.00E-07 | 0.0005 | 0.0005 | 0.718 | 0.0005 | 0.0005 | 0.348 | 63.5808967 | 4.98E-09 |
| **2** | 5000 | 0.5 | 2.50E-08 | 0.0005 | 0.778 | 0.0005 | 0.0005 | 0.326 | 0.378 | 50.2949102 | 1.24E-06 |
| **2** | 5000 | 0.5 | 1.25E-07 | 0.148 | 0.002 | 0.542 | 0.0005 | 0.464 | 0.388 | ***36.106327*** | ***0.00031149*** |
| **2** | 5000 | 0.5 | 2.50E-07 | 0.004 | 0.0005 | 0.894 | 0.0005 | 0.014 | 0.308 | 52.5633376 | 4.93E-07 |
| **2** | 5000 | 0.5 | 5.00E-07 | 0.0005 | 0.0005 | 0.704 | 0.0005 | 0.0005 | 0.318 | 63.8005813 | 4.54E-09 |
| **3** | 100 | 0.01 | 2.50E-08 | 0.0005 | 0.768 | 0.0005 | 0.0005 | 0.402 | 0.46 | 49.5090098 | 1.70E-06 |
| **3** | 100 | 0.01 | 1.25E-07 | 0.016 | 0.012 | 0.554 | 0.0005 | 0.41 | 0.368 | 37.2815574 | 0.00020109 |
| **3** | 100 | 0.01 | 2.50E-07 | 0.002 | 0.0005 | 0.844 | 0.0005 | 0.012 | 0.288 | 54.5073185 | 2.22E-07 |
| **3** | 100 | 0.01 | 5.00E-07 | 0.0005 | 0.0005 | 0.67 | 0.0005 | 0.0005 | 0.328 | 63.8376582 | 4.47E-09 |
| **3** | 100 | 0.1 | 2.50E-08 | 0.0005 | 0.774 | 0.0005 | 0.0005 | 0.002 | 0.226 | 61.5214383 | 1.19E-08 |
| **3** | 100 | 0.1 | 1.25E-07 | 0.024 | 0.01 | 0.566 | 0.0005 | 0.476 | 0.122 | 38.7020139 | 0.00011781 |
| **3** | 100 | 0.1 | 2.50E-07 | 0.004 | 0.002 | 0.886 | 0.0005 | 0.006 | 0.13 | 53.2284529 | 3.75E-07 |
| **3** | 100 | 0.1 | 5.00E-07 | 0.0005 | 0.0005 | 0.72 | 0.0005 | 0.0005 | 0.134 | 65.4840588 | 2.22E-09 |
| **3** | 100 | 0.25 | 2.50E-08 | 0.0005 | 0.748 | 0.0005 | 0.0005 | 0.004 | 0.208 | 60.3694756 | 1.93E-08 |
| **3** | 100 | 0.25 | 1.25E-07 | 0.018 | 0.004 | 0.54 | 0.0005 | 0.41 | 0.146 | 41.1433596 | 4.64E-05 |
| **3** | 100 | 0.25 | 2.50E-07 | 0.002 | 0.0005 | 0.866 | 0.0005 | 0.018 | 0.112 | 55.5338466 | 1.45E-07 |
| **3** | 100 | 0.25 | 5.00E-07 | 0.0005 | 0.0005 | 0.688 | 0.0005 | 0.0005 | 0.148 | 65.3762386 | 2.32E-09 |
| **3** | 100 | 0.5 | 2.50E-08 | 0.0005 | 0.806 | 0.0005 | 0.0005 | 0.004 | 0.236 | 59.9675266 | 2.29E-08 |
| **3** | 100 | 0.5 | 1.25E-07 | 0.014 | 0.002 | 0.58 | 0.0005 | 0.44 | 0.152 | 42.667582 | 2.57E-05 |
| **3** | 100 | 0.5 | 2.50E-07 | 0.002 | 0.0005 | 0.902 | 0.0005 | 0.012 | 0.112 | 56.2633176 | 1.07E-07 |
| **3** | 100 | 0.5 | 5.00E-07 | 0.0005 | 0.0005 | 0.722 | 0.0005 | 0.0005 | 0.124 | 65.6336274 | 2.08E-09 |
| **3** | 1000 | 0.01 | 2.50E-08 | 0.0005 | 0.712 | 0.0005 | 0.0005 | 0.002 | 0.234 | 61.618854 | 1.14E-08 |
| **3** | 1000 | 0.01 | 1.25E-07 | 0.016 | 0.0005 | 0.512 | 0.0005 | 0.404 | 0.158 | 45.5158056 | 8.41E-06 |
| **3** | 1000 | 0.01 | 2.50E-07 | 0.004 | 0.0005 | 0.796 | 0.0005 | 0.006 | 0.128 | 56.2462855 | 1.08E-07 |
| **3** | 1000 | 0.01 | 5.00E-07 | 0.0005 | 0.0005 | 0.664 | 0.0005 | 0.0005 | 0.128 | 65.737616 | 1.99E-09 |
| **3** | 1000 | 0.1 | 2.50E-08 | 0.0005 | 0.746 | 0.0005 | 0.0005 | 0.006 | 0.228 | 59.380285 | 2.93E-08 |
| **3** | 1000 | 0.1 | 1.25E-07 | 0.014 | 0.004 | 0.57 | 0.0005 | 0.4 | 0.152 | 41.5066915 | 4.03E-05 |
| **3** | 1000 | 0.1 | 2.50E-07 | 0.002 | 0.0005 | 0.87 | 0.0005 | 0.012 | 0.164 | 55.5728251 | 1.43E-07 |
| **3** | 1000 | 0.1 | 5.00E-07 | 0.0005 | 0.0005 | 0.694 | 0.0005 | 0.0005 | 0.116 | 65.8461165 | 1.90E-09 |
| **3** | 1000 | 0.25 | 2.50E-08 | 0.0005 | 0.764 | 0.0005 | 0.0005 | 0.012 | 0.216 | 58.0544407 | 5.10E-08 |
| **3** | 1000 | 0.25 | 1.25E-07 | 0.012 | 0.002 | 0.53 | 0.0005 | 0.396 | 0.148 | 43.4202431 | 1.92E-05 |
| **3** | 1000 | 0.25 | 2.50E-07 | 0.0005 | 0.0005 | 0.892 | 0.0005 | 0.018 | 0.146 | 57.7170574 | 5.86E-08 |
| **3** | 1000 | 0.25 | 5.00E-07 | 0.0005 | 0.0005 | 0.65 | 0.0005 | 0.0005 | 0.116 | 65.9771157 | 1.80E-09 |
| **3** | 1000 | 0.5 | 2.50E-08 | 0.0005 | 0.75 | 0.0005 | 0.0005 | 0.004 | 0.238 | 60.0946699 | 2.17E-08 |
| **3** | 1000 | 0.5 | 1.25E-07 | 0.022 | 0.012 | 0.55 | 0.0005 | 0.41 | 0.138 | 38.6208013 | 0.00012149 |
| **3** | 1000 | 0.5 | 2.50E-07 | 0.002 | 0.0005 | 0.882 | 0.0005 | 0.008 | 0.14 | 56.6728057 | 9.06E-08 |
| **3** | 1000 | 0.5 | 5.00E-07 | 0.0005 | 0.0005 | 0.64 | 0.0005 | 0.0005 | 0.142 | 65.6036503 | 2.11E-09 |
| **3** | 5000 | 0.01 | 2.50E-08 | 0.0005 | 0.758 | 0.0005 | 0.0005 | 0.006 | 0.206 | 59.5513084 | 2.72E-08 |
| **3** | 5000 | 0.01 | 1.25E-07 | 0.014 | 0.01 | 0.534 | 0.0005 | 0.366 | 0.114 | 40.5576176 | 5.81E-05 |
| **3** | 5000 | 0.01 | 2.50E-07 | 0.006 | 0.0005 | 0.902 | 0.0005 | 0.022 | 0.136 | 52.4655094 | 5.13E-07 |
| **3** | 5000 | 0.01 | 5.00E-07 | 0.0005 | 0.0005 | 0.678 | 0.0005 | 0.0005 | 0.136 | 65.5746364 | 2.13E-09 |
| **3** | 5000 | 0.1 | 2.50E-08 | 0.0005 | 0.836 | 0.0005 | 0.0005 | 0.002 | 0.216 | 61.457838 | 1.22E-08 |
| **3** | 5000 | 0.1 | 1.25E-07 | 0.008 | 0.0005 | 0.558 | 0.0005 | 0.36 | 0.144 | 47.1462164 | 4.40E-06 |
| **3** | 5000 | 0.1 | 2.50E-07 | 0.004 | 0.0005 | 0.892 | 0.0005 | 0.01 | 0.126 | 55.0283971 | 1.79E-07 |
| **3** | 5000 | 0.1 | 5.00E-07 | 0.0005 | 0.0005 | 0.69 | 0.0005 | 0.0005 | 0.132 | 65.5992538 | 2.11E-09 |
| **3** | 5000 | 0.25 | 2.50E-08 | 0.0005 | 0.812 | 0.0005 | 0.0005 | 0.002 | 0.218 | 61.4976613 | 1.20E-08 |
| **3** | 5000 | 0.25 | 1.25E-07 | 0.026 | 0.002 | 0.538 | 0.0005 | 0.398 | 0.146 | 41.8610359 | 3.51E-05 |
| **3** | 5000 | 0.25 | 2.50E-07 | 0.004 | 0.0005 | 0.92 | 0.0005 | 0.022 | 0.11 | 53.6612704 | 3.14E-07 |
| **3** | 5000 | 0.25 | 5.00E-07 | 0.0005 | 0.0005 | 0.638 | 0.0005 | 0.0005 | 0.128 | 65.8175037 | 1.92E-09 |
| **3** | 5000 | 0.5 | 2.50E-08 | 0.0005 | 0.762 | 0.0005 | 0.0005 | 0.002 | 0.252 | 61.3349008 | 1.29E-08 |
| **3** | 5000 | 0.5 | 1.25E-07 | 0.004 | 0.0005 | 0.602 | 0.0005 | 0.414 | 0.13 | 48.3057476 | 2.76E-06 |
| **3** | 5000 | 0.5 | 2.50E-07 | 0.002 | 0.0005 | 0.864 | 0.0005 | 0.016 | 0.118 | 55.6696655 | 1.37E-07 |
| **3** | 5000 | 0.5 | 5.00E-07 | 0.0005 | 0.0005 | 0.678 | 0.0005 | 0.0005 | 0.124 | 65.7593831 | 1.97E-09 |
| **4** | 45000 | 0.01 | 2.50E-08 | 0.0005 | 0.754 | 0.0005 | 0.0005 | 0.0005 | 0.226 | 64.3463861 | 3.60E-09 |
| **4** | 45000 | 0.01 | 1.25E-07 | 0.006 | 0.006 | 0.6 | 0.0005 | 0.39 | 0.116 | 42.8789867 | 2.37E-05 |
| **4** | 45000 | 0.01 | 2.50E-07 | 0.004 | 0.0005 | 0.866 | 0.0005 | 0.014 | 0.122 | 54.4791368 | 2.24E-07 |
| **4** | 45000 | 0.01 | 5.00E-07 | 0.0005 | 0.0005 | 0.678 | 0.0005 | 0.0005 | 0.106 | 66.073068 | 1.73E-09 |
| **4** | 45000 | 0.1 | 2.50E-08 | 0.0005 | 0.738 | 0.0005 | 0.0005 | 0.0005 | 0.218 | 64.461363 | 3.43E-09 |
| **4** | 45000 | 0.1 | 1.25E-07 | 0.016 | 0.004 | 0.56 | 0.0005 | 0.45 | 0.108 | 41.7229604 | 3.71E-05 |
| **4** | 45000 | 0.1 | 2.50E-07 | 0.006 | 0.0005 | 0.822 | 0.0005 | 0.014 | 0.126 | 53.7079739 | 3.08E-07 |
| **4** | 45000 | 0.1 | 5.00E-07 | 0.0005 | 0.0005 | 0.722 | 0.0005 | 0.0005 | 0.128 | 65.57013 | 2.14E-09 |
| **4** | 45000 | 0.25 | 2.50E-08 | 0.0005 | 0.74 | 0.0005 | 0.0005 | 0.0005 | 0.194 | 64.6892241 | 3.11E-09 |
| **4** | 45000 | 0.25 | 1.25E-07 | 0.018 | 0.008 | 0.53 | 0.0005 | 0.428 | 0.1 | 40.4653903 | 6.02E-05 |
| **4** | 45000 | 0.25 | 2.50E-07 | 0.0005 | 0.0005 | 0.864 | 0.0005 | 0.02 | 0.1 | 58.326996 | 4.55E-08 |
| **4** | 45000 | 0.25 | 5.00E-07 | 0.0005 | 0.0005 | 0.704 | 0.0005 | 0.0005 | 0.112 | 65.8876863 | 1.87E-09 |
| **4** | 45000 | 0.5 | 2.50E-08 | 0.0005 | 0.752 | 0.0005 | 0.0005 | 0.002 | 0.222 | 61.6148247 | 1.14E-08 |
| **4** | 45000 | 0.5 | 1.25E-07 | 0.008 | 0.004 | 0.554 | 0.0005 | 0.428 | 0.106 | 43.268432 | 2.03E-05 |
| **4** | 45000 | 0.5 | 2.50E-07 | 0.002 | 0.0005 | 0.828 | 0.0005 | 0.016 | 0.106 | 55.9692758 | 1.21E-07 |
| **4** | 45000 | 0.5 | 5.00E-07 | 0.0005 | 0.0005 | 0.714 | 0.0005 | 0.0005 | 0.092 | 66.2528977 | 1.60E-09 |
| **5** | 45000 | 0.01 | 2.50E-08 | 0.0005 | 0.738 | 0.0005 | 0.0005 | 0.006 | 0.218 | 59.4915497 | 2.79E-08 |
| **5** | 45000 | 0.01 | 1.25E-07 | 0.018 | 0.014 | 0.562 | 0.0005 | 0.408 | 0.102 | 39.2850159 | 9.44E-05 |
| **5** | 45000 | 0.01 | 2.50E-07 | 0.004 | 0.0005 | 0.924 | 0.0005 | 0.01 | 0.09 | 55.6308497 | 1.39E-07 |
| **5** | 45000 | 0.01 | 5.00E-07 | 0.0005 | 0.0005 | 0.684 | 0.0005 | 0.0005 | 0.118 | 65.8409557 | 1.91E-09 |
| **5** | 45000 | 0.1 | 2.50E-08 | 0.0005 | 0.75 | 0.0005 | 0.0005 | 0.002 | 0.2 | 61.8288709 | 1.05E-08 |
| **5** | 45000 | 0.1 | 1.25E-07 | 0.034 | 0.006 | 0.536 | 0.0005 | 0.434 | 0.16 | 38.7784127 | 0.00011445 |
| **5** | 45000 | 0.1 | 2.50E-07 | 0.0005 | 0.0005 | 0.824 | 0.0005 | 0.018 | 0.108 | 58.4785994 | 4.27E-08 |
| **5** | 45000 | 0.1 | 5.00E-07 | 0.0005 | 0.0005 | 0.678 | 0.0005 | 0.0005 | 0.128 | 65.6958857 | 2.03E-09 |
| **5** | 45000 | 0.25 | 2.50E-08 | 0.0005 | 0.778 | 0.0005 | 0.0005 | 0.004 | 0.256 | 59.8755498 | 2.38E-08 |
| **5** | 45000 | 0.25 | 1.25E-07 | 0.016 | 0.006 | 0.57 | 0.0005 | 0.44 | 0.156 | 40.1861271 | 6.70E-05 |
| **5** | 45000 | 0.25 | 2.50E-07 | 0.0005 | 0.0005 | 0.844 | 0.0005 | 0.008 | 0.1 | 60.206418 | 2.07E-08 |
| **5** | 45000 | 0.25 | 5.00E-07 | 0.0005 | 0.0005 | 0.678 | 0.0005 | 0.0005 | 0.086 | 66.4912516 | 1.44E-09 |
| **5** | 45000 | 0.5 | 2.50E-08 | 0.0005 | 0.704 | 0.0005 | 0.0005 | 0.006 | 0.214 | 59.6229188 | 2.64E-08 |
| **5** | 45000 | 0.5 | 1.25E-07 | 0.014 | 0.004 | 0.556 | 0.0005 | 0.384 | 0.156 | 41.5861206 | 3.91E-05 |
| **5** | 45000 | 0.5 | 2.50E-07 | 0.0005 | 0.0005 | 0.864 | 0.0005 | 0.016 | 0.122 | 58.3755814 | 4.46E-08 |
| **5** | 45000 | 0.5 | 5.00E-07 | 0.0005 | 0.0005 | 0.708 | 0.0005 | 0.0005 | 0.102 | 66.063407 | 1.73E-09 |
| **6** | 45000 | 0.01 | 2.50E-08 | 0.0005 | 0.748 | 0.0005 | 0.0005 | 0.0005 | 0.196 | 64.6472055 | 3.17E-09 |
| **6** | 45000 | 0.01 | 1.25E-07 | 0.02 | 0.004 | 0.526 | 0.0005 | 0.42 | 0.13 | 41.1691237 | 4.59E-05 |
| **6** | 45000 | 0.01 | 2.50E-07 | 0.0005 | 0.0005 | 0.884 | 0.0005 | 0.01 | 0.126 | 59.2052983 | 3.15E-08 |
| **6** | 45000 | 0.01 | 5.00E-07 | 0.0005 | 0.0005 | 0.7 | 0.0005 | 0.0005 | 0.12 | 65.7610966 | 1.97E-09 |
| **6** | 45000 | 0.1 | 2.50E-08 | 0.0005 | 0.778 | 0.0005 | 0.0005 | 0.004 | 0.194 | 60.4301883 | 1.88E-08 |
| **6** | 45000 | 0.1 | 1.25E-07 | 0.022 | 0.004 | 0.596 | 0.0005 | 0.428 | 0.126 | 40.7533925 | 5.39E-05 |
| **6** | 45000 | 0.1 | 2.50E-07 | 0.0005 | 0.0005 | 0.8 | 0.0005 | 0.014 | 0.1 | 59.1942679 | 3.16E-08 |
| **6** | 45000 | 0.1 | 5.00E-07 | 0.0005 | 0.0005 | 0.71 | 0.0005 | 0.0005 | 0.098 | 66.1377759 | 1.68E-09 |
| **6** | 45000 | 0.25 | 2.50E-08 | 0.0005 | 0.776 | 0.0005 | 0.0005 | 0.006 | 0.188 | 59.6872385 | 2.57E-08 |
| **6** | 45000 | 0.25 | 1.25E-07 | 0.01 | 0.004 | 0.53 | 0.0005 | 0.412 | 0.086 | 43.4051035 | 1.93E-05 |
| **6** | 45000 | 0.25 | 2.50E-07 | 0.002 | 0.0005 | 0.862 | 0.0005 | 0.016 | 0.118 | 55.6743005 | 1.37E-07 |
| **6** | 45000 | 0.25 | 5.00E-07 | 0.0005 | 0.0005 | 0.64 | 0.0005 | 0.0005 | 0.084 | 66.6536708 | 1.35E-09 |
| **6** | 45000 | 0.5 | 2.50E-08 | 0.0005 | 0.84 | 0.0005 | 0.0005 | 0.002 | 0.19 | 61.7048001 | 1.10E-08 |
| **6** | 45000 | 0.5 | 1.25E-07 | 0.02 | 0.002 | 0.566 | 0.0005 | 0.398 | 0.116 | 42.7443263 | 2.49E-05 |
| **6** | 45000 | 0.5 | 2.50E-07 | 0.002 | 0.0005 | 0.882 | 0.0005 | 0.008 | 0.138 | 56.7015831 | 8.95E-08 |
| **6** | 45000 | 0.5 | 5.00E-07 | 0.0005 | 0.0005 | 0.652 | 0.0005 | 0.0005 | 0.09 | 66.4785323 | 1.45E-09 |
| **7** | 50000 | 0.01 | 2.50E-08 | 0.0005 | 0.202 | 0.0005 | 0.0005 | 0.002 | 0.238 | 64.1045753 | 3.99E-09 |
| **7** | 50000 | 0.01 | 1.25E-07 | 0.248 | 0.0005 | 0.96 | 0.0005 | 0.486 | 0.13 | 38.7974419 | 0.00011363 |
| **7** | 50000 | 0.01 | 2.50E-07 | 0.002 | 0.0005 | 0.78 | 0.0005 | 0.024 | 0.108 | 55.2403998 | 1.64E-07 |
| **7** | 50000 | 0.01 | 5.00E-07 | 0.0005 | 0.0005 | 0.676 | 0.0005 | 0.0005 | 0.102 | 66.155909 | 1.67E-09 |
| **7** | 50000 | 0.1 | 2.50E-08 | 0.0005 | 0.186 | 0.0005 | 0.0005 | 0.0005 | 0.222 | 67.1813927 | 1.07E-09 |
| **7** | 50000 | 0.1 | 1.25E-07 | 0.218 | 0.0005 | 0.956 | 0.0005 | 0.386 | 0.128 | 39.5554109 | 8.52E-05 |
| **7** | 50000 | 0.1 | 2.50E-07 | 0.0005 | 0.0005 | 0.702 | 0.0005 | 0.01 | 0.096 | 60.2102131 | 2.07E-08 |
| **7** | 50000 | 0.1 | 5.00E-07 | 0.0005 | 0.0005 | 0.586 | 0.0005 | 0.0005 | 0.106 | 66.364723 | 1.52E-09 |
| **7** | 50000 | 0.25 | 2.50E-08 | 0.0005 | 0.204 | 0.0005 | 0.0005 | 0.0005 | 0.198 | 67.2254667 | 1.05E-09 |
| **7** | 50000 | 0.25 | 1.25E-07 | 0.23 | 0.002 | 0.982 | 0.0005 | 0.404 | 0.13 | ***36.4998235*** | ***0.00026917*** |
| **7** | 50000 | 0.25 | 2.50E-07 | 0.0005 | 0.0005 | 0.75 | 0.0005 | 0.01 | 0.112 | 59.7696321 | 2.49E-08 |
| **7** | 50000 | 0.25 | 5.00E-07 | 0.0005 | 0.0005 | 0.656 | 0.0005 | 0.0005 | 0.104 | 66.1771374 | 1.65E-09 |
| **7** | 50000 | 0.5 | 2.50E-08 | 0.0005 | 0.258 | 0.0005 | 0.0005 | 0.0005 | 0.198 | 66.7557876 | 1.29E-09 |
| **7** | 50000 | 0.5 | 1.25E-07 | 0.242 | 0.0005 | 0.97 | 0.0005 | 0.392 | 0.114 | 39.5182639 | 8.64E-05 |
| **7** | 50000 | 0.5 | 2.50E-07 | 0.0005 | 0.0005 | 0.694 | 0.0005 | 0.012 | 0.102 | 59.7472436 | 2.51E-08 |
| **7** | 50000 | 0.5 | 5.00E-07 | 0.0005 | 0.0005 | 0.614 | 0.0005 | 0.0005 | 0.128 | 65.8941904 | 1.86E-09 |
| **8** | 50000 | 0.01 | 2.50E-08 | 0.0005 | 0.16 | 0.0005 | 0.0005 | 0.658 | 0.632 | 51.0254101 | 9.22E-07 |
| **8** | 50000 | 0.01 | 1.25E-07 | 0.852 | 0.0005 | 0.728 | 0.0005 | 0.182 | 0.526 | ***36.0512611*** | ***0.0003179*** |
| **8** | 50000 | 0.01 | 2.50E-07 | 0.0005 | 0.0005 | 0.982 | 0.0005 | 0.004 | 0.506 | 58.0471018 | 5.11E-08 |
| **8** | 50000 | 0.01 | 5.00E-07 | 0.0005 | 0.0005 | 0.942 | 0.0005 | 0.0005 | 0.518 | 62.2422798 | 8.78E-09 |
| **8** | 50000 | 0.1 | 2.50E-08 | 0.0005 | 0.166 | 0.0005 | 0.0005 | 0.622 | 0.59 | 51.2018456 | 8.58E-07 |
| **8** | 50000 | 0.1 | 1.25E-07 | 0.848 | 0.0005 | 0.774 | 0.0005 | 0.186 | 0.528 | ***35.8870611*** | ***0.00033782*** |
| **8** | 50000 | 0.1 | 2.50E-07 | 0.0005 | 0.0005 | 0.908 | 0.0005 | 0.008 | 0.484 | 56.9064048 | 8.22E-08 |
| **8** | 50000 | 0.1 | 5.00E-07 | 0.0005 | 0.0005 | 0.86 | 0.0005 | 0.0005 | 0.526 | 62.3937736 | 8.24E-09 |
| **8** | 50000 | 0.25 | 2.50E-08 | 0.0005 | 0.118 | 0.0005 | 0.0005 | 0.668 | 0.616 | 51.6555069 | 7.13E-07 |
| **8** | 50000 | 0.25 | 1.25E-07 | 0.86 | 0.0005 | 0.74 | 0.0005 | 0.174 | 0.526 | ***36.0897739*** | ***0.0003134*** |
| **8** | 50000 | 0.25 | 2.50E-07 | 0.002 | 0.0005 | 0.936 | 0.0005 | 0.01 | 0.514 | 53.50651 | 3.35E-07 |
| **8** | 50000 | 0.25 | 5.00E-07 | 0.0005 | 0.0005 | 0.91 | 0.0005 | 0.0005 | 0.51 | 62.3425301 | 8.42E-09 |
| **8** | 50000 | 0.5 | 2.50E-08 | 0.0005 | 0.17 | 0.0005 | 0.0005 | 0.642 | 0.626 | 50.9724722 | 9.42E-07 |
| **8** | 50000 | 0.5 | 1.25E-07 | 0.88 | 0.004 | 0.75 | 0.0005 | 0.176 | 0.554 | ***31.7314814*** | ***0.00152209*** |
| **8** | 50000 | 0.5 | 2.50E-07 | 0.004 | 0.0005 | 0.988 | 0.0005 | 0.002 | 0.454 | 55.4792092 | 1.48E-07 |
| **8** | 50000 | 0.5 | 5.00E-07 | 0.0005 | 0.0005 | 0.884 | 0.0005 | 0.0005 | 0.472 | 62.5553687 | 7.69E-09 |
| **9** | 50000 | 0.01 | 2.50E-08 | 0.0005 | 0.782 | 0.0005 | 0.0005 | 0.01 | 0.498 | 56.7018666 | 8.95E-08 |
| **9** | 50000 | 0.01 | 1.25E-07 | 0.016 | 0.0005 | 0.612 | 0.0005 | 0.898 | 0.294 | 42.3195104 | 2.94E-05 |
| **9** | 50000 | 0.01 | 2.50E-07 | 0.0005 | 0.0005 | 0.882 | 0.0005 | 0.226 | 0.282 | 51.3626782 | 8.04E-07 |
| **9** | 50000 | 0.01 | 5.00E-07 | 0.0005 | 0.0005 | 0.68 | 0.0005 | 0.012 | 0.3 | 57.6303826 | 6.08E-08 |
| **9** | 50000 | 0.1 | 2.50E-08 | 0.0005 | 0.72 | 0.0005 | 0.0005 | 0.006 | 0.474 | 57.9875104 | 5.24E-08 |
| **9** | 50000 | 0.1 | 1.25E-07 | 0.022 | 0.004 | 0.54 | 0.0005 | 0.89 | 0.314 | 37.6603169 | 0.00017448 |
| **9** | 50000 | 0.1 | 2.50E-07 | 0.002 | 0.0005 | 0.858 | 0.0005 | 0.222 | 0.262 | 48.8281057 | 2.24E-06 |
| **9** | 50000 | 0.1 | 5.00E-07 | 0.0005 | 0.0005 | 0.718 | 0.0005 | 0.018 | 0.24 | 57.1569859 | 7.40E-08 |
| **9** | 50000 | 0.25 | 2.50E-08 | 0.0005 | 0.78 | 0.0005 | 0.0005 | 0.006 | 0.488 | 57.7692088 | 5.74E-08 |
| **9** | 50000 | 0.25 | 1.25E-07 | 0.014 | 0.0005 | 0.612 | 0.0005 | 0.846 | 0.292 | 42.7195265 | 2.52E-05 |
| **9** | 50000 | 0.25 | 2.50E-07 | 0.002 | 0.0005 | 0.842 | 0.0005 | 0.222 | 0.266 | 48.8354503 | 2.23E-06 |
| **9** | 50000 | 0.25 | 5.00E-07 | 0.0005 | 0.0005 | 0.684 | 0.0005 | 0.012 | 0.262 | 57.8895283 | 5.46E-08 |
| **9** | 50000 | 0.5 | 2.50E-08 | 0.0005 | 0.804 | 0.0005 | 0.0005 | 0.004 | 0.524 | 58.3771758 | 4.45E-08 |
| **9** | 50000 | 0.5 | 1.25E-07 | 0.018 | 0.004 | 0.55 | 0.0005 | 0.88 | 0.314 | 38.0475591 | 0.00015084 |
| **9** | 50000 | 0.5 | 2.50E-07 | 0.002 | 0.0005 | 0.848 | 0.0005 | 0.23 | 0.31 | 48.4442932 | 2.62E-06 |
| **9** | 50000 | 0.5 | 5.00E-07 | 0.0005 | 0.0005 | 0.736 | 0.0005 | 0.01 | 0.276 | 58.0035143 | 5.20E-08 |
| **10** | 0 | 0 | 2.50E-08 | 0.0005 | 0.802 | 0.0005 | 0.0005 | 0.002 | 0.236 | 61.3637712 | 1.27E-08 |
| **10** | 0 | 0 | 1.25E-07 | 0.026 | 0.008 | 0.566 | 0.0005 | 0.42 | 0.144 | 38.9069574 | 0.00010901 |
| **10** | 0 | 0 | 2.50E-07 | 0.006 | 0.0005 | 0.86 | 0.0005 | 0.012 | 0.094 | 54.5118655 | 2.21E-07 |
| **10** | 0 | 0 | 5.00E-07 | 0.0005 | 0.0005 | 0.714 | 0.0005 | 0.0005 | 0.108 | 65.9322124 | 1.83E-09 |

**Table S3.** mtDNA haplogroup L0 sequences used in this study.

| **SampleID** | **Geography/Ethnicity** | **Reference** |
| --- | --- | --- |
| AF346985 | Chad | [^37^](#_ENREF_37) |
| AF346998 | Congo - Mbuti | [^37^](#_ENREF_37) |
| AF346999 | Congo - Mbuti | [^37^](#_ENREF_37) |
| AF347008 | unknown - San | [^37^](#_ENREF_37) |
| AF347009 | unknown - San | [^37^](#_ENREF_37) |
| AF381988 | Morocco | [^38^](#_ENREF_38) |
| AM711903 | Congo - Mbuti | [^39^](#_ENREF_39) |
| AM711904 | S. Africa - Kung | [^39^](#_ENREF_39) |
| AY195777 | S. Africa - Khoe | [^40^](#_ENREF_40) |
| AY195780 | S. Africa | [^40^](#_ENREF_40) |
| AY963585 | Uganda | [^41^](#_ENREF_41) |
| DQ304897 | USA (African American) | [^42^](#_ENREF_42) |
| DQ304898 | USA (African American) | [^42^](#_ENREF_42) |
| DQ304899 | USA (African American) | [^42^](#_ENREF_42) |
| DQ304900 | USA (African American) | [^42^](#_ENREF_42) |
| DQ304901 | USA (African American) | [^42^](#_ENREF_42) |
| DQ304902 | USA (African American) | [^42^](#_ENREF_42) |
| DQ304903 | USA (African American) | [^42^](#_ENREF_42) |
| DQ304904 | USA (African American) | [^42^](#_ENREF_42) |
| DQ341058 | Dominica | [^43^](#_ENREF_43) |
| EF184586 | Tanzania | [^44^](#_ENREF_44) |
| EF184587 | Tanzania | [^44^](#_ENREF_44) |
| EF184588 | Tanzania | [^44^](#_ENREF_44) |
| EF184589 | Tanzania | [^44^](#_ENREF_44) |
| EF184590 | Tanzania | [^44^](#_ENREF_44) |
| EF184592 | Tanzania | [^44^](#_ENREF_44) |
| EF184593 | South Africa - San | [^44^](#_ENREF_44) |
| EF184594 | South Africa - San | [^44^](#_ENREF_44) |
| EF184599 | Tanzania | [^44^](#_ENREF_44) |
| EF184601 | Tanzania | [^44^](#_ENREF_44) |
| EF184603 | Tanzania | [^44^](#_ENREF_44) |
| EF184604 | Tanzania | [^44^](#_ENREF_44) |
| EF184605 | Tanzania | [^44^](#_ENREF_44) |
| EF184606 | Tanzania | [^44^](#_ENREF_44) |
| EF184610 | Tanzania | [^44^](#_ENREF_44) |
| EF184611 | Tanzania | [^44^](#_ENREF_44) |
| EF556174 | Ethiopia | [^45^](#_ENREF_45) |
| EU092665 | Israel- Bedouin | [^46^](#_ENREF_46) |
| EU092668 | Ethiopia - Jew | [^46^](#_ENREF_46) |
| EU092670 | Ethiopia-Jew | [^46^](#_ENREF_46) |
| EU092688 | Mozambique- Shangaan | [^46^](#_ENREF_46) |
| EU092700 | Mozambique | [^46^](#_ENREF_46) |
| EU092701 | Mozambique- Shangaan | [^46^](#_ENREF_46) |
| EU092708 | Mozambique | [^46^](#_ENREF_46) |
| EU092714 | G. Bissau- Beafada | [^46^](#_ENREF_46) |
| EU092745 | S. Arabia | [^46^](#_ENREF_46) |
| EU092746 | S. Arabia | [^46^](#_ENREF_46) |
| EU092760 | Iran | [^46^](#_ENREF_46) |
| EU092763 | Egypt | [^46^](#_ENREF_46) |
| EU092764 | Egypt | [^46^](#_ENREF_46) |
| EU092786 | Oman | [^46^](#_ENREF_46) |
| EU092787 | Oman | [^46^](#_ENREF_46) |
| EU092792 | Yemen | [^46^](#_ENREF_46) |
| EU092801 | Yemen | [^46^](#_ENREF_46) |
| EU092809 | Yemen | [^46^](#_ENREF_46) |
| EU092810 | Yemen | [^46^](#_ENREF_46) |
| EU092819 | Algeria | [^46^](#_ENREF_46) |
| EU092831 | S. Africa - San | [^46^](#_ENREF_46) |
| EU092832 | S. Africa - San | [^46^](#_ENREF_46) |
| EU092833 | S. Africa - San | [^46^](#_ENREF_46) |
| EU092834 | S. Africa - San | [^46^](#_ENREF_46) |
| EU092835 | S. Africa - San | [^46^](#_ENREF_46) |
| EU092837 | S. Africa - San | [^46^](#_ENREF_46) |
| EU092839 | S. Africa - Khoe | [^46^](#_ENREF_46) |
| EU092840 | S. Africa - Khoe | [^46^](#_ENREF_46) |
| EU092841 | S. Africa - Khoe | [^46^](#_ENREF_46) |
| EU092842 | S. Africa - Khoe | [^46^](#_ENREF_46) |
| EU092843 | S. Africa - Khoe | [^46^](#_ENREF_46) |
| EU092844 | S. Africa - Khoe | [^46^](#_ENREF_46) |
| EU092845 | S. Africa - Khoe | [^46^](#_ENREF_46) |
| EU092846 | S. Africa - Khoe | [^46^](#_ENREF_46) |
| EU092853 | S. Africa - San | [^46^](#_ENREF_46) |
| EU092855 | S. Africa - San | [^46^](#_ENREF_46) |
| EU092856 | S. Africa - San | [^46^](#_ENREF_46) |
| EU092858 | S. Africa - San | [^46^](#_ENREF_46) |
| EU092859 | S. Africa | [^46^](#_ENREF_46) |
| EU092860 | S. Africa | [^46^](#_ENREF_46) |
| EU092861 | S. Africa - SWB | [^46^](#_ENREF_46) |
| EU092863 | South Africa | [^46^](#_ENREF_46) |
| EU092868 | S. Africa - SWB | [^46^](#_ENREF_46) |
| EU092869 | S. Africa - SWB | [^46^](#_ENREF_46) |
| EU092870 | S. Africa | [^46^](#_ENREF_46) |
| EU092871 | S. Africa- SEB | [^46^](#_ENREF_46) |
| EU092874 | S. Africa | [^46^](#_ENREF_46) |
| EU092878 | Chad - Laal | [^46^](#_ENREF_46) |
| EU092881 | Chad - Laal | [^46^](#_ENREF_46) |
| EU092889 | Chad - Sara | [^46^](#_ENREF_46) |
| EU092892 | Chad - Sara | [^46^](#_ENREF_46) |
| EU092900 | Chad - Sara | [^46^](#_ENREF_46) |
| EU092906 | Kenya | [^46^](#_ENREF_46) |
| EU092909 | Kenya | [^46^](#_ENREF_46) |
| EU092911 | Kenya | [^46^](#_ENREF_46) |
| EU092913 | Kenya | [^46^](#_ENREF_46) |
| EU092921 | Kuwait | [^46^](#_ENREF_46) |
| EU092925 | Oman | [^46^](#_ENREF_46) |
| EU092936 | Ethiopia | [^46^](#_ENREF_46) |
| EU092945 | Ethiopia | [^46^](#_ENREF_46) |
| EU092950 | Ethiopia | [^46^](#_ENREF_46) |
| EU092963 | USA | [^46^](#_ENREF_46) |
| EU092964 | USA | [^46^](#_ENREF_46) |
| EU092965 | S. Africa - San | [^46^](#_ENREF_46) |
| EU092966 | S. Africa - Khoe | [^46^](#_ENREF_46) |
| EU597502 | S. Africa | [^47^](#_ENREF_47) |
| EU597514 | Congo | [^47^](#_ENREF_47) |
| EU597537 | Congo - Mbuti | [^47^](#_ENREF_47) |
| EU935434 | Egypt - el-Hayez oasis | [^48^](#_ENREF_48) |
| EU935437 | Egypt - el-Hayez oasis | [^48^](#_ENREF_48) |
| EU935464 | Egypt - el-Hayez oasis | [^48^](#_ENREF_48) |
| EU935467 | Egypt - el-Hayez oasis | [^48^](#_ENREF_48) |
| FJ157838 | India | [^49^](#_ENREF_49) |
| FJ157839 | India | [^49^](#_ENREF_49) |
| FJ157840 | India | [^49^](#_ENREF_49) |
| HG01108 | Puerto Rico | 1K GP Database^a^ |
| HG02971 | Nigeria- Esan | 1K GP database^a^ |
| HG02981 | Nigeria- Esan | 1K GP database^a^ |
| HG03063 | Sierra Leone - Mende | 1K GP database^a^ |
| HG03084 | Sierra Leone - Mende | 1K GP database^a^ |
| HG03127 | Nigeria - Esan | 1K GP database^a^ |
| HG03267 | Nigeria - Esan | 1K GP database^a^ |
| HG03268 | Nigeria - Esan | 1K GP database^a^ |
| HG03298 | Nigeria - Esan | 1K GP database^a^ |
| HG03517 | Nigeria - Esan | 1K GP database^a^ |
| HM771160 | CAR - Pygmy | [^50^](#_ENREF_50) |
| HM771161 | CAR - Pygmy | [^50^](#_ENREF_50) |
| HM771188 | Congo - Mbuti | [^50^](#_ENREF_50) |
| HM771189 | Congo - Mbuti | [^50^](#_ENREF_50) |
| HM771190 | Congo - Mbuti | [^50^](#_ENREF_50) |
| HM771199 | Congo - Mbuti | [^50^](#_ENREF_50) |
| HM771200 | Congo - Mbuti | [^50^](#_ENREF_50) |
| HM771201 | Congo - Mbuti | [^50^](#_ENREF_50) |
| HM771202 | Congo - Mbuti | [^50^](#_ENREF_50) |
| JQ044838 | Burkina Fasso | [^51^](#_ENREF_51) |
| JQ044849 | Burkina Fasso | [^51^](#_ENREF_51) |
| JQ044851 | Burkina Fasso | [^51^](#_ENREF_51) |
| JQ044874 | Burkina Fasso | [^51^](#_ENREF_51) |
| JQ044893 | Burkina Fasso | [^51^](#_ENREF_51) |
| JQ044903 | Burkina Fasso | [^51^](#_ENREF_51) |
| JQ044943 | Burkina Fasso | [^51^](#_ENREF_51) |
| JQ044995 | Burkina Fasso | [^51^](#_ENREF_51) |
| JQ045004 | Burkina Fasso | [^51^](#_ENREF_51) |
| JQ045053 | Burkina Fasso | [^51^](#_ENREF_51) |
| JQ702227 | unknown | [^52^](#_ENREF_52) |
| JQ702326 | unknown | [^52^](#_ENREF_52) |
| JQ702428 | unknown | [^52^](#_ENREF_52) |
| JQ703481 | unknown | [^52^](#_ENREF_52) |
| JQ705109 | Dhamar (Yemen) | [^52^](#_ENREF_52) |
| JX303745 | Zambia | [^53^](#_ENREF_53) |
| JX303753 | Zambia | [^53^](#_ENREF_53) |
| JX303757 | Zambia | [^53^](#_ENREF_53) |
| JX303762 | Zambia - Fwe | [^53^](#_ENREF_53) |
| JX303763 | Zambia - Totela | [^53^](#_ENREF_53) |
| JX303765 | Zambia | [^53^](#_ENREF_53) |
| JX303766 | Zambia - Shanjo | [^53^](#_ENREF_53) |
| JX303772 | Zambia - Totela | [^53^](#_ENREF_53) |
| JX303778 | Zambia - Totela | [^53^](#_ENREF_53) |
| JX303784 | Zambia - Tonga | [^53^](#_ENREF_53) |
| JX303786 | Zambia - Tonga | [^53^](#_ENREF_53) |
| JX303788 | Zambia | [^53^](#_ENREF_53) |
| JX303791 | Zambia | [^53^](#_ENREF_53) |
| JX303796 | Zambia - Kwamashi | [^53^](#_ENREF_53) |
| JX303817 | Zambia - Fwe | [^53^](#_ENREF_53) |
| JX303818 | Zambia | [^53^](#_ENREF_53) |
| JX303823 | Zambia - Fwe | [^53^](#_ENREF_53) |
| JX303826 | Zambia - Kwamashi | [^53^](#_ENREF_53) |
| JX303830 | Zambia - Kwamashi | [^53^](#_ENREF_53) |
| JX303831 | Zambia - Kwamashi | [^53^](#_ENREF_53) |
| JX303835 | Zambia - Kwamashi | [^53^](#_ENREF_53) |
| JX303856 | Zambia | [^53^](#_ENREF_53) |
| JX303861 | Zambia | [^53^](#_ENREF_53) |
| JX303865 | Zambia | [^53^](#_ENREF_53) |
| JX303867 | Zambia | [^53^](#_ENREF_53) |
| JX303868 | Zambia | [^53^](#_ENREF_53) |
| JX303869 | Zambia | [^53^](#_ENREF_53) |
| JX303895 | Zambia | [^53^](#_ENREF_53) |
| JX303897 | Zambia | [^53^](#_ENREF_53) |
| JX303899 | Zambia | [^53^](#_ENREF_53) |
| JX303903 | Zambia | [^53^](#_ENREF_53) |
| JX303904 | Zambia - Totela | [^53^](#_ENREF_53) |
| JX303911 | Zambia -Tonga | [^53^](#_ENREF_53) |
| KC345764 | Angola | [^54^](#_ENREF_54) |
| KC345765 | Angola | [^54^](#_ENREF_54) |
| KC345766 | Angola | [^54^](#_ENREF_54) |
| KC345767 | Angola | [^54^](#_ENREF_54) |
| KC345768 | Angola | [^54^](#_ENREF_54) |
| KC345769 | Angola | [^54^](#_ENREF_54) |
| KC345770 | Angola | [^54^](#_ENREF_54) |
| KC345771 | Angola | [^54^](#_ENREF_54) |
| KC345772 | Angola | [^54^](#_ENREF_54) |
| KC345773 | Angola | [^54^](#_ENREF_54) |
| KC345774 | Angola | [^54^](#_ENREF_54) |
| KC345775 | Angola | [^54^](#_ENREF_54) |
| KC345776 | Angola | [^54^](#_ENREF_54) |
| KC345777 | Angola | [^54^](#_ENREF_54) |
| KC345778 | Angola | [^54^](#_ENREF_54) |
| KC345779 | Angola | [^54^](#_ENREF_54) |
| KC345780 | Angola | [^54^](#_ENREF_54) |
| KC345781 | Angola | [^54^](#_ENREF_54) |
| KC345782 | Angola | [^54^](#_ENREF_54) |
| KC345783 | Angola | [^54^](#_ENREF_54) |
| KC345784 | Angola | [^54^](#_ENREF_54) |
| KC345785 | Angola | [^54^](#_ENREF_54) |
| KC345786 | Botswana | [^54^](#_ENREF_54) |
| KC345787 | Botswana - Khoe | [^54^](#_ENREF_54) |
| KC345788 | Botswana - Khoe | [^54^](#_ENREF_54) |
| KC345789 | Botswana - Khoe | [^54^](#_ENREF_54) |
| KC345790 | Botswana - Khoe | [^54^](#_ENREF_54) |
| KC345791 | Botswana - Khoe | [^54^](#_ENREF_54) |
| KC345792 | Botswana - Khoe | [^54^](#_ENREF_54) |
| KC345793 | Botswana - Khoe | [^54^](#_ENREF_54) |
| KC345794 | Botswana - Khoe | [^54^](#_ENREF_54) |
| KC345795 | Botswana - Khoe | [^54^](#_ENREF_54) |
| KC345796 | Botswana - Khoe | [^54^](#_ENREF_54) |
| KC345797 | Botswana - Khoe | [^54^](#_ENREF_54) |
| KC345798 | Botswana - Khoe | [^54^](#_ENREF_54) |
| KC345799 | Botswana - Khoe | [^54^](#_ENREF_54) |
| KC345800 | Botswana - Khoe | [^54^](#_ENREF_54) |
| KC345801 | Botswana - Khoe | [^54^](#_ENREF_54) |
| KC345802 | Botswana - Khoe | [^54^](#_ENREF_54) |
| KC345803 | Botswana - Khoe | [^54^](#_ENREF_54) |
| KC345804 | Botswana - Khoe | [^54^](#_ENREF_54) |
| KC345805 | Botswana - Khoe | [^54^](#_ENREF_54) |
| KC345806 | Botswana - Khoe | [^54^](#_ENREF_54) |
| KC345807 | Botswana - Khoe | [^54^](#_ENREF_54) |
| KC345808 | Botswana - Khoe | [^54^](#_ENREF_54) |
| KC345809 | Botswana - Kx'a | [^54^](#_ENREF_54) |
| KC345810 | Botswana- Khoe | [^54^](#_ENREF_54) |
| KC345811 | Botswana- Khoe | [^54^](#_ENREF_54) |
| KC345812 | Botswana - Khoe | [^54^](#_ENREF_54) |
| KC345813 | Botswana - Khoe | [^54^](#_ENREF_54) |
| KC345814 | Botswana - Khoe | [^54^](#_ENREF_54) |
| KC345815 | Botswana - Khoe | [^54^](#_ENREF_54) |
| KC345816 | Botswana - Khoe | [^54^](#_ENREF_54) |
| KC345817 | Botswana - Khoe | [^54^](#_ENREF_54) |
| KC345818 | Botswana - Khoe | [^54^](#_ENREF_54) |
| KC345819 | Botswana - Khoe | [^54^](#_ENREF_54) |
| KC345820 | Botswana - Khoe | [^54^](#_ENREF_54) |
| KC345821 | Botswana - Khoe | [^54^](#_ENREF_54) |
| KC345822 | Botswana - Khoe | [^54^](#_ENREF_54) |
| KC345823 | Botswana - Khoe | [^54^](#_ENREF_54) |
| KC345824 | Botswana - Khoe | [^54^](#_ENREF_54) |
| KC345825 | Botswana - Khoe | [^54^](#_ENREF_54) |
| KC345826 | Botswana - Khoe | [^54^](#_ENREF_54) |
| KC345827 | Botswana - Khoe | [^54^](#_ENREF_54) |
| KC345828 | Botswana - Khoe | [^54^](#_ENREF_54) |
| KC345829 | Botswana - G\|ui | [^54^](#_ENREF_54) |
| KC345830 | Botswana - Khoe | [^54^](#_ENREF_54) |
| KC345831 | Botswana - Khoe | [^54^](#_ENREF_54) |
| KC345832 | Botswana - Khoe | [^54^](#_ENREF_54) |
| KC345833 | Botswana - Khoe | [^54^](#_ENREF_54) |
| KC345834 | Botswana - Khoe | [^54^](#_ENREF_54) |
| KC345835 | Botswana - Khoe | [^54^](#_ENREF_54) |
| KC345836 | Botswana - Khoe | [^54^](#_ENREF_54) |
| KC345837 | Botswana - Khoe | [^54^](#_ENREF_54) |
| KC345838 | Botswana - Khoe | [^54^](#_ENREF_54) |
| KC345839 | Botswana - Khoe | [^54^](#_ENREF_54) |
| KC345840 | Botswana - Khoe | [^54^](#_ENREF_54) |
| KC345841 | Botswana - Khoe | [^54^](#_ENREF_54) |
| KC345842 | Botswana - Khoe | [^54^](#_ENREF_54) |
| KC345843 | Botswana - Khoe | [^54^](#_ENREF_54) |
| KC345844 | Botswana - Khoe | [^54^](#_ENREF_54) |
| KC345845 | Botswana - Khoe | [^54^](#_ENREF_54) |
| KC345846 | Botswana - Khoe | [^54^](#_ENREF_54) |
| KC345847 | Botswana - Khoe | [^54^](#_ENREF_54) |
| KC345848 | Botswana - Khoe | [^54^](#_ENREF_54) |
| KC345849 | Botswana - Khoe | [^54^](#_ENREF_54) |
| KC345850 | Botswana - Khoe | [^54^](#_ENREF_54) |
| KC345851 | Botswana - Khoe | [^54^](#_ENREF_54) |
| KC345852 | Botswana - Kx'a | [^54^](#_ENREF_54) |
| KC345853 | Botswana - Kx'a | [^54^](#_ENREF_54) |
| KC345854 | Botswana - Kx'a | [^54^](#_ENREF_54) |
| KC345855 | Botswana - Kx'a | [^54^](#_ENREF_54) |
| KC345856 | Botswana - Kx'a | [^54^](#_ENREF_54) |
| KC345857 | Botswana - Kx'a | [^54^](#_ENREF_54) |
| KC345858 | Botswana - Kx'a | [^54^](#_ENREF_54) |
| KC345859 | Botswana - Kx'a | [^54^](#_ENREF_54) |
| KC345860 | Botswana - Kx'a | [^54^](#_ENREF_54) |
| KC345861 | Botswana - Kx'a | [^54^](#_ENREF_54) |
| KC345862 | Botswana - Kx'a | [^54^](#_ENREF_54) |
| KC345863 | Botswana - Kx'a | [^54^](#_ENREF_54) |
| KC345864 | Botswana - Kx'a | [^54^](#_ENREF_54) |
| KC345865 | Botswana - Kx'a | [^54^](#_ENREF_54) |
| KC345866 | Botswana - Kx'a | [^54^](#_ENREF_54) |
| KC345867 | Botswana - Kx'a | [^54^](#_ENREF_54) |
| KC345868 | Botswana - Kx'a | [^54^](#_ENREF_54) |
| KC345869 | Botswana - Kx'a | [^54^](#_ENREF_54) |
| KC345870 | Botswana - Kx'a | [^54^](#_ENREF_54) |
| KC345871 | Botswana - Kx'a | [^54^](#_ENREF_54) |
| KC345872 | Botswana - Kx'a | [^54^](#_ENREF_54) |
| KC345873 | Botswana - Kx'a | [^54^](#_ENREF_54) |
| KC345874 | Botswana - Kx'a | [^54^](#_ENREF_54) |
| KC345875 | Botswana - Kx'a | [^54^](#_ENREF_54) |
| KC345876 | Botswana - Kx'a | [^54^](#_ENREF_54) |
| KC345877 | Botswana - Kx'a | [^54^](#_ENREF_54) |
| KC345878 | Botswana - Kx'a | [^54^](#_ENREF_54) |
| KC345879 | Botswana - Kx'a | [^54^](#_ENREF_54) |
| KC345880 | Botswana - Kx'a | [^54^](#_ENREF_54) |
| KC345881 | Botswana - Kx'a | [^54^](#_ENREF_54) |
| KC345882 | Botswana - Kx'a | [^54^](#_ENREF_54) |
| KC345883 | Botswana - Kx'a | [^54^](#_ENREF_54) |
| KC345884 | Botswana - Kx'a | [^54^](#_ENREF_54) |
| KC345885 | Botswana - Kx'a | [^54^](#_ENREF_54) |
| KC345886 | Botswana - Kx'a | [^54^](#_ENREF_54) |
| KC345887 | Botswana - Kx'a | [^54^](#_ENREF_54) |
| KC345888 | Botswana - Kx'a | [^54^](#_ENREF_54) |
| KC345889 | Botswana - Kx'a | [^54^](#_ENREF_54) |
| KC345890 | Botswana - Kx'a | [^54^](#_ENREF_54) |
| KC345891 | Botswana - Kx'a | [^54^](#_ENREF_54) |
| KC345892 | Botswana - Kx'a | [^54^](#_ENREF_54) |
| KC345893 | Botswana - Kx'a | [^54^](#_ENREF_54) |
| KC345894 | Botswana - Kx'a | [^54^](#_ENREF_54) |
| KC345895 | Botswana - Kx'a | [^54^](#_ENREF_54) |
| KC345896 | Botswana - Kx'a | [^54^](#_ENREF_54) |
| KC345897 | Botswana - Kx'a | [^54^](#_ENREF_54) |
| KC345898 | Botswana - Kx'a | [^54^](#_ENREF_54) |
| KC345899 | Botswana - Kx'a | [^54^](#_ENREF_54) |
| KC345900 | Botswana - Kx'a | [^54^](#_ENREF_54) |
| KC345901 | Botswana - Kx'a | [^54^](#_ENREF_54) |
| KC345902 | Botswana - Kx'a | [^54^](#_ENREF_54) |
| KC345903 | Botswana - Kx'a | [^54^](#_ENREF_54) |
| KC345904 | Botswana - Kx'a | [^54^](#_ENREF_54) |
| KC345905 | Botswana - Kx'a | [^54^](#_ENREF_54) |
| KC345906 | Botswana - Kx'a | [^54^](#_ENREF_54) |
| KC345907 | Botswana - Kx'a | [^54^](#_ENREF_54) |
| KC345908 | Botswana - Kx'a | [^54^](#_ENREF_54) |
| KC345909 | Botswana - Kx'a | [^54^](#_ENREF_54) |
| KC345910 | Botswana - Kx'a | [^54^](#_ENREF_54) |
| KC345911 | Botswana - Kx'a | [^54^](#_ENREF_54) |
| KC345912 | Botswana - Kx'a | [^54^](#_ENREF_54) |
| KC345913 | Botswana - Ju\|hoan_South | [^54^](#_ENREF_54) |
| KC345914 | Botswana - Kx'a | [^54^](#_ENREF_54) |
| KC345915 | Botswana - Kx'a | [^54^](#_ENREF_54) |
| KC345916 | Botswana - Kx'a | [^54^](#_ENREF_54) |
| KC345917 | Botswana | [^54^](#_ENREF_54) |
| KC345918 | Botswana | [^54^](#_ENREF_54) |
| KC345919 | Botswana | [^54^](#_ENREF_54) |
| KC345920 | Botswana | [^54^](#_ENREF_54) |
| KC345921 | Botswana | [^54^](#_ENREF_54) |
| KC345922 | Botswana | [^54^](#_ENREF_54) |
| KC345923 | Botswana | [^54^](#_ENREF_54) |
| KC345924 | Botswana | [^54^](#_ENREF_54) |
| KC345925 | Botswana | [^54^](#_ENREF_54) |
| KC345926 | Botswana | [^54^](#_ENREF_54) |
| KC345927 | Botswana | [^54^](#_ENREF_54) |
| KC345928 | Botswana | [^54^](#_ENREF_54) |
| KC345929 | Botswana | [^54^](#_ENREF_54) |
| KC345930 | Botswana | [^54^](#_ENREF_54) |
| KC345931 | Botswana | [^54^](#_ENREF_54) |
| KC345932 | Botswana | [^54^](#_ENREF_54) |
| KC345933 | Botswana | [^54^](#_ENREF_54) |
| KC345934 | Botswana - Khoe | [^54^](#_ENREF_54) |
| KC345935 | Botswana - Khoe | [^54^](#_ENREF_54) |
| KC345936 | Botswana - Khoe | [^54^](#_ENREF_54) |
| KC345937 | Botswana - Khoe | [^54^](#_ENREF_54) |
| KC345938 | Botswana - Khoe | [^54^](#_ENREF_54) |
| KC345939 | Botswana - Khoe | [^54^](#_ENREF_54) |
| KC345940 | Botswana - Khoe | [^54^](#_ENREF_54) |
| KC345941 | Botswana - Khoe | [^54^](#_ENREF_54) |
| KC345942 | Botswana - Khoe | [^54^](#_ENREF_54) |
| KC345943 | Botswana - Khoe | [^54^](#_ENREF_54) |
| KC345944 | Botswana - Khoe | [^54^](#_ENREF_54) |
| KC345945 | Botswana - Khoe | [^54^](#_ENREF_54) |
| KC345946 | Botswana - Khoe | [^54^](#_ENREF_54) |
| KC345947 | Botswana - Khoe | [^54^](#_ENREF_54) |
| KC345948 | Botswana - Khoe | [^54^](#_ENREF_54) |
| KC345949 | Botswana - Khoe | [^54^](#_ENREF_54) |
| KC345950 | Botswana - Khoe | [^54^](#_ENREF_54) |
| KC345951 | Botswana - Khoe | [^54^](#_ENREF_54) |
| KC345952 | Botswana - Khoe | [^54^](#_ENREF_54) |
| KC345953 | Botswana - Khoe | [^54^](#_ENREF_54) |
| KC345954 | Botswana - Khoe | [^54^](#_ENREF_54) |
| KC345955 | Botswana - Khoe | [^54^](#_ENREF_54) |
| KC345956 | Botswana - Khoe | [^54^](#_ENREF_54) |
| KC345957 | Botswana - Khoe | [^54^](#_ENREF_54) |
| KC345958 | Namibia - Khoe | [^54^](#_ENREF_54) |
| KC345959 | Botswana - Khoe | [^54^](#_ENREF_54) |
| KC345960 | Botswana - Khoe | [^54^](#_ENREF_54) |
| KC345961 | Botswana - Khoe | [^54^](#_ENREF_54) |
| KC345962 | Botswana - Naro | [^54^](#_ENREF_54) |
| KC345963 | Botswana - Khoe | [^54^](#_ENREF_54) |
| KC345964 | Botswana - Khoe | [^54^](#_ENREF_54) |
| KC345965 | Botswana - Khoe | [^54^](#_ENREF_54) |
| KC345966 | Botswana - Khoe | [^54^](#_ENREF_54) |
| KC345967 | Botswana - Khoe | [^54^](#_ENREF_54) |
| KC345968 | Botswana - Khoe | [^54^](#_ENREF_54) |
| KC345969 | Botswana- Khoe | [^54^](#_ENREF_54) |
| KC345970 | Botswana- Khoe | [^54^](#_ENREF_54) |
| KC345971 | Botswana- Khoe | [^54^](#_ENREF_54) |
| KC345972 | Botswana- Khoe | [^54^](#_ENREF_54) |
| KC345973 | Botswana- Khoe | [^54^](#_ENREF_54) |
| KC345974 | Botswana - Khoe | [^54^](#_ENREF_54) |
| KC345975 | Botswana - Khoe | [^54^](#_ENREF_54) |
| KC345976 | Botswana - Khoe | [^54^](#_ENREF_54) |
| KC345977 | Botswana - Khoe | [^54^](#_ENREF_54) |
| KC345978 | Botswana - Khoe | [^54^](#_ENREF_54) |
| KC345979 | Botswana - Khoe | [^54^](#_ENREF_54) |
| KC345980 | Botswana - Khoe | [^54^](#_ENREF_54) |
| KC345981 | Botswana - Khoe | [^54^](#_ENREF_54) |
| KC345982 | Botswana - Khoe | [^54^](#_ENREF_54) |
| KC345983 | Botswana - Tuu | [^54^](#_ENREF_54) |
| KC345984 | Botswana - Tuu | [^54^](#_ENREF_54) |
| KC345985 | Botswana - Tuu | [^54^](#_ENREF_54) |
| KC345986 | Botswana - Tuu | [^54^](#_ENREF_54) |
| KC345987 | Botswana - Tuu | [^54^](#_ENREF_54) |
| KC345988 | Botswana - Tuu | [^54^](#_ENREF_54) |
| KC345989 | Botswana - Tuu | [^54^](#_ENREF_54) |
| KC345990 | Botswana - Tuu | [^54^](#_ENREF_54) |
| KC345991 | Botswana - Tuu | [^54^](#_ENREF_54) |
| KC345992 | Botswana - Tuu | [^54^](#_ENREF_54) |
| KC345993 | Botswana - Tuu | [^54^](#_ENREF_54) |
| KC345994 | Botswana - Tuu | [^54^](#_ENREF_54) |
| KC345995 | Botswana- Khoe | [^54^](#_ENREF_54) |
| KC345996 | Botswana - Tuu | [^54^](#_ENREF_54) |
| KC345997 | Botswana - Tuu | [^54^](#_ENREF_54) |
| KC345998 | Botswana - Tuu | [^54^](#_ENREF_54) |
| KC345999 | Botswana - Tuu | [^54^](#_ENREF_54) |
| KC346000 | Botswana - Tuu | [^54^](#_ENREF_54) |
| KC346001 | Botswana - Tuu | [^54^](#_ENREF_54) |
| KC346002 | Botswana- Taa_east | [^54^](#_ENREF_54) |
| KC346003 | Botswana - Tuu | [^54^](#_ENREF_54) |
| KC346004 | Botswana - Tuu | [^54^](#_ENREF_54) |
| KC346005 | Botswana - Tuu | [^54^](#_ENREF_54) |
| KC346006 | Botswana - Tuu | [^54^](#_ENREF_54) |
| KC346007 | Botswana - Tuu | [^54^](#_ENREF_54) |
| KC346008 | Botswana - Tuu | [^54^](#_ENREF_54) |
| KC346009 | Botswana - Tuu | [^54^](#_ENREF_54) |
| KC346010 | Botswana - Tuu | [^54^](#_ENREF_54) |
| KC346011 | Botswana - Tuu | [^54^](#_ENREF_54) |
| KC346012 | Botswana - Tuu | [^54^](#_ENREF_54) |
| KC346013 | Botswana - Tuu | [^54^](#_ENREF_54) |
| KC346014 | Botswana - Tuu | [^54^](#_ENREF_54) |
| KC346015 | Botswana - Tuu | [^54^](#_ENREF_54) |
| KC346016 | Botswana - Tuu | [^54^](#_ENREF_54) |
| KC346017 | Botswana - Tuu | [^54^](#_ENREF_54) |
| KC346018 | Botswana - Tuu | [^54^](#_ENREF_54) |
| KC346019 | Botswana - Tuu | [^54^](#_ENREF_54) |
| KC346020 | Botswana - Tuu | [^54^](#_ENREF_54) |
| KC346021 | Botswana - Tuu | [^54^](#_ENREF_54) |
| KC346022 | Botswana - Tuu | [^54^](#_ENREF_54) |
| KC346023 | Botswana - Tuu | [^54^](#_ENREF_54) |
| KC346024 | Botswana - Tuu | [^54^](#_ENREF_54) |
| KC346025 | Botswana - Tuu | [^54^](#_ENREF_54) |
| KC346026 | Botswana - Tuu | [^54^](#_ENREF_54) |
| KC346027 | Botswana - Tuu | [^54^](#_ENREF_54) |
| KC346028 | Botswana - Tuu | [^54^](#_ENREF_54) |
| KC346029 | Botswana - Tuu | [^54^](#_ENREF_54) |
| KC346030 | Botswana - Tuu | [^54^](#_ENREF_54) |
| KC346031 | Botswana - Tuu | [^54^](#_ENREF_54) |
| KC346032 | Botswana - Tuu | [^54^](#_ENREF_54) |
| KC346033 | Botswana - Tuu | [^54^](#_ENREF_54) |
| KC346034 | Botswana - Tuu | [^54^](#_ENREF_54) |
| KC346035 | Botswana - Tuu | [^54^](#_ENREF_54) |
| KC346036 | Botswana - Tuu | [^54^](#_ENREF_54) |
| KC346037 | Botswana - Tuu | [^54^](#_ENREF_54) |
| KC346038 | Botswana - Tuu | [^54^](#_ENREF_54) |
| KC346039 | Botswana - Tuu | [^54^](#_ENREF_54) |
| KC346040 | Botswana - Tuu | [^54^](#_ENREF_54) |
| KC346041 | Botswana - Tuu | [^54^](#_ENREF_54) |
| KC346042 | Botswana - Tuu | [^54^](#_ENREF_54) |
| KC346043 | Botswana - Tuu | [^54^](#_ENREF_54) |
| KC346044 | Botswana - Tuu | [^54^](#_ENREF_54) |
| KC346045 | Botswana - Tuu | [^54^](#_ENREF_54) |
| KC346046 | Botswana - Tuu | [^54^](#_ENREF_54) |
| KC346047 | Botswana - Tuu | [^54^](#_ENREF_54) |
| KC346048 | Botswana - Tuu | [^54^](#_ENREF_54) |
| KC346049 | Botswana - Tuu | [^54^](#_ENREF_54) |
| KC346050 | Botswana - Tuu | [^54^](#_ENREF_54) |
| KC346051 | Botswana - Tuu | [^54^](#_ENREF_54) |
| KC346052 | Botswana - Tuu | [^54^](#_ENREF_54) |
| KC346053 | Botswana - Tuu | [^54^](#_ENREF_54) |
| KC346054 | Botswana - Tuu | [^54^](#_ENREF_54) |
| KC346055 | Botswana - Tuu | [^54^](#_ENREF_54) |
| KC346056 | Botswana - Tuu | [^54^](#_ENREF_54) |
| KC346057 | Botswana - Tuu | [^54^](#_ENREF_54) |
| KC346058 | Botswana - Tuu | [^54^](#_ENREF_54) |
| KC346059 | Botswana - Tuu | [^54^](#_ENREF_54) |
| KC346060 | Botswana - Tuu | [^54^](#_ENREF_54) |
| KC346061 | Botswana - Tuu | [^54^](#_ENREF_54) |
| KC346062 | Botswana - Tuu | [^54^](#_ENREF_54) |
| KC346063 | Botswana - Tuu | [^54^](#_ENREF_54) |
| KC346064 | Botswana - Tuu | [^54^](#_ENREF_54) |
| KC346065 | Botswana - Tuu | [^54^](#_ENREF_54) |
| KC346066 | Botswana - Khoe | [^54^](#_ENREF_54) |
| KC346067 | Botswana - Khoe | [^54^](#_ENREF_54) |
| KC346068 | Botswana - Khoe | [^54^](#_ENREF_54) |
| KC346069 | Botswana - Khoe | [^54^](#_ENREF_54) |
| KC346070 | Botswana - Khoe | [^54^](#_ENREF_54) |
| KC346071 | Botswana - Khoe | [^54^](#_ENREF_54) |
| KC346072 | Botswana - Khoe | [^54^](#_ENREF_54) |
| KC346073 | Botswana - Khoe | [^54^](#_ENREF_54) |
| KC346074 | Botswana - Khoe | [^54^](#_ENREF_54) |
| KC346075 | Botswana - Khoe | [^54^](#_ENREF_54) |
| KC346076 | Botswana - Khoe | [^54^](#_ENREF_54) |
| KC346077 | Botswana - Tshwa | [^54^](#_ENREF_54) |
| KC346078 | Botswana - Khoe | [^54^](#_ENREF_54) |
| KC346079 | Botswana - Khoe | [^54^](#_ENREF_54) |
| KC346080 | Botswana - Khoe | [^54^](#_ENREF_54) |
| KC346081 | Botswana - Khoe | [^54^](#_ENREF_54) |
| KC346082 | Botswana - Khoe | [^54^](#_ENREF_54) |
| KC346083 | Botswana - Khoe | [^54^](#_ENREF_54) |
| KC346084 | Botswana - Khoe | [^54^](#_ENREF_54) |
| KC346085 | Botswana - Khoe | [^54^](#_ENREF_54) |
| KC346086 | Botswana - Khoe | [^54^](#_ENREF_54) |
| KC346087 | Botswana | [^54^](#_ENREF_54) |
| KC346088 | Botswana | [^54^](#_ENREF_54) |
| KC346089 | Botswana | [^54^](#_ENREF_54) |
| KC346090 | Botswana | [^54^](#_ENREF_54) |
| KC346091 | Botswana | [^54^](#_ENREF_54) |
| KC346092 | Namibia - Khoe | [^54^](#_ENREF_54) |
| KC346093 | Namibia - Khoe | [^54^](#_ENREF_54) |
| KC346094 | Namibia - Khoe | [^54^](#_ENREF_54) |
| KC346095 | Namibia - Khoe | [^54^](#_ENREF_54) |
| KC346096 | Namibia - Khoe | [^54^](#_ENREF_54) |
| KC346097 | Namibia - Khoe | [^54^](#_ENREF_54) |
| KC346098 | Namibia - Khoe | [^54^](#_ENREF_54) |
| KC346099 | Namibia - Khoe | [^54^](#_ENREF_54) |
| KC346100 | Namibia - Khoe | [^54^](#_ENREF_54) |
| KC346101 | Namibia - Khoe | [^54^](#_ENREF_54) |
| KC346102 | Namibia - Khoe | [^54^](#_ENREF_54) |
| KC346103 | Namibia - Khoe | [^54^](#_ENREF_54) |
| KC346104 | Namibia - Khoe | [^54^](#_ENREF_54) |
| KC346105 | Namibia - Khoe | [^54^](#_ENREF_54) |
| KC346106 | Namibia - Khoe | [^54^](#_ENREF_54) |
| KC346107 | Namibia - Khoe | [^54^](#_ENREF_54) |
| KC346108 | Namibia - Khoe | [^54^](#_ENREF_54) |
| KC346109 | Namibia - Khoe | [^54^](#_ENREF_54) |
| KC346110 | Namibia - Khoe | [^54^](#_ENREF_54) |
| KC346111 | Namibia - Khoe | [^54^](#_ENREF_54) |
| KC346112 | Namibia - Khoe | [^54^](#_ENREF_54) |
| KC346113 | Namibia - Khoe | [^54^](#_ENREF_54) |
| KC346114 | Namibia - Khoe | [^54^](#_ENREF_54) |
| KC346115 | Namibia - Khoe | [^54^](#_ENREF_54) |
| KC346116 | Namibia - Khoe | [^54^](#_ENREF_54) |
| KC346117 | Namibia - Khoe | [^54^](#_ENREF_54) |
| KC346118 | Botswana- Khoe | [^54^](#_ENREF_54) |
| KC346119 | Namibia - Khoe | [^54^](#_ENREF_54) |
| KC346120 | Namibia - Khoe | [^54^](#_ENREF_54) |
| KC346121 | Namibia - Khoe | [^54^](#_ENREF_54) |
| KC346122 | Namibia - Khoe | [^54^](#_ENREF_54) |
| KC346123 | Namibia - Khoe | [^54^](#_ENREF_54) |
| KC346124 | Namibia - Khoe | [^54^](#_ENREF_54) |
| KC346125 | Namibia - Khoe | [^54^](#_ENREF_54) |
| KC346126 | Namibia - Khoe | [^54^](#_ENREF_54) |
| KC346127 | Namibia - Khoe | [^54^](#_ENREF_54) |
| KC346128 | Namibia - Khoe | [^54^](#_ENREF_54) |
| KC346129 | Namibia - Khoe | [^54^](#_ENREF_54) |
| KC346130 | Namibia - Khoe | [^54^](#_ENREF_54) |
| KC346131 | Namibia - Khoe | [^54^](#_ENREF_54) |
| KC346132 | Namibia - Khoe | [^54^](#_ENREF_54) |
| KC346133 | Namibia - Khoe | [^54^](#_ENREF_54) |
| KC346134 | Namibia - Khoe | [^54^](#_ENREF_54) |
| KC346135 | Namibia - Khoe | [^54^](#_ENREF_54) |
| KC346136 | Namibia - Khoe | [^54^](#_ENREF_54) |
| KC346137 | Namibia - Khoe | [^54^](#_ENREF_54) |
| KC346138 | Namibia - Khoe | [^54^](#_ENREF_54) |
| KC346139 | Namibia - Khoe | [^54^](#_ENREF_54) |
| KC346140 | Namibia - Khoe | [^54^](#_ENREF_54) |
| KC346141 | Namibia - Khoe | [^54^](#_ENREF_54) |
| KC346142 | Namibia - Khoe | [^54^](#_ENREF_54) |
| KC346143 | Namibia - Khoe | [^54^](#_ENREF_54) |
| KC346144 | Namibia - Khoe | [^54^](#_ENREF_54) |
| KC346145 | Namibia - Khoe | [^54^](#_ENREF_54) |
| KC346146 | Namibia - Khoe | [^54^](#_ENREF_54) |
| KC346147 | Namibia - Khoe | [^54^](#_ENREF_54) |
| KC346148 | Namibia - Khoe | [^54^](#_ENREF_54) |
| KC346149 | Namibia - Khoe | [^54^](#_ENREF_54) |
| KC346150 | Namibia - Khoe | [^54^](#_ENREF_54) |
| KC346151 | Namibia - Khoe | [^54^](#_ENREF_54) |
| KC346152 | Namibia - Khoe | [^54^](#_ENREF_54) |
| KC346153 | Namibia - Khoe | [^54^](#_ENREF_54) |
| KC346154 | Namibia - Khoe | [^54^](#_ENREF_54) |
| KC346155 | Namibia - Khoe | [^54^](#_ENREF_54) |
| KC346156 | Namibia - Khoe | [^54^](#_ENREF_54) |
| KC346157 | Namibia - Khoe | [^54^](#_ENREF_54) |
| KC346158 | Namibia - Khoe | [^54^](#_ENREF_54) |
| KC346159 | Namibia - Khoe | [^54^](#_ENREF_54) |
| KC346160 | Namibia - Khoe | [^54^](#_ENREF_54) |
| KC346161 | Namibia - Khoe | [^54^](#_ENREF_54) |
| KC346162 | Namibia - Khoe | [^54^](#_ENREF_54) |
| KC346163 | Namibia - Khoe | [^54^](#_ENREF_54) |
| KC346164 | Namibia - Khoe | [^54^](#_ENREF_54) |
| KC346165 | Namibia - Khoe | [^54^](#_ENREF_54) |
| KC346166 | Namibia - Khoe | [^54^](#_ENREF_54) |
| KC346167 | Namibia - Khoe | [^54^](#_ENREF_54) |
| KC346168 | Namibia - Khoe | [^54^](#_ENREF_54) |
| KC346169 | Namibia - Khoe | [^54^](#_ENREF_54) |
| KC346170 | Namibia - Khoe | [^54^](#_ENREF_54) |
| KC346171 | Namibia - Khoe | [^54^](#_ENREF_54) |
| KC346172 | Namibia - Khoe | [^54^](#_ENREF_54) |
| KC346173 | Zambia | [^54^](#_ENREF_54) |
| KC346174 | Namibia - Khoe | [^54^](#_ENREF_54) |
| KC346175 | Namibia - Khoe | [^54^](#_ENREF_54) |
| KC346176 | Namibia - Khoe | [^54^](#_ENREF_54) |
| KC346177 | Namibia - Khoe | [^54^](#_ENREF_54) |
| KC346178 | Namibia - Khoe | [^54^](#_ENREF_54) |
| KC346179 | Namibia - Khoe | [^54^](#_ENREF_54) |
| KC346180 | Namibia - Khoe | [^54^](#_ENREF_54) |
| KC346181 | Namibia - Khoe | [^54^](#_ENREF_54) |
| KC346182 | Namibia - Khoe | [^54^](#_ENREF_54) |
| KC346183 | Namibia - Khoe | [^54^](#_ENREF_54) |
| KC346184 | Namibia - Khoe | [^54^](#_ENREF_54) |
| KC346185 | Namibia - Khoe | [^54^](#_ENREF_54) |
| KC346186 | Namibia - Khoe | [^54^](#_ENREF_54) |
| KC346187 | Namibia - Khoe | [^54^](#_ENREF_54) |
| KC346188 | Namibia - Khoe | [^54^](#_ENREF_54) |
| KC346189 | Namibia - Khoe | [^54^](#_ENREF_54) |
| KC346190 | Namibia - Khoe | [^54^](#_ENREF_54) |
| KC346191 | Namibia - Khoe | [^54^](#_ENREF_54) |
| KC346192 | Namibia - Khoe | [^54^](#_ENREF_54) |
| KC346193 | Namibia - Khoe | [^54^](#_ENREF_54) |
| KC346194 | Namibia - Khoe | [^54^](#_ENREF_54) |
| KC346195 | Namibia - Khoe | [^54^](#_ENREF_54) |
| KC346196 | Namibia - Khoe | [^54^](#_ENREF_54) |
| KC346197 | Namibia - Khoe | [^54^](#_ENREF_54) |
| KC346198 | Namibia - Khoe | [^54^](#_ENREF_54) |
| KC346199 | Namibia - Khoe | [^54^](#_ENREF_54) |
| KC346200 | Namibia - Khoe | [^54^](#_ENREF_54) |
| KC346201 | Namibia - Khoe | [^54^](#_ENREF_54) |
| KC346202 | Namibia - Khoe | [^54^](#_ENREF_54) |
| KC346203 | Namibia - Khoe | [^54^](#_ENREF_54) |
| KC346204 | Namibia - Khoe | [^54^](#_ENREF_54) |
| KC346205 | Namibia - Khoe | [^54^](#_ENREF_54) |
| KC346206 | Namibia - Khoe | [^54^](#_ENREF_54) |
| KC346207 | Namibia - Khoe | [^54^](#_ENREF_54) |
| KC346208 | Namibia - Khoe | [^54^](#_ENREF_54) |
| KC346209 | Namibia - Khoe | [^54^](#_ENREF_54) |
| KC346210 | Namibia - Khoe | [^54^](#_ENREF_54) |
| KC346211 | Namibia - Khoe | [^54^](#_ENREF_54) |
| KC346212 | Namibia - Khoe | [^54^](#_ENREF_54) |
| KC346213 | Namibia - Khoe | [^54^](#_ENREF_54) |
| KC346214 | Namibia - Khoe | [^54^](#_ENREF_54) |
| KC346215 | Namibia - Khoe | [^54^](#_ENREF_54) |
| KC346216 | Namibia - Khoe | [^54^](#_ENREF_54) |
| KC346217 | Namibia - Khoe | [^54^](#_ENREF_54) |
| KC346218 | Namibia - Khoe | [^54^](#_ENREF_54) |
| KC346219 | Namibia - Khoe | [^54^](#_ENREF_54) |
| KC346220 | Namibia - Khoe | [^54^](#_ENREF_54) |
| KC346221 | Namibia - Khoe | [^54^](#_ENREF_54) |
| KC346222 | Namibia - Khoe | [^54^](#_ENREF_54) |
| KC346223 | Namibia - Khoe | [^54^](#_ENREF_54) |
| KC346224 | Namibia - Khoe | [^54^](#_ENREF_54) |
| KC346225 | Namibia - Khoe | [^54^](#_ENREF_54) |
| KC346226 | Namibia - Khoe | [^54^](#_ENREF_54) |
| KC346227 | Namibia - Khoe | [^54^](#_ENREF_54) |
| KC346228 | Namibia - Khoe | [^54^](#_ENREF_54) |
| KC346229 | Namibia - Khoe | [^54^](#_ENREF_54) |
| KC346230 | Namibia - Khoe | [^54^](#_ENREF_54) |
| KC346231 | Namibia - Khoe | [^54^](#_ENREF_54) |
| KC346232 | Namibia - Khoe | [^54^](#_ENREF_54) |
| KC346233 | Zambia | [^54^](#_ENREF_54) |
| KC346234 | Zambia | [^54^](#_ENREF_54) |
| KC346235 | Zambia | [^54^](#_ENREF_54) |
| KC346236 | Zambia | [^54^](#_ENREF_54) |
| KC346237 | Zambia | [^54^](#_ENREF_54) |
| KC346238 | Zambia | [^54^](#_ENREF_54) |
| KC346239 | Zambia | [^54^](#_ENREF_54) |
| KC346240 | Zambia | [^54^](#_ENREF_54) |
| KC346241 | Zambia | [^54^](#_ENREF_54) |
| KC346242 | Zambia | [^54^](#_ENREF_54) |
| KC346243 | Zambia | [^54^](#_ENREF_54) |
| KC346244 | Zambia | [^54^](#_ENREF_54) |
| KC346245 | Zambia | [^54^](#_ENREF_54) |
| KC346246 | Zambia | [^54^](#_ENREF_54) |
| KC346247 | Zambia | [^54^](#_ENREF_54) |
| KC346248 | Zambia | [^54^](#_ENREF_54) |
| KC533466 | South Africa | [^55^](#_ENREF_55) |
| KC533475 | South Africa: Mpumalanga Province | [^55^](#_ENREF_55) |
| KC533476 | South Africa | [^55^](#_ENREF_55) |
| KC533477 | South Africa | [^55^](#_ENREF_55) |
| KC533478 | South Africa | [^55^](#_ENREF_55) |
| KC533479 | South Africa | [^55^](#_ENREF_55) |
| KC533481 | South Africa | [^55^](#_ENREF_55) |
| KC533486 | South Africa | [^55^](#_ENREF_55) |
| KC533487 | South Africa | [^55^](#_ENREF_55) |
| KC533490 | South Africa | [^55^](#_ENREF_55) |
| KC533494 | South Africa | [^55^](#_ENREF_55) |
| KC533495 | South Africa | [^55^](#_ENREF_55) |
| KC533496 | South Africa | [^55^](#_ENREF_55) |
| KC533497 | South Africa | [^55^](#_ENREF_55) |
| KC533498 | South Africa | [^55^](#_ENREF_55) |
| KC533502 | South Africa | [^55^](#_ENREF_55) |
| KC533509 | South Africa | [^55^](#_ENREF_55) |
| KC533510 | South Africa | [^55^](#_ENREF_55) |
| KC533516 | South Africa | [^55^](#_ENREF_55) |
| KC622056 | Botswana | [^56^](#_ENREF_56) |
| KC622063 | Botswana - Tswana | [^56^](#_ENREF_56) |
| KC622064 | Botswana - Tswana | [^56^](#_ENREF_56) |
| KC622065 | Botswana - Tswana | [^56^](#_ENREF_56) |
| KC622067 | Botswana - Tswana | [^56^](#_ENREF_56) |
| KC622068 | Botswana - Tswana | [^56^](#_ENREF_56) |
| KC622077 | Botswana - Kgalagadi | [^56^](#_ENREF_56) |
| KC622078 | Botswana - Kalanga | [^56^](#_ENREF_56) |
| KC622100 | Namibia - Damara | [^56^](#_ENREF_56) |
| KC622104 | Namibia - Xo | [^56^](#_ENREF_56) |
| KC622106 | Botswana - Taa_West | [^56^](#_ENREF_56) |
| KC622107 | Botswana - Ani | [^56^](#_ENREF_56) |
| KC622112 | Botswana - Kgalagadi | [^56^](#_ENREF_56) |
| KC622114 | Botswana - Kgalagadi | [^56^](#_ENREF_56) |
| KC622115 | Botswana - Kgalagadi | [^56^](#_ENREF_56) |
| KC622117 | Botswana - Kgalagadi | [^56^](#_ENREF_56) |
| KC622121 | Namibia - Owambo | [^56^](#_ENREF_56) |
| KC622150 | Namibia - Nama | [^56^](#_ENREF_56) |
| KC622151 | Namibia - Nama | [^56^](#_ENREF_56) |
| KC622154 | Namibia - Kwamulonga Owambo | [^56^](#_ENREF_56) |
| KC622164 | Namibia - Xo | [^56^](#_ENREF_56) |
| KC622176 | Namibia - !Xun | [^56^](#_ENREF_56) |
| KC622187 | Namibia - Kwanyama | [^56^](#_ENREF_56) |
| KC622222 | Botswana - Buga | [^56^](#_ENREF_56) |
| KC622249 | Botswana - Tshwa | [^56^](#_ENREF_56) |
| KC622272 | Botswana - Tshwa | [^56^](#_ENREF_56) |
| KF672796 | Cameroon - Bulahay | [^57^](#_ENREF_57) |
| KF672797 | Mozambique | [^57^](#_ENREF_57) |
| KF672798 | Mozambique | [^57^](#_ENREF_57) |
| KF672799 | Mozambique | [^57^](#_ENREF_57) |
| KF672800 | Kenya | [^57^](#_ENREF_57) |
| KF672801 | Mozambique | [^57^](#_ENREF_57) |
| KF672802 | Mozambique | [^57^](#_ENREF_57) |
| KF672803 | Mozambique | [^57^](#_ENREF_57) |
| KF672804 | Mozambique | [^57^](#_ENREF_57) |
| KF672805 | Mozambique | [^57^](#_ENREF_57) |
| KF672806 | Mozambique | [^57^](#_ENREF_57) |
| KF672807 | Chad- Daza | [^57^](#_ENREF_57) |
| KF672808 | Mozambique | [^57^](#_ENREF_57) |
| KF672809 | Somalia | [^57^](#_ENREF_57) |
| KF672810 | Somalia | [^57^](#_ENREF_57) |
| KF672811 | Chad-Daza | [^57^](#_ENREF_57) |
| KF672812 | Ethiopia- Oromo | [^57^](#_ENREF_57) |
| KF672813 | Somalia | [^57^](#_ENREF_57) |
| KF672814 | Sudan | [^57^](#_ENREF_57) |
| KF672815 | Kenya- Turkana | [^57^](#_ENREF_57) |
| KF672816 | Mozambique | [^57^](#_ENREF_57) |
| KF672817 | Somalia | [^57^](#_ENREF_57) |
| KF672818 | Mozambique | [^57^](#_ENREF_57) |
| KF672819 | Mozambique | [^57^](#_ENREF_57) |
| KF672820 | Somalia | [^57^](#_ENREF_57) |
| KF672821 | Ethiopia | [^57^](#_ENREF_57) |
| KF672822 | S. Tomé Príncipe | [^57^](#_ENREF_57) |
| KF672823 | Somalia | [^57^](#_ENREF_57) |
| KF672824 | S. Tomé Príncipe | [^57^](#_ENREF_57) |
| KF672825 | Somalia | [^57^](#_ENREF_57) |
| KF672826 | S. Tomé Príncipe | [^57^](#_ENREF_57) |
| KF672827 | Cameroon- Kotoco | [^57^](#_ENREF_57) |
| KF672828 | Nubia | [^57^](#_ENREF_57) |
| KF672829 | Chad | [^57^](#_ENREF_57) |
| KF672830 | Ethiopia- Oromo | [^57^](#_ENREF_57) |
| KF672831 | Niger- Zinder | [^57^](#_ENREF_57) |
| KF672832 | Mozambique | [^57^](#_ENREF_57) |
| KF672833 | Cameroon | [^57^](#_ENREF_57) |
| KF672834 | S. Tomé Príncipe | [^57^](#_ENREF_57) |
| KF672835 | Somalia | [^57^](#_ENREF_57) |
| KF672836 | Sudan - Arab | [^57^](#_ENREF_57) |
| KF672837 | Sudan - Nubian | [^57^](#_ENREF_57) |
| KJ185394 | Zambia - Aushi | [^58^](#_ENREF_58) |
| KJ185396 | Zambia - Kwamulonga Bemba | [^58^](#_ENREF_58) |
| KJ185397 | Zambia - Bemba | [^58^](#_ENREF_58) |
| KJ185398 | Zambia - Bemba | [^58^](#_ENREF_58) |
| KJ185399 | Zambia | [^58^](#_ENREF_58) |
| KJ185400 | Zambia - Bemba | [^58^](#_ENREF_58) |
| KJ185408 | Zambia - Bisa | [^58^](#_ENREF_58) |
| KJ185423 | Zambia - Luchazi | [^58^](#_ENREF_58) |
| KJ185424 | Zambia - Luchazi | [^58^](#_ENREF_58) |
| KJ185425 | Zambia - Luchazi | [^58^](#_ENREF_58) |
| KJ185430 | Zambia | [^58^](#_ENREF_58) |
| KJ185431 | Zambia - Luvale | [^58^](#_ENREF_58) |
| KJ185432 | Zambia - Luvale | [^58^](#_ENREF_58) |
| KJ185433 | Zambia - Luvale | [^58^](#_ENREF_58) |
| KJ185457 | Zambia - Sala | [^58^](#_ENREF_58) |
| KJ185458 | Zambia - Tokaleya | [^58^](#_ENREF_58) |
| KJ185461 | Zambia - Tonga | [^58^](#_ENREF_58) |
| KJ185462 | Zambia - Tonga | [^58^](#_ENREF_58) |
| KJ185463 | Zambia | [^58^](#_ENREF_58) |
| KJ185476 | Angola - Ganguela | [^58^](#_ENREF_58) |
| KJ185477 | Angola | [^58^](#_ENREF_58) |
| KJ185478 | Angola | [^58^](#_ENREF_58) |
| KJ185479 | Angola | [^58^](#_ENREF_58) |
| KJ185480 | Angola - Ganguela | [^58^](#_ENREF_58) |
| KJ185494 | Angola - Kuvale | [^58^](#_ENREF_58) |
| KJ185495 | Angola - Kuvale | [^58^](#_ENREF_58) |
| KJ185496 | Angola - Kuvale | [^58^](#_ENREF_58) |
| KJ185497 | Angola - Kuvale | [^58^](#_ENREF_58) |
| KJ185498 | Angola - Kuvale | [^58^](#_ENREF_58) |
| KJ185499 | Angola - Kuvale | [^58^](#_ENREF_58) |
| KJ185500 | Angola - Kuvale | [^58^](#_ENREF_58) |
| KJ185501 | Angola - Kuvale | [^58^](#_ENREF_58) |
| KJ185502 | Angola - Kuvale | [^58^](#_ENREF_58) |
| KJ185503 | Angola - Kuvale | [^58^](#_ENREF_58) |
| KJ185504 | Angola - Kuvale | [^58^](#_ENREF_58) |
| KJ185505 | Angola - Kuvale | [^58^](#_ENREF_58) |
| KJ185506 | Angola - Kuvale | [^58^](#_ENREF_58) |
| KJ185507 | Angola - Kuvale | [^58^](#_ENREF_58) |
| KJ185508 | Angola - Kuvale | [^58^](#_ENREF_58) |
| KJ185509 | Angola - Kuvale | [^58^](#_ENREF_58) |
| KJ185510 | Angola - Kuvale | [^58^](#_ENREF_58) |
| KJ185540 | Zambia - Lozi | [^58^](#_ENREF_58) |
| KJ185541 | Zambia - Lozi | [^58^](#_ENREF_58) |
| KJ185542 | Zambia - Lozi | [^58^](#_ENREF_58) |
| KJ185543 | Zambia - Lozi | [^58^](#_ENREF_58) |
| KJ185544 | Zambia - Lozi | [^58^](#_ENREF_58) |
| KJ185545 | Zambia - Lozi | [^58^](#_ENREF_58) |
| KJ185546 | Zambia - Lozi | [^58^](#_ENREF_58) |
| KJ185547 | Zambia - Lozi | [^58^](#_ENREF_58) |
| KJ185548 | Zambia - Lozi | [^58^](#_ENREF_58) |
| KJ185549 | Zambia - Lozi | [^58^](#_ENREF_58) |
| KJ185550 | Zambia - Lozi | [^58^](#_ENREF_58) |
| KJ185551 | Zambia - Lozi | [^58^](#_ENREF_58) |
| KJ185552 | Zambia - Lozi | [^58^](#_ENREF_58) |
| KJ185553 | Zambia - Lozi | [^58^](#_ENREF_58) |
| KJ185554 | Zambia - Lozi | [^58^](#_ENREF_58) |
| KJ185555 | Zambia - Lozi | [^58^](#_ENREF_58) |
| KJ185556 | Zambia - Lozi | [^58^](#_ENREF_58) |
| KJ185557 | Zambia - Tonga | [^58^](#_ENREF_58) |
| KJ185653 | Zambia - Mbunda | [^58^](#_ENREF_58) |
| KJ185654 | Zambia - Mbunda | [^58^](#_ENREF_58) |
| KJ185655 | Zambia - Mbunda | [^58^](#_ENREF_58) |
| KJ185656 | Zambia | [^58^](#_ENREF_58) |
| KJ185657 | Zambia - Mbunda | [^58^](#_ENREF_58) |
| KJ185658 | Zambia - Mbunda | [^58^](#_ENREF_58) |
| KJ185659 | Zambia - Mbunda | [^58^](#_ENREF_58) |
| KJ185660 | Zambia - Mbunda | [^58^](#_ENREF_58) |
| KJ185717 | Zambia - Nkoya | [^58^](#_ENREF_58) |
| KJ185718 | Zambia - Nkoya | [^58^](#_ENREF_58) |
| KJ185719 | Zambia - Nkoya | [^58^](#_ENREF_58) |
| KJ185720 | Zambia - Nkoya | [^58^](#_ENREF_58) |
| KJ185721 | Zambia - Nkoya | [^58^](#_ENREF_58) |
| KJ185722 | Zambia - Kwamulonga Nkoya | [^58^](#_ENREF_58) |
| KJ185749 | Angola - Nyaneka | [^58^](#_ENREF_58) |
| KJ185750 | Angola - Nyaneka | [^58^](#_ENREF_58) |
| KJ185751 | Angola - Nyaneka | [^58^](#_ENREF_58) |
| KJ185752 | Angola - Nyaneka | [^58^](#_ENREF_58) |
| KJ185753 | Angola - Nyaneka | [^58^](#_ENREF_58) |
| KJ185754 | Angola - Nyaneka | [^58^](#_ENREF_58) |
| KJ185755 | Angola - Nyaneka | [^58^](#_ENREF_58) |
| KJ185756 | Angola - Nyaneka | [^58^](#_ENREF_58) |
| KJ185757 | Angola - Nyaneka | [^58^](#_ENREF_58) |
| KJ185758 | Angola - Nyaneka | [^58^](#_ENREF_58) |
| KJ185759 | Angola - Nyaneka | [^58^](#_ENREF_58) |
| KJ185802 | Angola - Ovimbundu | [^58^](#_ENREF_58) |
| KJ185803 | Angola - Ovimbundu | [^58^](#_ENREF_58) |
| KJ185804 | Angola - Ovimbundu | [^58^](#_ENREF_58) |
| KJ185805 | Angola - Ovimbundu | [^58^](#_ENREF_58) |
| KJ185806 | Angola - Ovimbundu | [^58^](#_ENREF_58) |
| KJ185858 | Zambia - Shanjo | [^58^](#_ENREF_58) |
| KJ185862 | Zambia - Tswana | [^58^](#_ENREF_58) |
| KJ185863 | Zambia - Tswana | [^58^](#_ENREF_58) |
| KJ185865 | Zambia - Kwamulonga | [^58^](#_ENREF_58) |
| KJ185881 | Zambia - Kwamulonga Kwangwa | [^58^](#_ENREF_58) |
| KJ185882 | Zambia - Kwangwa | [^58^](#_ENREF_58) |
| KJ185883 | Zambia - Kwamulonga Kwangwa | [^58^](#_ENREF_58) |
| KJ185884 | Zambia - Kwangwa | [^58^](#_ENREF_58) |
| KJ185926 | Zambia - Makoma | [^58^](#_ENREF_58) |
| KJ185946 | Zambia - Nyengo | [^58^](#_ENREF_58) |
| KJ185947 | Zambia - Nyengo | [^58^](#_ENREF_58) |
| KJ185966 | Zambia - Yauma | [^58^](#_ENREF_58) |
| KJ185967 | Zambia - Shona | [^58^](#_ENREF_58) |
| KJ185968 | Zambia - Shona | [^58^](#_ENREF_58) |
| KJ185970 | Zambia - Chewa | [^58^](#_ENREF_58) |
| KJ185972 | Zambia - Kaonde | [^58^](#_ENREF_58) |
| KJ185977 | Zambia - Lunda | [^58^](#_ENREF_58) |
| KJ185978 | Zambia - Lunda | [^58^](#_ENREF_58) |
| KJ185979 | Zambia - Lunda | [^58^](#_ENREF_58) |
| KJ185990 | Zambia | [^58^](#_ENREF_58) |
| KJ185995 | Zambia - Ngoni | [^58^](#_ENREF_58) |
| KJ186001 | Zambia - Tebele | [^58^](#_ENREF_58) |
| KJ186004 | Zambia - Yeyi | [^58^](#_ENREF_58) |
| KJ186005 | Zambia - Yeyi | [^58^](#_ENREF_58) |
| KJ186008 | Angola - Nyaneka | [^58^](#_ENREF_58) |
| KJ186009 | Angola - Ovimbundu | [^58^](#_ENREF_58) |
| KJ445738 | Pakistan | HGDP database^b^ |
| KJ445739 | South_Africa - Bantu | HGDP database^b^ |
| KJ445740 | Namibia - San | HGDP database^b^ |
| KJ445741 | Namibia - San | HGDP database^b^ |
| KJ445742 | Namibia - San | HGDP database^b^ |
| KJ445743 | S. Africa - Bantu | HGDP database^b^ |
| KJ445744 | Namibia - San | HGDP database^b^ |
| KJ445745 | Namibia - San | HGDP database^b^ |
| KJ445746 | Namibia- San | HGDP database^b^ |
| KJ445747 | Pakistan | HGDP database^b^ |
| KJ445748 | Israel-Bedouin | HGDP database^b^ |
| KJ445749 | Israel-Bedouin | HGDP database^b^ |
| KJ445750 | Kenya - Bantu | HGDP database^b^ |
| KJ445751 | Israel-Bedouin | HGDP database^b^ |
| KJ445752 | Kenya - Bantu | HGDP database^b^ |
| KJ445753 | Congo - Mbuti | HGDP database^b^ |
| KJ445754 | Congo - Mbuti | HGDP database^b^ |
| KJ445755 | Congo - Mbuti | HGDP database^b^ |
| KJ445756 | Congo - Mbuti | HGDP database^b^ |
| KJ445757 | CAR - Pygmy | HGDP database^b^ |
| KJ445758 | CAR - Pygmy | HGDP database^b^ |
| KJ445759 | CAR - Pygmy | HGDP database^b^ |
| KJ445760 | CAR - Pygmy | HGDP database^b^ |
| KJ445761 | Kenya | HGDP database^b^ |
| KJ445762 | Pakistan - Sindhi | HGDP database^b^ |
| KJ445763 | South Africa | HGDP database^b^ |
| KJ669103 | Namibia - Nama | [^59^](#_ENREF_59) |
| KJ669104 | Namibia - Khwe | [^59^](#_ENREF_59) |
| KJ669105 | Namibia - Khwe | [^59^](#_ENREF_59) |
| KJ669106 | Namibia - !Xun | [^59^](#_ENREF_59) |
| KJ669107 | Namibia - Ju/'hoan | [^59^](#_ENREF_59) |
| KJ669108 | Namibia - Ju/'hoan | [^59^](#_ENREF_59) |
| KJ669109 | Namibia - Ju/'hoan | [^59^](#_ENREF_59) |
| KJ669110 | Namibia - !Xun | [^59^](#_ENREF_59) |
| KJ669111 | Angola | [^59^](#_ENREF_59) |
| KJ669112 | Namibia - !Xun | [^59^](#_ENREF_59) |
| KJ669113 | Zimbabwe - Shona | [^59^](#_ENREF_59) |
| KJ669114 | South Africa - Xhosa | [^59^](#_ENREF_59) |
| KJ669115 | Namibia - Tjimba | [^59^](#_ENREF_59) |
| KJ669116 | Namibia - Tau | [^59^](#_ENREF_59) |
| KJ669117 | Namibia - Kwambi | [^59^](#_ENREF_59) |
| KJ669118 | Namibia - Owambo | [^59^](#_ENREF_59) |
| KJ669119 | Namibia - Caprivian | [^59^](#_ENREF_59) |
| KJ669120 | Zimbabwe - Shona | [^59^](#_ENREF_59) |
| KJ669121 | Namibia - Baster | [^59^](#_ENREF_59) |
| KJ669122 | Namibia - Kwambi | [^59^](#_ENREF_59) |
| KJ669123 | Namibia - Ndonga | [^59^](#_ENREF_59) |
| KJ669124 | Namibia - !Xun | [^59^](#_ENREF_59) |
| KJ669125 | Namibia - Ju/'hoan | [^59^](#_ENREF_59) |
| KJ669126 | Namibia - Ju/'hoan | [^59^](#_ENREF_59) |
| KJ669127 | Namibia - Ju/'hoan | [^59^](#_ENREF_59) |
| KJ669128 | Namibia - Hai//om | [^59^](#_ENREF_59) |
| KJ669129 | Namibia - Hai//om | [^59^](#_ENREF_59) |
| KJ669130 | Namibia - Hai//om | [^59^](#_ENREF_59) |
| KJ669131 | Namibia- Hai/Khau-na | [^59^](#_ENREF_59) |
| KJ669132 | Namibia - Nama | [^59^](#_ENREF_59) |
| KJ669133 | Namibia - Damara | [^59^](#_ENREF_59) |
| KJ669134 | Namibia- Tuu | [^59^](#_ENREF_59) |
| KJ669135 | South Africa - Venda | [^59^](#_ENREF_59) |
| KJ669137 | South Africa - Coloured | [^59^](#_ENREF_59) |
| KJ669138 | South Africa - Xhosa | [^59^](#_ENREF_59) |
| KJ669139 | Namibia - !Xun | [^59^](#_ENREF_59) |
| KJ669140 | Namibia - Herero | [^59^](#_ENREF_59) |
| KJ669141 | South Africa - Coloured | [^59^](#_ENREF_59) |
| KJ669142 | Namibia - Nama | [^59^](#_ENREF_59) |
| KJ669143 | Namibia - Nama | [^59^](#_ENREF_59) |
| KJ669144 | South Africa - Zulu | [^59^](#_ENREF_59) |
| KJ669145 | South Africa - Tswana | [^59^](#_ENREF_59) |
| KJ669146 | Namibia - Naro | [^59^](#_ENREF_59) |
| KJ669147 | Namibia - Ju/'hoan | [^59^](#_ENREF_59) |
| KJ669148 | Namibia - Ju/'hoan | [^59^](#_ENREF_59) |
| KJ669149 | Namibia | [^59^](#_ENREF_59) |
| KJ669150 | South Africa - Tsonga | [^59^](#_ENREF_59) |
| KJ669151 | Namibia - !Xun | [^59^](#_ENREF_59) |
| KJ669152 | Namibia | [^59^](#_ENREF_59) |
| KJ669153 | Namibia - Damara | [^59^](#_ENREF_59) |
| KJ669154 | South Africa - Baster | [^59^](#_ENREF_59) |
| KJ669155 | Namibia - Baster | [^59^](#_ENREF_59) |
| KJ669156 | South Africa - Pedi | [^59^](#_ENREF_59) |
| KJ669157 | South Africa - Pedi | [^59^](#_ENREF_59) |
| KJ669158 | Coastal Khoe-San remains | [^59^](#_ENREF_59) |
| KJ669159 | Namibia - Hai//om | [^59^](#_ENREF_59) |
| KJ669160 | Namibia - !Xun | [^59^](#_ENREF_59) |
| KJ669161 | Namibia - Hai//om | [^59^](#_ENREF_59) |
| KJ669162 | South Africa - Xhosa | [^59^](#_ENREF_59) |
| KJ669163 | Namibia - Damara | [^59^](#_ENREF_59) |
| KJ669164 | Namibia - Nama | [^59^](#_ENREF_59) |
| KJ669165 | Namibia - Damara | [^59^](#_ENREF_59) |
| KJ669166 | South Africa - Sotho | [^59^](#_ENREF_59) |
| KJ669167 | Namibia - Hai//om | [^59^](#_ENREF_59) |
| KJ669168 | Namibia - Baster | [^59^](#_ENREF_59) |
| KJ669169 | South Africa - Tswana | [^59^](#_ENREF_59) |
| KJ669170 | South Africa - Sotho | [^59^](#_ENREF_59) |
| KJ669171 | South Africa - Xhosa | [^59^](#_ENREF_59) |
| KJ669172 | Namibia - Owambo | [^59^](#_ENREF_59) |
| KJ669173 | Namibia - Baster | [^59^](#_ENREF_59) |
| KJ669174 | South Africa - Venda | [^59^](#_ENREF_59) |
| KJ669175 | Namibia - Hai//om | [^59^](#_ENREF_59) |
| KJ669176 | South Africa | [^59^](#_ENREF_59) |
| KJ669177 | South Africa - coloured | [^59^](#_ENREF_59) |
| KJ669178 | Namibia - Damara | [^59^](#_ENREF_59) |
| KJ669179 | Namibia - Baster | [^59^](#_ENREF_59) |
| KJ669180 | South Africa - Zulu | [^59^](#_ENREF_59) |
| KM101580 | USA | [^60^](#_ENREF_60) |
| KM101598 | USA | [^60^](#_ENREF_60) |
| KM101644 | USA | [^60^](#_ENREF_60) |
| KM101662 | USA | [^60^](#_ENREF_60) |
| KM101678 | USA | [^60^](#_ENREF_60) |
| KM102081 | USA | [^60^](#_ENREF_60) |
| KM986519 | Yemen | [^61^](#_ENREF_61) |
| KM986528 | Uganda | [^61^](#_ENREF_61) |
| KM986532 | Yemen | [^61^](#_ENREF_61) |
| KM986555 | Yemen | [^61^](#_ENREF_61) |
| KM986563 | Yemen | [^61^](#_ENREF_61) |
| KM986565 | Yemen | [^61^](#_ENREF_61) |
| KM986569 | Yemen | [^61^](#_ENREF_61) |
| KM986571 | Yemen | [^61^](#_ENREF_61) |
| KM986580 | Yemen | [^61^](#_ENREF_61) |
| KM986599 | Yemen | [^61^](#_ENREF_61) |
| KM986609 | Yemen | [^61^](#_ENREF_61) |
| KM986624 | Yemen | [^61^](#_ENREF_61) |
| KP635238 | Bolivia | [^62^](#_ENREF_62) |
| KP635239 | Bolivia | [^62^](#_ENREF_62) |
| KP635243 | Bolivia - Yungas | [^62^](#_ENREF_62) |
| NA18510 | Nigeria - Yoruba | [^63^](#_ENREF_63) |
| NA18861 | Nigeria - Yoruba | 1K GP database^a^ |
| NA18876 | Nigeria - Yoruba | 1K GP database^a^ |
| NA18877 | Nigeria - Yoruba | 1K GP database^a^ |
| NA19027 | Kenya - Luhya | 1K GP database^a^ |
| NA19031 | Kenya - Luhya | 1K GP database^a^ |
| NA19039 | Kenya - Luhya | 1K GP database^a^ |
| NA19042 | Kenya - Luhya | 1K GP database^a^ |
| NA19137 | Nigeria - Yoruba | 1K GP database^a^ |
| NA19156 | Nigeria - Yoruba | 1K GP database^a^ |
| NA19216 | Nigeria - Yoruba | 1K GP database^a^ |
| NA19311 | Kenya - Luhya | 1K GP database^a^ |
| NA19312 | Kenya - Luhya | 1K GP database^a^ |
| NA19327 | Kenya - Luhya | 1K GP database^a^ |
| NA19328 | Kenya - Luhya | 1K GP database^a^ |
| NA19350 | Kenya - Luhya | 1K GP database^a^ |
| NA19379 | Kenya - Luhya | 1K GP database^a^ |
| NA19382 | Kenya - Luhya | 1K GP database^a^ |
| NA19402 | Kenya - Luhya | 1K GP database^a^ |
| NA19430 | Kenya - Luhya | 1K GP database^a^ |
| NA19440 | Kenya - Luhya | 1K GP database^a^ |
| NA19448 | Kenya - Luhya | 1K GP database^a^ |
| NA19449 | Kenya - Luhya | 1K GP database^a^ |
| NA19454 | Kenya - Luhya | 1K GP database^a^ |
| NA19466 | Kenya - Luhya | 1K GP database^a^ |
| NA19467 | Kenya - Luhya | 1K GP database^a^ |
| NA19474 | Kenya - Luhya | 1K GP database^a^ |
| NA19703 | USA | 1K GP database^a^ |
| NA19713 | USA | 1K GP database^a^ |
| NA19904 | USA | 1K GP database^a^ |
| NA19985 | USA | 1K GP database^a^ |
| NA20334 | USA | 1K GP database^a^ |
| NA20336 | USA | 1K GP database^a^ |
| NA20355 | USA | 1K GP database^a^ |

^a^ 1000 Genomes Project database. <http://www.internationalgenome.org/>

^b^ Human Genome Diversity Project. <http://www.hagsc.org/hgdp/>

**Table S4.** Modern mtDNA haplogroup L1’6 sequences used in this study.

| **Accession number** | **Reference** |
| --- | --- |
| AF346987 | [^37^](#_ENREF_37) |
| AF346995 | [^37^](#_ENREF_37) |
| AY195785 | [^40^](#_ENREF_40) |
| DQ282507 | [^42^](#_ENREF_42) |
| DQ304924 | [^42^](#_ENREF_42) |
| DQ304925 | [^42^](#_ENREF_42) |
| DQ304926 | [^42^](#_ENREF_42) |
| DQ304928 | [^42^](#_ENREF_42) |
| DQ304945 | [^42^](#_ENREF_42) |
| DQ304946 | [^42^](#_ENREF_42) |
| DQ304949 | [^42^](#_ENREF_42) |
| DQ304954 | [^42^](#_ENREF_42) |
| DQ304985 | [^42^](#_ENREF_42) |
| DQ305010 | [^42^](#_ENREF_42) |
| DQ305018 | [^42^](#_ENREF_42) |
| DQ341061 | [^43^](#_ENREF_43) |
| DQ341063 | [^43^](#_ENREF_43) |
| DQ341064 | [^43^](#_ENREF_43) |
| DQ341065 | [^43^](#_ENREF_43) |
| DQ341069 | [^43^](#_ENREF_43) |
| DQ341074 | [^43^](#_ENREF_43) |
| DQ341075 | [^43^](#_ENREF_43) |
| DQ341076 | [^43^](#_ENREF_43) |
| DQ341078 | [^43^](#_ENREF_43) |
| DQ341080 | [^43^](#_ENREF_43) |
| DQ341081 | [^43^](#_ENREF_43) |
| EF556173 | [^45^](#_ENREF_45) |
| EU092660 | [^46^](#_ENREF_46) |
| EU092661 | [^46^](#_ENREF_46) |
| EU092671 | [^46^](#_ENREF_46) |
| EU092676 | [^46^](#_ENREF_46) |
| EU092678 | [^46^](#_ENREF_46) |
| EU092686 | [^46^](#_ENREF_46) |
| EU092693 | [^46^](#_ENREF_46) |
| EU092695 | [^46^](#_ENREF_46) |
| EU092697 | [^46^](#_ENREF_46) |
| EU092698 | [^46^](#_ENREF_46) |
| EU092699 | [^46^](#_ENREF_46) |
| EU092703 | [^46^](#_ENREF_46) |
| EU092709 | [^46^](#_ENREF_46) |
| EU092712 | [^46^](#_ENREF_46) |
| EU092715 | [^46^](#_ENREF_46) |
| EU092717 | [^46^](#_ENREF_46) |
| EU092724 | [^46^](#_ENREF_46) |
| EU092734 | [^46^](#_ENREF_46) |
| EU092736 | [^46^](#_ENREF_46) |
| EU092740 | [^46^](#_ENREF_46) |
| EU092747 | [^46^](#_ENREF_46) |
| EU092748 | [^46^](#_ENREF_46) |
| EU092750 | [^46^](#_ENREF_46) |
| EU092752 | [^46^](#_ENREF_46) |
| EU092766 | [^46^](#_ENREF_46) |
| EU092768 | [^46^](#_ENREF_46) |
| EU092770 | [^46^](#_ENREF_46) |
| EU092773 | [^46^](#_ENREF_46) |
| EU092774 | [^46^](#_ENREF_46) |
| EU092776 | [^46^](#_ENREF_46) |
| EU092781 | [^46^](#_ENREF_46) |
| EU092784 | [^46^](#_ENREF_46) |
| EU092802 | [^46^](#_ENREF_46) |
| EU092813 | [^46^](#_ENREF_46) |
| EU092817 | [^46^](#_ENREF_46) |
| EU092818 | [^46^](#_ENREF_46) |
| EU092822 | [^46^](#_ENREF_46) |
| EU092824 | [^46^](#_ENREF_46) |
| EU092838 | [^46^](#_ENREF_46) |
| EU092848 | [^46^](#_ENREF_46) |
| EU092851 | [^46^](#_ENREF_46) |
| EU092877 | [^46^](#_ENREF_46) |
| EU092885 | [^46^](#_ENREF_46) |
| EU092886 | [^46^](#_ENREF_46) |
| EU092888 | [^46^](#_ENREF_46) |
| EU092890 | [^46^](#_ENREF_46) |
| EU092891 | [^46^](#_ENREF_46) |
| EU092898 | [^46^](#_ENREF_46) |
| EU092902 | [^46^](#_ENREF_46) |
| EU092915 | [^46^](#_ENREF_46) |
| EU092916 | [^46^](#_ENREF_46) |
| EU092923 | [^46^](#_ENREF_46) |
| EU092934 | [^46^](#_ENREF_46) |
| EU092935 | [^46^](#_ENREF_46) |
| EU092941 | [^46^](#_ENREF_46) |
| EU092942 | [^46^](#_ENREF_46) |
| EU092943 | [^46^](#_ENREF_46) |
| EU092944 | [^46^](#_ENREF_46) |
| EU092949 | [^46^](#_ENREF_46) |
| EU273484 | [^64^](#_ENREF_64) |
| EU273489 | [^64^](#_ENREF_64) |
| EU273491 | [^64^](#_ENREF_64) |
| EU273493 | [^64^](#_ENREF_64) |
| EU273499 | [^64^](#_ENREF_64) |
| EU273501 | [^64^](#_ENREF_64) |
| EU597500 | [^47^](#_ENREF_47) |
| EU597570 | [^47^](#_ENREF_47) |
| EU935440 | [^48^](#_ENREF_48) |
| FJ460520 | [^65^](#_ENREF_65) |
| FJ460531 | [^65^](#_ENREF_65) |
| FJ625848 | [^66^](#_ENREF_66) |
| FJ625856 | [^66^](#_ENREF_66) |
| GU455418 | [^67^](#_ENREF_67) |
| HM596745 | [Family](https://www.ncbi.nlm.nih.gov/nuccore/HM596745) Tree |
| HM771114 | [^50^](#_ENREF_50) |
| HM771117 | [^50^](#_ENREF_50) |
| HM771136 | [^50^](#_ENREF_50) |
| HM771162 | [^50^](#_ENREF_50) |
| HM771166 | [^50^](#_ENREF_50) |
| HM771171 | [^50^](#_ENREF_50) |
| HM771178 | [^50^](#_ENREF_50) |
| HM771184 | [^50^](#_ENREF_50) |
| HM771203 | [^50^](#_ENREF_50) |
| HM771204 | [^50^](#_ENREF_50) |
| HM771206 | [^50^](#_ENREF_50) |
| HM771211 | [^50^](#_ENREF_50) |
| HM771220 | [^50^](#_ENREF_50) |
| HM771223 | [^50^](#_ENREF_50) |
| HM771226 | [^50^](#_ENREF_50) |
| HM771233 | [^50^](#_ENREF_50) |
| HQ425328 | Family Tree |
| HQ425645 | Family Tree |
| JN655774 | [^68^](#_ENREF_68) |
| JN655776 | [^68^](#_ENREF_68) |
| JN655778 | [^68^](#_ENREF_68) |
| JN655780 | [^68^](#_ENREF_68) |
| JN655784 | [^68^](#_ENREF_68) |
| JN655785 | [^68^](#_ENREF_68) |
| JN655786 | [^68^](#_ENREF_68) |
| JN655787 | [^68^](#_ENREF_68) |
| JN655788 | [^68^](#_ENREF_68) |
| JN655794 | [^68^](#_ENREF_68) |
| JN655797 | [^68^](#_ENREF_68) |
| JN655798 | [^68^](#_ENREF_68) |
| JN655803 | [^68^](#_ENREF_68) |
| JN655812 | [^68^](#_ENREF_68) |
| JN655813 | [^68^](#_ENREF_68) |
| JN655815 | [^68^](#_ENREF_68) |
| JN655825 | [^68^](#_ENREF_68) |
| JN655830 | [^68^](#_ENREF_68) |
| JN655837 | [^68^](#_ENREF_68) |
| JQ044795 | [^51^](#_ENREF_51) |
| JQ044797 | [^51^](#_ENREF_51) |
| JQ044810 | [^51^](#_ENREF_51) |
| JQ044811 | [^51^](#_ENREF_51) |
| JQ044816 | [^51^](#_ENREF_51) |
| JQ044829 | [^51^](#_ENREF_51) |
| JQ044831 | [^51^](#_ENREF_51) |
| JQ044834 | [^51^](#_ENREF_51) |
| JQ044836 | [^51^](#_ENREF_51) |
| JQ044843 | [^51^](#_ENREF_51) |
| JQ044846 | [^51^](#_ENREF_51) |
| JQ044847 | [^51^](#_ENREF_51) |
| JQ044858 | [^51^](#_ENREF_51) |
| JQ044866 | [^51^](#_ENREF_51) |
| JQ044871 | [^51^](#_ENREF_51) |
| JQ044878 | [^51^](#_ENREF_51) |
| JQ044882 | [^51^](#_ENREF_51) |
| JQ044907 | [^51^](#_ENREF_51) |
| JQ044910 | [^51^](#_ENREF_51) |
| JQ044914 | [^51^](#_ENREF_51) |
| JQ044922 | [^51^](#_ENREF_51) |
| JQ044936 | [^51^](#_ENREF_51) |
| JQ044939 | [^51^](#_ENREF_51) |
| JQ044941 | [^51^](#_ENREF_51) |
| JQ044959 | [^51^](#_ENREF_51) |
| JQ044975 | [^51^](#_ENREF_51) |
| JQ045002 | [^51^](#_ENREF_51) |
| JQ045008 | [^51^](#_ENREF_51) |
| JQ045026 | [^51^](#_ENREF_51) |
| JQ045029 | [^51^](#_ENREF_51) |
| JQ045030 | [^51^](#_ENREF_51) |
| JQ045038 | [^51^](#_ENREF_51) |
| JQ045043 | [^51^](#_ENREF_51) |
| JQ045062 | [^51^](#_ENREF_51) |
| JQ045070 | [^51^](#_ENREF_51) |
| JQ045074 | [^51^](#_ENREF_51) |
| JQ045080 | [^51^](#_ENREF_51) |
| JQ045084 | [^51^](#_ENREF_51) |
| JQ045092 | [^51^](#_ENREF_51) |
| JQ045101 | [^51^](#_ENREF_51) |
| JQ045111 | [^51^](#_ENREF_51) |
| JQ701901 | [^52^](#_ENREF_52) |
| JQ701954 | [^52^](#_ENREF_52) |
| JQ702441 | [^52^](#_ENREF_52) |
| JQ702617 | [^52^](#_ENREF_52) |
| JQ702626 | [^52^](#_ENREF_52) |
| JQ702659 | [^52^](#_ENREF_52) |
| JQ703773 | [^52^](#_ENREF_52) |
| JQ703986 | [^52^](#_ENREF_52) |
| JQ704919 | [^52^](#_ENREF_52) |
| JQ705275 | [^52^](#_ENREF_52) |
| JQ705310 | [^52^](#_ENREF_52) |
| JQ705521 | [^52^](#_ENREF_52) |
| JQ705626 | [^52^](#_ENREF_52) |
| JQ705650 | [^52^](#_ENREF_52) |

**Table S5.** Parameters used in the coalescent simulations

| **Parameter** | **Description** | **Notes** |
| --- | --- | --- |
| **N0** | 10600 |  |
| **SegSize** | 16568 |  |
| **theta** | 2N0*Mut*SegSize | Mut is the neutral mutation rate per site per generation |
| **NKhoesan150ka** | 10600 | Khoe-San effective population size (150kyrs) |
| **NKhoesan75ka** | 10600 | Khoe-San effective population size (75kyrs) |
| **NKhoesan60ka** | 14100 | Khoe-San effective population size (60kyrs) |
| **NKhoesan0ka** | 25880 | Khoe-San effective population size (now) |
| **NEastern150ka** | 3950 | Eastern effective population size (150kyrs) |
| **NEastern75ka** | 5500 | Eastern effective population size (75kyrs) |
| **NEastern60ka** | 13870 | Eastern effective population size (60kyrs) |
| **NEastern0ka** | 138500 | Eastern effective population size (now) |
| **T0** | 0 |  |
| **T1** | 60 ka |  |
| **T2** | 75 ka |  |
| **T3** | 150 ka |  |
| **Tm1 & Tm2** | variable | Times for migration periods. Migration duration = Tm2 - Tm1 |
| **Mig** | variable | Number of mitogenomes which migrated in each generation |
| **Mut** | variable | neutral mutation rate per site per generation |

**Table S6.** Population and sample sizes used in the various genome-wide analyses in this study.

| **Population** | **Sample size (n)** | | | | | |
| --- | --- | --- | --- | --- | --- | --- |
|  | **Initial Dataset –**  **preliminary**  **analysis** | **Principal**  **Component**  **Analysis**  **(PCA)** | **ADMIXTURE/**  **sNMF/**  **preliminary**  **TreeMix** | **Final**  **TreeMix** | **References** |  |
| Arab | 16 | 16 | 16 | 15 | [^69^](#_ENREF_69) |  |
| Coloured_Colesberg | 20 | - | - | - | [^70^](#_ENREF_70) |  |
| Coloured_Wellington | 20 | - | - | - | [^70^](#_ENREF_70) |  |
| Daza | 18 | 18 | 18 | - | [^69^](#_ENREF_69) |  |
| Esan | 33 | 33 | 33 | 33 | [^71^](#_ENREF_71) |  |
| Fulani | 13 | - | - | - | [^69^](#_ENREF_69) |  |
| Gambian | 39 | 39 | 39 | 36 | [^71^](#_ENREF_71) |  |
| GuiGhanaKgal | 15 | 15 | 15 | - | [^70^](#_ENREF_70) |  |
| Gurmantche | 15 | 15 | 15 | 15 | [^69^](#_ENREF_69) |  |
| Gurunsi | 16 | 16 | 16 | 16 | [^69^](#_ENREF_69) |  |
| Juhoansi | 18 | 18 | 18 | 17 | [^70^](#_ENREF_70) |  |
| Kanembu | 5 | 5 | 5 | - | [^69^](#_ENREF_69) |  |
| Karretjie | 20 | 20 | 20 | 17 | [^70^](#_ENREF_70) |  |
| Khomani | 39 | 39 | 39 | 39 | [^70^](#_ENREF_70) |  |
| Khwe | 17 | 11 | 17 | - | [^70^](#_ENREF_70) |  |
| Luhya | 33 | 33 | 33 | - | [^71^](#_ENREF_71) |  |
| Mende | 29 | 29 | 29 | 29 | [^71^](#_ENREF_71) |  |
| Mossi | 17 | 17 | 17 | 17 | [^69^](#_ENREF_69) |  |
| Nama | 20 | 20 | 20 | 20 | [^70^](#_ENREF_70) |  |
| Nubian | 14 | 14 | 14 | 14 | [^69^](#_ENREF_69) |  |
| Oromo | 14 | 14 | 14 | 14 | [^69^](#_ENREF_69) |  |
| Samburu | 10 | 10 | 10 | 10 | [^69^](#_ENREF_69) |  |
| Southeast Bantu | 20 | 20 | 20 | - | [^70^](#_ENREF_70) |  |
| Somali + Turkana | 14 (8 + 6) | 14 (8 + 6) | 14 (8 + 6) | 7 (5 + 2) | [^69^](#_ENREF_69) |  |
| Songhai | 3 | 3 | 3 | - | [^69^](#_ENREF_69) |  |
| Southwest Bantu | 12 | 12 | 12 | - | [^70^](#_ENREF_70) |  |
| Xun | 19 | 19 | 19 | 19 | [^70^](#_ENREF_70) |  |
| Yoruba | 36 | 36 | 36 | 36 | [^71^](#_ENREF_71) |  |

**
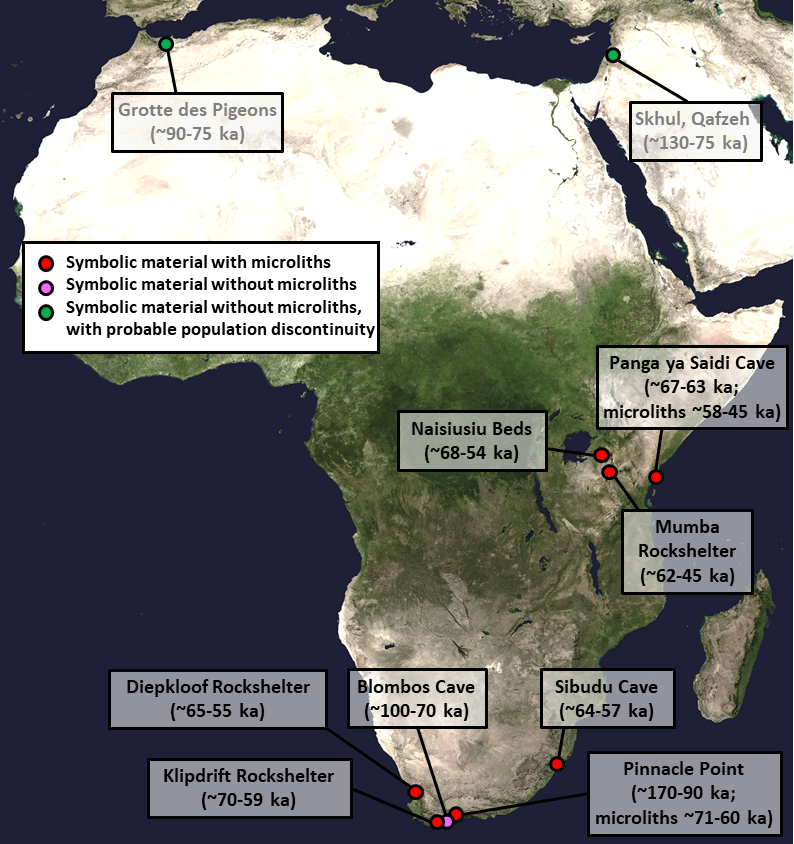
**

**Figure S1.** Location and approximate age of MSA sites with evidence for symbolic material and microliths mentioned in the main text and in the supplementary material. Sites in the Levant and Morocco are known from genetic evidence to have experienced replacement within the last ~50 ka. Base map is freely available from http://visibleearth.nasa.gov/view.php?id=57752 (NASA Goddard Space Flight Center Image by Reto Stöckli).


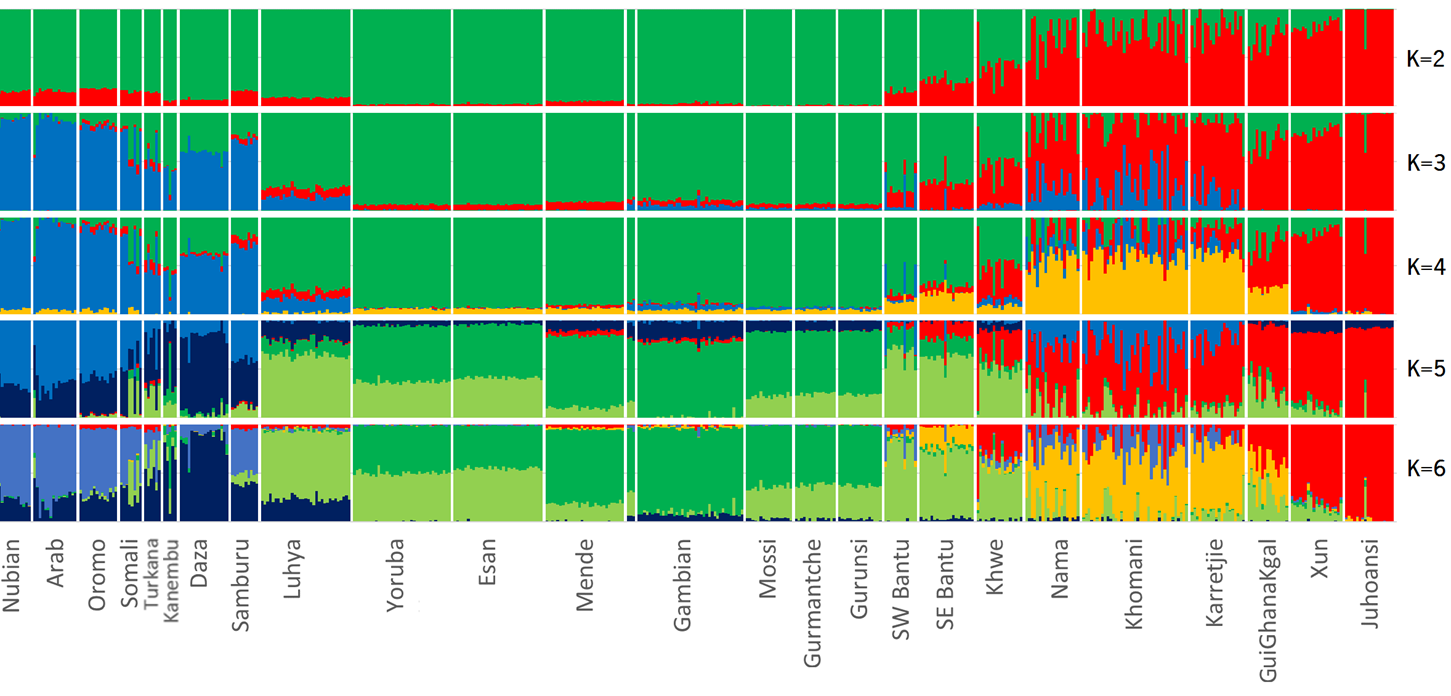


**Figure S2.** Reconstruction of ancestry in African populations using ADMIXTURE considering two to six ancestral populations (*K*= 2 to 6). Estimated best *K* corresponds to *K*=4.


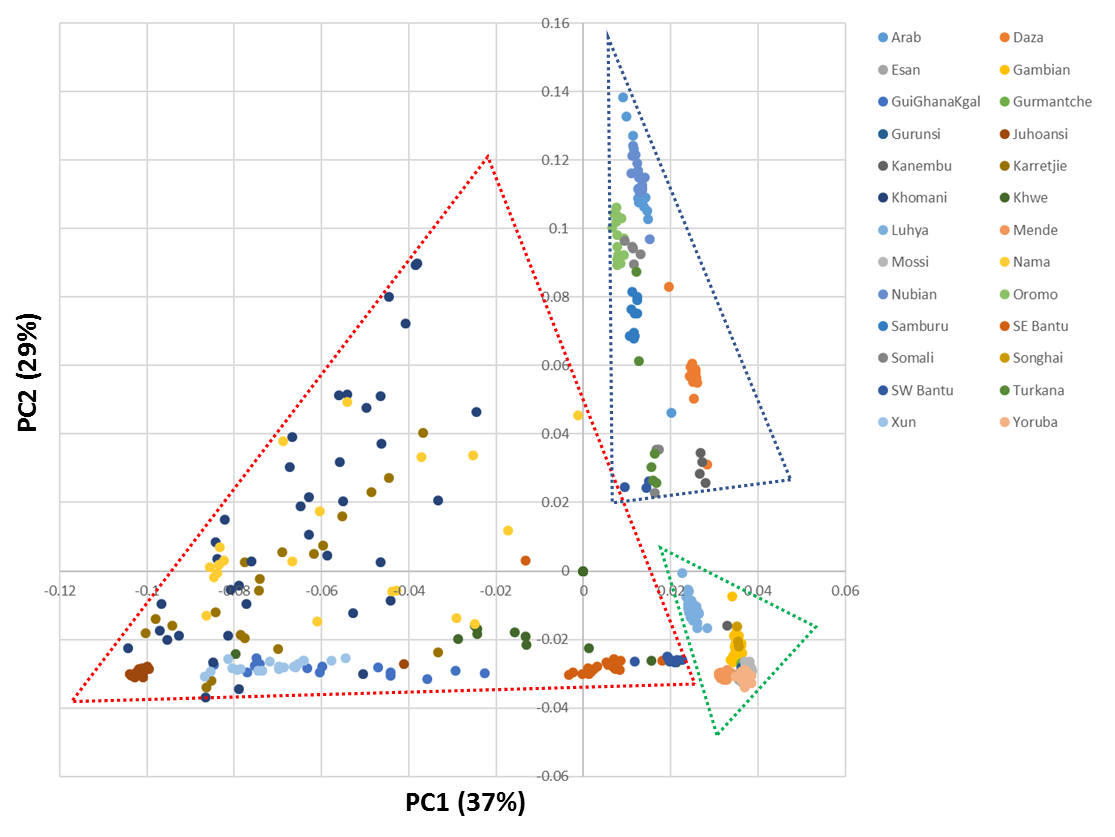


**Figure S3.** Principal component analysis of African populations plotting the two major components obtained. Triangles in dotted lines indicate southern Africa (red), eastern Africa (blue) and central/western Africa (green).

**References**

1. O'Shea JJ. Refuting a myth about human origins. *American Scientist* **99**, 128-135 (2011).

2. Higham T*, et al.* Chronology of the Grotte du Renne (France) and implications for the context of ornaments and human remains within the Châtelperronian. *Proc Natl Acad Sci U S A* **107**, 20234-20239 (2010).

3. Brown KS*, et al.* An early and enduring advanced technology originating 71,000 years ago in South Africa. *Nature* **491**, 590-593 (2012).

4. Marean CW*, et al.* Early human use of marine resources and pigment in South Africa during the Middle Pleistocene. *Nature* **449**, 905-908 (2007).

5. Henshilwood CS, d'Errico F, Watts I. Engraved ochres from the Middle Stone Age levels at Blombos Cave, South Africa. *J Hum Evol* **57**, 27-47 (2009).

6. Botha, Knight C. (eds). The cradle of language. Oxford University Press (2009).

7. Mellars P, Gori KC, Carr M, Soares PA, Richards MB. Genetic and archaeological perspectives on the initial modern human colonization of southern Asia. *Proc Natl Acad Sci U S A* **110**, 10699-10704 (2013).

8. Mackay A. Technological change and the importance of variability: The Western Cape of South Africa from MIS 6-2. In: *Africa from MIS 6-2: Population Dynamics and Paleoenvironments* (eds Jones SC, Stewart BA). Springer Netherlands (2016).

9. Soriano S*, et al.* The Still Bay and Howiesons Poort at Sibudu and Blombos: Understanding Middle Stone Age technologies. *PLoS One* **10**, e0131127 (2015).

10. Henshilwood CS, d’Errico F, van Niekerk KL, Dayet L, Queffelec A, Pollarolo L. An abstract drawing from the 73,000-year-old levels at Blombos Cave, South Africa. *Nature* **562**, 115–118 (2018).

11. d’Errico F, Vanhaeren M. Earliest personal ornaments and their signicance for the origin of language debate. In: *The cradle of language* (eds Botha R, Knight C). Oxford University Press (2009).

12. Henshilwood CS*, et al.* A 100,000-year-old ochre-processing workshop at Blombos Cave, South Africa. *Science* **334**, 219-222 (2011).

13. Jacobs Z, Roberts RG. Single-grain OSL chronologies for the Still Bay and Howieson's Poort industries and the transition between them: Further analyses and statistical modelling. *J Hum Evol* **107**, 1-13 (2017).

14. Mourre V, Villa P, Henshilwood CS. Early use of pressure flaking on lithic artifacts at Blombos Cave, South Africa. *Science* **330**, 659-662 (2010).

15. Henshilwood CS*, et al.* Klipdrift Shelter, southern Cape, South Africa: preliminary report on the Howiesons Poort layers. *JAS* **45**, 284-303 (2014).

16. Compton JS. Pleistocene sea-level fluctuations and human evolution on the southern coastal plain of South Africa. *Quat Sci Rev* **30**, 506-527 (2011).

17. Douze K, Delagnes A, Wurz S, Henshilwood CS. The Howiesons Poort lithic sequence of Klipdrift Shelter, southern Cape, South Africa. *PLoS One* **13**, e0206238 (2018).

18. Mellars P, Dark P. *Star Carr in context: new archaeological and palaeoecolgical investigations at the early Mesolithic site of Star Carr, North Yorkshire*. McDonald Institute for Archaeological Research (1998).

19. Clark JGD. *The earlier Stone Age settlement of Scandinavia*. Cambridge University Press (1975).

20. Clark J D. A comparison of the Late Acheulian industries of Africa and the Middle East. In: *After the australopithecines: Stratigraphy, ecology and culture change in the Middle Pleistocene* (eds KW Butzer, GL Isaac)*.* Aldine (1975).

21. Blome MW, Cohen AS, Tryon CA, Brooks AS, Russell J. The environmental context for the origins of modern human diversity: A synthesis of regional variability in African climate 150,000–30,000 years ago. *J Hum Evol* **62**, 563-592 (2012).

22. Texier P-J*, et al.* A Howiesons Poort tradition of engraving ostrich eggshell containers dated to 60,000 years ago at Diepkloof Rock Shelter, South Africa. *Proceedings of the National Academy of Sciences* **107**, 6180-6185 (2010).

23. Gliganic LA, Jacobs Z, Roberts RG, Domínguez-Rodrigo M, Mabulla AZP. New ages for Middle and Later Stone Age deposits at Mumba rockshelter, Tanzania: Optically stimulated luminescence dating of quartz and feldspar grains. *J Hum Evol* **62**, 533-547 (2012).

24. Skinner AR, Hay RL, Masao F, Blackwell BAB. Dating the Naisiusiu Beds, Olduvai Gorge, by electron spin resonance. *Quat Sci Rev* **22**, 1361-1366 (2003).

25. Tryon CA, Faith JT. Variability in the Middle Stone Age of eastern Africa. *Curr Anthrop* **54**, S234-S254 (2013).

26. Diez-Martín F*, et al.* The Middle to Later Stone Age Technological Transition in East Africa. New data from Mumba Rockshelter Bed V (Tanzania) and their implications for the origin of modern human behavior. *Journal of African Archaeology* **7**, 147-173 (2009).

27. Scholz CA*, et al.* East African megadroughts between 135 and 75 thousand years ago and bearing on early-modern human origins. *Proc Natl Acad Sci U S A* **104**, 16416-16421 (2007).

28. Lee RB, DeVore I. *Man the hunter*. Aldine (1968).

29. Backwell L, d'Errico F, Wadley L. Middle Stone Age bone tools from the Howiesons Poort layers, Sibudu Cave, South Africa. *JAS* **35**, 1566-1580 (2008).

30. Deacon HJ, Deacon J. *Human beginnings in South Africa: Uncovering the secrets of the Stone Age*. D. Phillips (1999).

31. Powell A, Shennan S, Thomas MG. Late Pleistocene demography and the appearance of modern human behavior. *Science* **324**, 1298 (2009).

32. Hudson RR. Generating samples under a Wright-Fisher neutral model of genetic variation. *Bioinformatics* **18**, 337-338 (2002).

33. Fay JC, Wu CI. Hitchhiking under positive Darwinian selection. *Genetics* **155**, 1405-1413 (2000).

34. Sokal R, Rohlf J. *Biometry: The principles and practices of statistics in biological research*. W. H. Freeman (1994).

35. Belle EMS, Ramakrishnan U, Mountain JL, Barbujani G. Serial coalescent simulations suggest a weak genealogical relationship between Etruscans and modern Tuscans. *Proc Natl Acad Sci U S A* **103**, 8012-8017 (2006).

36. Zlojutro M, Tarskaia LA, Sorensen M, Snodgrass JJ, Leonard WR, Crawford MH. Coalescent simulations of Yakut mtDNA variation suggest small founding population. *Amer J Phys Anthrop* **139**, 474-482 (2009).

37. Ingman M, Kaessmann H, Paabo S, Gyllensten U. Mitochondrial genome variation and the origin of modern humans. *Nature* **408**, 708-713 (2000).

38. Maca-Meyer N, González AM, Larruga JM, Flores C, Cabrera VM. Major genomic mitochondrial lineages delineate early human expansions. *BMC Genet* **2**, 13-13 (2001).

39. Arnason U, Gullberg A, Janke A, Kullberg M. Mitogenomic analyses of caniform relationships. *Mol Phylogen Evol* **45**, 863-874 (2007).

40. Mishmar D*, et al.* Natural selection shaped regional mtDNA variation in humans. *Proc Natl Acad Sci U S A* **100**, 171-176 (2003).

41. Macaulay V*, et al.* Single, rapid coastal settlement of Asia revealed by analysis of complete mitochondrial genomes. *Science* **308**, 1034-1036 (2005).

42. Just RS, Diegoli TM, Saunier JL, Irwin JA, Parsons TJ. Complete mitochondrial genome sequences for 265 African American and U.S. “Hispanic” individuals. *Forensic Sci Int Genet* **2**, e45-e48 (2008).

43. Torroni A, Achilli A, Macaulay V, Richards M, Bandelt H-J. Harvesting the fruit of the human mtDNA tree. *Trends Genet* **22**, 339-345 (2006).

44. Gonder MK, Mortensen HM, Reed FA, de Sousa A, Tishkoff SA. Whole-mtDNA genome sequence analysis of ancient African lineages. *Mol Biol Evol* **24**, 757-768 (2007).

45. Behar DM*, et al.* Counting the Founders: The matrilineal genetic ancestry of the Jewish diaspora. *PLoS One* **3**, e2062 (2008).

46. Behar DM*, et al.* The dawn of human matrilineal diversity. *Am J Hum Genet* **82**, 1130-1140 (2008).

47. Hartmann A*, et al.* Validation of microarray-based resequencing of 93 worldwide mitochondrial genomes. *Hum Mutat* **30**, 115-122 (2009).

48. Kujanová M, Pereira L, Fernandes V, Pereira JB, Černý V. Near Eastern Neolithic genetic input in a small oasis of the Egyptian Western Desert. *Amer J Phys Anthrop* **140**, 336-346 (2009).

49. Eaaswarkhanth M*, et al.* Traces of sub-Saharan and Middle Eastern lineages in Indian Muslim populations. *Europ J Hum Genet* **18**, 354-363 (2009).

50. Batini C*, et al.* Insights into the demographic history of African Pygmies from complete mitochondrial genomes. *Mol Biol Evol* **28**, 1099-1110 (2011).

51. Barbieri C, Whitten M, Beyer K, Schreiber H, Li M, Pakendorf B. Contrasting maternal and paternal histories in the linguistic context of Burkina Faso. *Mol Biol Evol* **29**, 1213-1223 (2012).

52. Behar Doron M*, et al.* A “Copernican” reassessment of the human mitochondrial dna tree from its root. *Am J Hum Genet* **90**, 675-684 (2012).

53. Barbieri C, Butthof A, Bostoen K, Pakendorf B. Genetic perspectives on the origin of clicks in Bantu languages from southwestern Zambia. *Europ J Hum Genet* **21**, 430-436 (2013).

54. Barbieri C, Vicente M, Rocha J, Mpoloka Sununguko W, Stoneking M, Pakendorf B. Ancient substructure in early mtDNA lineages of Southern Africa. *Am J Hum Genet* **92**, 285-292 (2013).

55. van der Walt EM*, et al.* Characterization of mtDNA variation in a cohort of South African paediatric patients with mitochondrial disease. *Europ J Hum Genet* **20**, 650-656 (2012).

56. Barbieri C*, et al.* Unraveling the complex maternal history of Southern African Khoisan populations. *Amer J Phys Anthrop* **153**, 435-448 (2014).

57. Rito T*, et al.* The first modern human dispersals across Africa. *PLoS One* **8**, e80031 (2013).

58. Barbieri C*, et al.* Migration and interaction in a contact zone: mtDNA variation among Bantu-speakers in Southern Africa. *PLoS One* **9**, e99117 (2014).

59. Chan EKF*, et al.* Revised timeline and distribution of the earliest diverged human maternal lineages in Southern Africa. *PLoS One* **10**, e0121223 (2015).

60. Just RS*, et al.* Full mtGenome reference data: Development and characterization of 588 forensic-quality haplotypes representing three U.S. populations. *Forensic Sci Int Genet* **14**, 141-155 (2015).

61. Vyas DN, Kitchen A, Miró-Herrans AT, Pearson LN, Al-Meeri A, Mulligan CJ. Bayesian analyses of Yemeni mitochondrial genomes suggest multiple migration events with Africa and Western Eurasia. *Amer J Phys Anthrop* **159**, 382-393 (2016).

62. Heinz T*, et al.* The genomic legacy of the Transatlantic Slave Trade in the Yungas Valley of Bolivia. *PLoS One* **10**, e0134129 (2015).

63. Boettger LM*, et al.* Recurring exon deletions in the HP (haptoglobin) gene contribute to lower blood cholesterol levels. *Nat Genet* **48**, 359-366 (2016).

64. Quintana-Murci L*, et al.* Maternal traces of deep common ancestry and asymmetric gene flow between Pygmy hunter–gatherers and Bantu-speaking farmers. *Proc Natl Acad Sci U S A* **105**, 1596-1601 (2008).

65. Costa MD, Cherni L, Fernandes V, Freitas F, Ammar el Gaaied AB, Pereira L. Data from complete mtDNA sequencing of Tunisian centenarians: Testing haplogroup association and the “golden mean” to longevity. *Mech Ageing Dev* **130**, 222-226 (2009).

66. Černý V, Fernandes V, Costa MD, Hájek M, Mulligan CJ, Pereira L. Migration of Chadic speaking pastoralists within Africa based on population structure of Chad Basin and phylogeography of mitochondrial L3f haplogroup. *BMC Evol Biol* **9**, 63 (2009).

67. Harich N*, et al.* The trans-Saharan slave trade - clues from interpolation analyses and high-resolution characterization of mitochondrial DNA lineages. *BMC Evol Biol* **10**, 138-138 (2010).

68. Soares P*, et al.* The expansion of mtDNA haplogroup L3 within and out of Africa. *Mol Biol Evol* **29**, 915-927 (2012).

69. Triska P, Soares P, Patin E, Fernandes V, Cerny V, Pereira L. Extensive admixture and selective pressure across the Sahel Belt. *Genome Biol Evol* **7**, 3484-3495 (2015).

70. Schlebusch CM*, et al.* Genomic variation in seven Khoe-San groups reveals adaptation and complex African history. *Science* **338**, 374 (2012).

71. Sudmant PH*, et al.* An integrated map of structural variation in 2,504 human genomes. *Nature* **526**, 75-81 (2015).
